# Identification of collaborative driver pathways in breast cancer

**DOI:** 10.1186/1471-2164-15-605

**Published:** 2014-07-17

**Authors:** Yang Liu, Zhenjun Hu

**Affiliations:** Bioinformatics Graduate Program and Department of Biomedical Engineering, Boston University, 24 Cummington Mall, Boston, MA 02215 USA

**Keywords:** Mutation, Driver gene, Driver pathway collaboration, Breast cancer, Subtype

## Abstract

**Background:**

An important challenge in cancer biology is to computationally screen mutations in cancer cells, separating those that might drive cancer initiation and progression, from the much larger number of bystanders. Since mutations are large in number and diverse in type, the frequency of any particular mutation pattern across a set of samples is low. This makes statistical distinctions and reproducibility across different populations difficult to establish.

**Results:**

In this paper we develop a novel method that promises to partially ameliorate these problems. The basic idea is although mutations are highly heterogeneous and vary from one sample to another, the processes that are disrupted when cells undergo transformation tend to be invariant across a population for a particular cancer or cancer subtype. Specifically, we focus on finding mutated pathway-groups that are invariant across samples of breast cancer subtypes. The identification of informative pathway-groups consists of two steps. The first is identification of pathways significantly enriched in genes containing non-synonymous mutations; the second uses pathways so identified to find groups that are functionally related in the largest number of samples. An application to 4 subtypes of breast cancer identified pathway-groups that can highly explicate a particular subtype and rich in processes associated with transformation.

**Conclusions:**

In contrast to previous methods that identify pathways across a set of samples without any further validation, we show that mutated pathway-groups can be found in each breast cancer subtype and that such groups are invariant across the majority of samples. The algorithm is available at http://www.visantnet.org/misi/MUDPAC.zip.

**Electronic supplementary material:**

The online version of this article (doi:10.1186/1471-2164-15-605) contains supplementary material, which is available to authorized users.

## Background

Large collaborative efforts, including the Cancer Genome Atlas (TCGA) [1] and the International Cancer Genome Consortium (ICGC) [2], are taking initial steps toward developing a blueprint of human cancer genomes by identifying, characterizing and cataloguing alterations in thousands of tumor samples. A major challenge in interpreting the data generated by these projects is to distinguish those mutations that play a role in the initiation and progression of cancer, from the much larger number of passenger alterations that play no role in cancer cell development [3].

Common approaches hypothesize that mutations conferring a selective advantage on tumor initiation and progression will occur at high frequency across a wide range of tumor samples. The useful implementation of this concept, however, faces a number of well know challenges. First, the vast majority of drivers occur only rarely, making them difficult to detect statistically [4]. Second, the number of different types of mutations and the number of altered genes are both very large. As a result, the sets of genes implicated by different studies often display relatively low overlap. This makes it difficult to establish a consistent causal mechanism for a given cancer [5]. Finally, transformed cells are typically mutated in multiple members of a set of functionally related genes. Consequently, mutations that drive transformation, especially when rare, are best sought and understood in that context [6]. This idea of a functionally coupled set of genes is not new, and it has been exploited in a number of studies aimed at discovering drivers.

One way to proceed is to identify known pathways that are enriched in genes carrying somatic mutations [1, 7]. More recent methods exploit genomic characteristics of mutations, such as mutual exclusivity, to identify oncogenic modules [8–10]. In addition, gene level knowledge -including gene size bias [11, 12], gene-interaction networks and expression levels [4, 6, 13, 14] - have been incorporated to uncover the mutational significance of a pathway. Copy number alteration [15] and other biological knowledge of mutational processes, such as transcript isoforms, variation in mutation type and redundancy of genetic code [16] are fully integrated into analysis. Algorithms that infer patient specific pathways have also been developed [17, 18].

However, very few methods involve a principled procedure for taking account of pathway interactions, i.e. pathways that are mutated in the same sample, and that are mutated together across a large subset of samples. We develop a two-step procedure - referred to as Mutational Driver Pathway Collaboration (MUDPAC) - for identifying groups of interactive pathways, and apply it to the analysis of 4 breast cancer subtypes.

The first step identifies candidate driver pathways using a mutational pathway enrichment analysis, based on a modification of Pathway Enrichment Analysis (PWEA) developed previously in our Lab [19]. Genes are ranked, as described in Methods, using a scoring function that combines a gene Mutation Factor (*MF*) and a gene Interaction Factor (*IF*). The *MF* scores a gene by a weighted difference between its non-synonymous and synonymous mutation rates. The *IF* takes account of mutational patterns, i.e., we allow the score of a mutated gene g* in the list to depend on the number and type of mutated genes (i.e., mutual exclusivity, topology distance) in its functional neighborhood, as explained in Methods. The ranked list of genes is analyzed in the usual way [20] to identify pathways whose genes are significantly enriched in the highest scoring candidates.

The second step searches the identified pathways for combinations that are most informative about a particular cancer subtype, assuming that pathways involved in producing a distinct cancer phenotype will tend to be cooperative, i.e., they will be simultaneously aberrant in the majority population of samples. The main idea here is that patients with the same cancer subtype will have some common set of perturbed cellular functions.

We applied the method to 29,900 somatic mutations identified in 11,897 genes from 498 breast cancer (BRCA) patients distributed over four subtypes annotated in the Cancer Genome Atlas (TCGA) project [5]: Basal-like (93), HER2+ (57), Luminal-A (224), Luminal-B (124).

## Results

### Overview of MUDPAC

**Figure 1:**
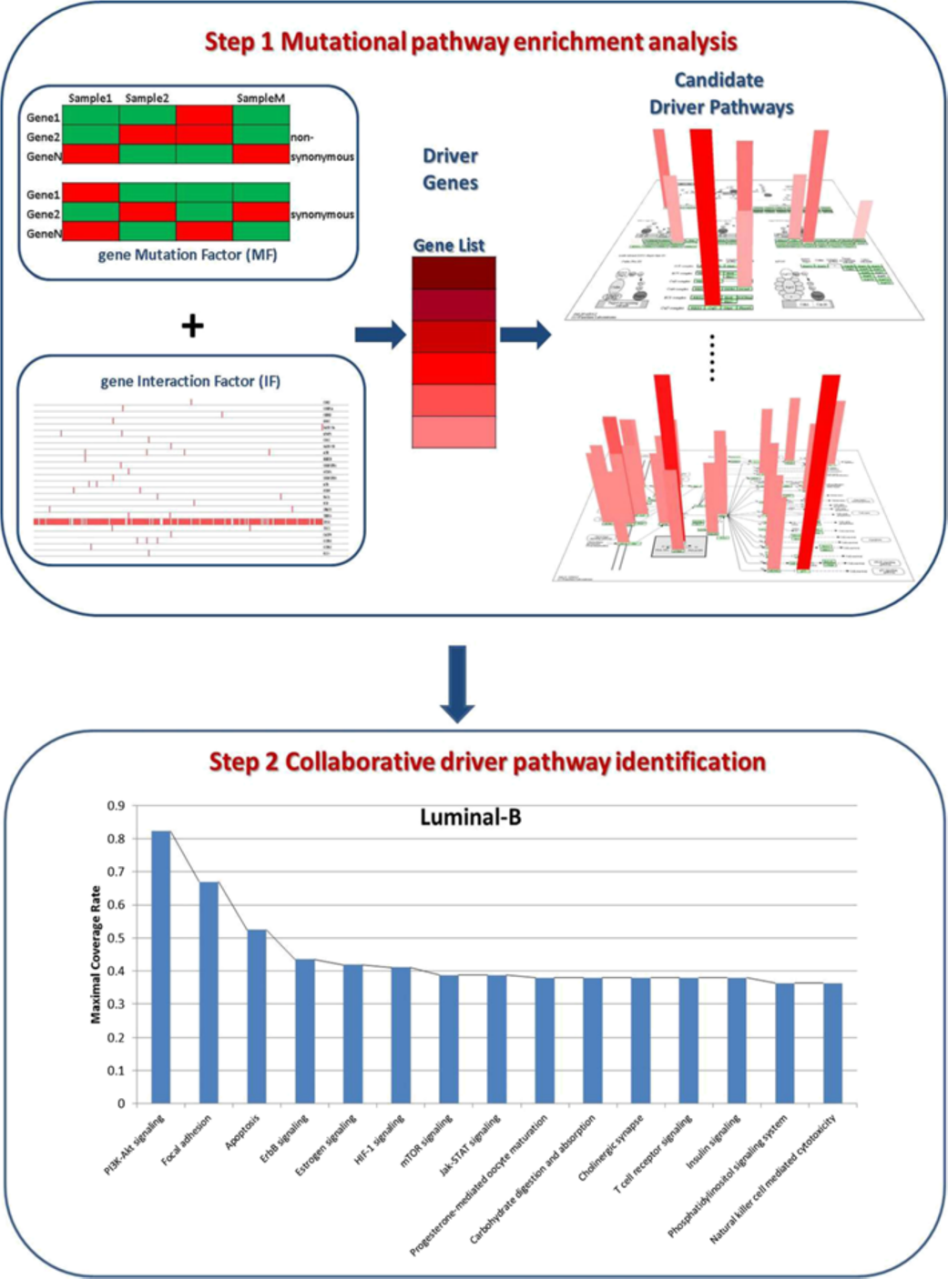
**Schematic representation of MUDPAC.** In Step 1 a ranking score is calculated as a function of Mutation Factor (*MF*) and Interaction Factor (*IF*). *MF* is essentially the difference between non-synonymous and synonymous mutational impacts averaged across all samples; *IF* takes account of the mutational landscape in the functional vicinity of the gene being scored. A ranking gene list is then computed based on the score assigned to each gene (darker red indicates higher score), which is then used to find candidate pathways. In Step 2, the coverage rates of the Top 60 candidate driver pathways are calculated based on the number of samples where there is at least one non-synonymous mutation in the corresponding pathway. Greedy algorithm is applied to find a set of pathways whose composition and pattern, uniquely identifies a breast cancer subtype in a maximum number of samples.

Details of the algorithm are described in Methods, while a brief introduction is summarized here, with a schematic representation shown in Figure 1. There are two hypotheses when identifying collaborative driver pathways. First, complex diseases such as cancers often result from cooperative perturbations in multiple functions or pathways. Second, any gene that disrupts a driver pathway is considered to be a driver candidate; therefore a driver pathway need not be dominated by the mutation of a single gene.

In the first step of MUDPAC we proceed in analogy to Gene Set Enrichment Analysis (GSEA) [20], which identifies pathways that correlate with phenotypic difference (tumor VS normal) based on gene’s differential expressions. For mutation data we use the differences between the non-synonymous mutations, and a background of synonymous mutations [7, 21]. Genes are ranked by using a score that integrates a mutation factor (*MF*), i.e., the difference between the impact of non-synonymous and synonymous mutations, and an Interaction Factor (*IF*), which takes account of the mutational landscape in the functional vicinity (for example, propinquity on a pathway) of a particular gene. Pathways whose genes are significantly overrepresented toward the top of the ranked list are then identified as candidate driver pathways.

The second step searches the identified pathways in Step 1 for combinations that are most informative about a particular cancer subtype, assuming that driver pathways should collaborate to achieve a distinct phenotype. The main idea here is that patients with the same cancer subtype will have the same set of disrupted cellular functions. A greedy algorithm is used to find the single pathway that is mutated in (covers) the largest number of samples, and then iteratively adds new pathways to form a collaborative group that achieves a Maximal Coverage Rate (MCR), which is the percentage of samples covered by current set of driver pathways. This is done subject to the restrictions that (i) the collaboration (mutation co-occurrence) between a new pathway and all previously selected pathways is statistically significant (*P* < 0.01) in comparison to the case where mutations of the genes in the new pathway are distributed randomly (see Methods for details) across the samples; (ii) the new MCR after the addition of the new pathway is at least 5% (See Additional file 1 for detailed explanation) higher than the mutation coverage of samples of any single gene in the new pathway. This is to ensure that the functional disruption of the pathway is not dominated by the mutation of a single gene.

Detailed results of the Top 60 selected pathways from Step 1, pathway collaborations along with MCR and *P*-values of each subtype resulted from Step 2, can be found in Additional file 2.

### Application to breast cancer

#### Summary of results

**Figure 2:**
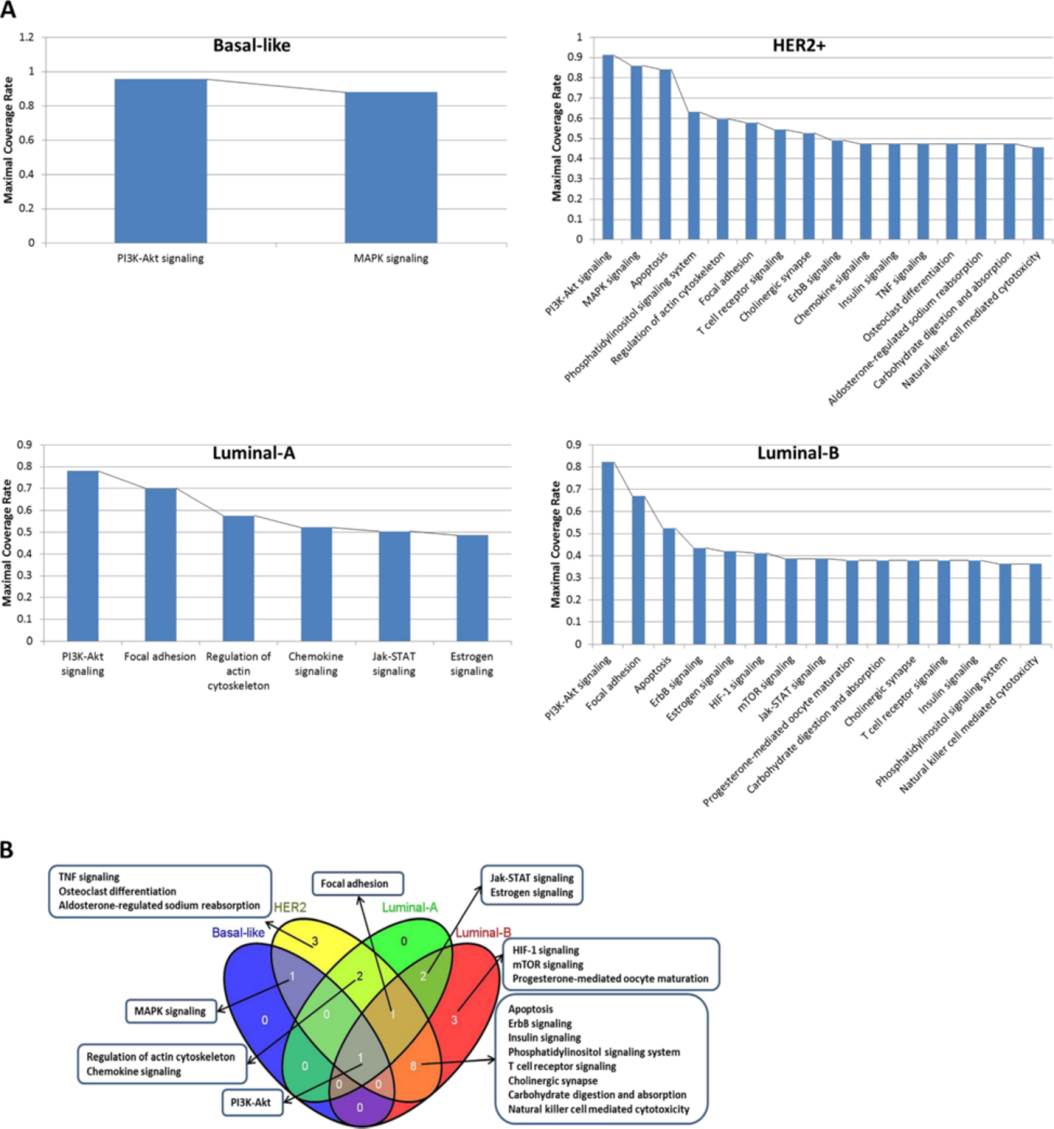
**Driver pathway collaboration in breast cancer. (A)** Identified collaborative driver pathways in each of the 4 subtypes. Mutational pathway enrichment analysis is performed on the 4 subtypes separately, and collaborative driver pathway identification is applied over the Top 60 significantly enriched pathways. The numbers of driver pathways identified in subtype of Basal-like, HER2+, Luminal-A and Luminal-B are 2, 16, 6, 15 respectively. **(B)** Collaborative driver pathway venn diagram.

We refer to a pathway as mutated in a given sample if the pathway has at least one non-synonymous mutation in that sample; the sample is then said to be covered by the pathway. A set of pathways are collaborative in a given sample if each member of the set is mutated. Using these definitions and the results in Step 1, we iteratively search for a set of collaborative pathways for each subtype (Figure 2A), such that MCR is achieved -- with additional restrictions as explained in Methods. Briefly, we proceed as follows. To be concrete, we frame the description in terms of Luminal-A.

We started with the Top 60 pathways obtained in Step 1, and identified the pathway that has maximum coverage rate, which turns out to be PI3k-Akt. The MCR of PI3k-Akt pathway is 78% and *PIK3CA* is the gene that has the highest mutation rate in this pathway (44%). PI3k-Akt is therefore selected as the first pathway of the collaborative set because the MCR of the pathway is much higher than the single gene mutation rate (threshold = 5%). We then searched for the next pathway that has the largest coverage rate in the samples covered by the collaborative pathway set. That turns out to be the focal adhesion pathway. The MCR of these two pathways is 70%, and the gene with the highest mutation rate in the focal adhesion pathway is still *PIK3CA*, therefore the MCR is again much higher than the mutation rate of any single gene in the focal adhesion pathway. In addition, the collaboration between two pathways with an MCR 70% is statistically significant based on 5000 permutation test by randomly distributing the mutations of the genes in focal adhesion pathway across the different samples (*P* < 0.0002). The focal adhesion pathway is therefore selected as part of the set. The search procedure continues interactively until no qualified pathways can be found.As shown in Figure 2A, MCR in general drops quickly at the beginning and becomes flat when more pathways are added to the collaborative set. The plateau after the drop is what we expected based on our hypothesis that disrupted functions/pathways tend to be invariant across samples. We also found that heterogeneous subtypes tend to have larger numbers of collaborative pathways and lower MCRs.

The Basal-like subtype, which is dominated by *TP53* (mutation rate 82%), is the most homogeneous subtype in breast cancer, so there are only 2 collaborative pathways selected with MCR = 88%. The luminal subtypes, on the other hand, are reported as heterogeneous, with the largest number of mutated genes but low overall mutation rates [5]. This shows a marked consistence in our results where *PIK3CA* is the highest mutated gene in both Luminal-A and Luminal-B with mutation rates of 44% and 31% respectively, and Luminal-B includes 15 collaborative pathways with MCR as low as 36%. Luminal-A, however, is not as obviously heterogeneous as Luminal-B, with only 6 pathways identified and MCR equal to 49%. This might explain its better prognosis and lower relapse rate in comparison with Luminal-B.

HER2+ is closely related to Luminal-B. Previous studies found that about 50% of clinically HER2+ tumors are observed predominantly in luminal mRNA subtypes [5], and HER2+ tumors are classified molecularly as Luminal-B in other studies [22]. Our results also show close overlaps between these two subtypes: there are 16 collaborative pathways selected in HER2+ with an MCR of 46%, which are quite similar with Luminal-B, not only in the number of selected pathways, but also in the overlap of those collaborative pathways. Pathways in HER2+ are a mixture of *TP53* (mutation rate 72%) related pathways and *PIK3CA* (mutation rate 40%) related pathways.

A list of driver pathways identified in each of the subtypes can be seen in Table 1. The common pathway in the collaborative pathway sets of the 4 subtypes is “PI3K-Akt signaling” (hsa04151), as shown in Figure 2B. PI3K is known to play an important role in breast tumor progression [23–26]. Our results suggest that it is the single most informative pathway for breast cancer. In particular it has significantly enriched genes in a higher percentage of samples than any other pathway, the percentages being 96, 91, 78, 82 in Basal-like, HER2+, Luminal-A, Luminal-B respectively. It is also the only pathway with significant mutational enrichment in the majority of samples in all subtypes.

**Table 1:** **List of driver pathways in each subtype**

Subtype	Driver pathway
Basal-like	PI3K-Akt signaling; MAPK signaling
HER2+	PI3K-Akt signaling; MAPK signaling, Apoptosis; Phosphatidylinositol signaling system; Regulation of actin cytoskeleton; Focal adhesion; T cell receptor signaling; Cholinergic synapse; ErbB signaling; Chemokine signaling; Insulin signaling; TNF signaling; Osteoclast differentiation; Aldosterone-regulated sodium reabsorption; Carbohydrate digestion and absorption; Natural killer cell mediated cytotoxicity
Luminal-A	PI3K-Akt signaling; Focal adhesion; Regulation of actin cytoskeleton; Chemokine signaling; Jak-STAT signaling; Estrogen signaling
Luminal-B	PI3K-Akt signaling; Focal adhesion; Apoptosis; ErbB signaling; Estrogen signaling; HIF-1 signaling; mTOR signaling; Jak-STAT signaling; Progesterone-mediated oocyte maturation; Carbohydrate digestion and absorption; Cholinergic synapse; T cell receptor signaling; Insulin signaling; Phosphatidylinositol signaling system; Natural killer cell mediated cytotoxicity

**Figure 3:**
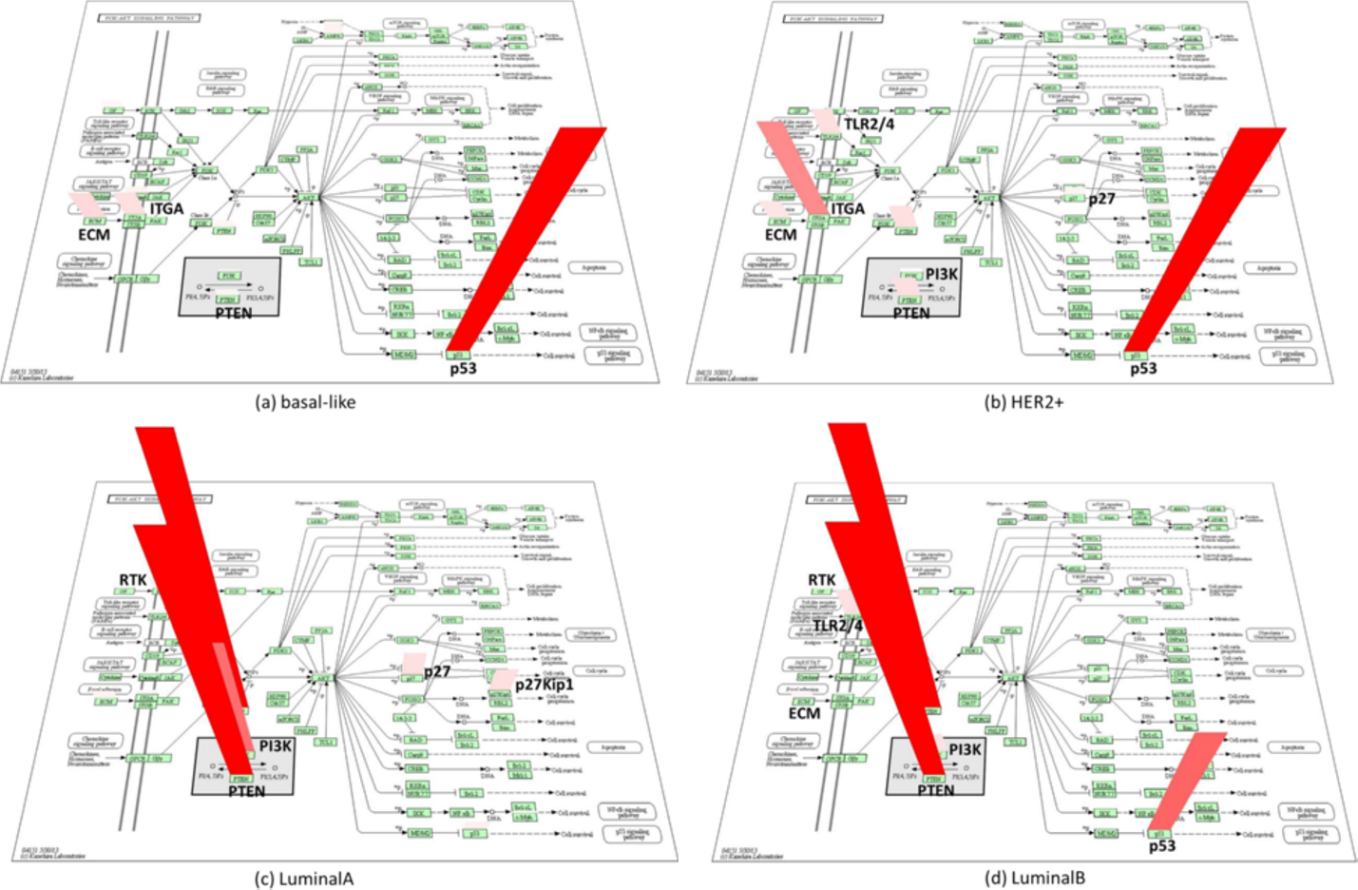
**Gene ranking score distribution of PI3K pathway in different subtypes.** The ranking scores of each gene (in log scale) in PI3K pathway are displayed as a red bar in the 3D plot using KEGG Color Pathway 3D, with the color intensity and bar height proportional to the gene’s ranking score. Genes with high ranking scores are labelled. **(a)** basal-like subtype. **(b)** HER+ subtype. **(c)** Luminal-A subtype. **(d)** Luminal-B subtype.

Although the PI3K-Akt signaling pathway is common to all subtypes, the significantly mutated genes of this pathway in different subtypes are quite different, as shown in Figure 3. All the genes within this pathway were drawn in a 3D plot using Kyoto Encyclopedia of Genes and Genomes (KEGG) Color Pathway 3D, with its ranking scores proportional to the color intensity and height of the red bar, and darker red means higher gene ranking score. We can see that Basal-like subtype is predominant of *TP53*, HER2+ subtype is a series function of *TP53, ITGAV, PTEN, TLR4, COL5A3, CDKN1B* and *PIK3R1*, in descending order of the gene score. In Luminal-A subtype, *PTEN* and *PIK3CA* are the most important genes while *CDKN1B* and *KIT* also play roles in perturbing this pathway. Luminal-B is mainly affected by *PTEN* and *TP53*, but *TLR4, PIK3CA, KIT* and *LAMA5* also contribute to functional disruption.

For the genes mentioned above, *TP53, PTEN, PIK3R1, PIK3CA, KIT* are all oncogenes or tumor suppressing genes that have records in the Catalogue of Somatic Mutations In Cancer (COSMIC) (http://cancer.sanger.ac.uk/cancergenome/projects/cosmic/) or the Online Mendelian Inheritance in Man (OMIM) (http://www.omim.org/), and many of the genes have been reported to be associated with cancer or other diseases. For example, it is proposed that protein encoded by *ITGAV* interacts with several extracellular matrix proteins to mediate cell adhesion and may play a role in cell migration. This protein may also regulate angiogenesis and cancer progression [27]. The protein encoded by *CDKN1B* is found to bind to and prevent the activation of cyclin E-CDK2 or cyclin D-CDK4 complexes, therefore controls the cell cycle progression at G1 and its polymorphism appears to be an important predictive factor for breast cancer risk [28].

The KEGG Color Pathway 2D diagrams for every driver pathway of all the 4 subtypes can be seen in Additional File 1, with gene’s ranking score proportional to the color intensity of the red box representing that gene, and darker red mean higher gene ranking score.

#### Basal-like subtype

The driver pathway set in the basal-like subtype includes pathways of “PI3K-Akt” and “MAPK signaling”, with a MCR of 88%. As shown in Figure 2B, no unique pathways are discovered in this subtype. Basal-like tumors showed a high frequency of *TP53* mutations, suggesting the loss of *TP53* function occurs within most of basal-like cancers [5]. Additional studies further support the idea that both pathways play important roles in basal-like breast cancer [29, 30]. Although the small number of driver pathway set may mainly be due to the homogeneity of the samples for this subtype, it may also results from the potential artifacts of our method that will be discussed in detail in the Discussion section.

#### HER2 positive subtype

A pathway collaboration of size 16 was identified with a MCR of 46% in this subtype. Most of these pathways are associated with two genes: the mutational enrichment of pathways such as “ErbB signaling” [31] and “Focal adhesion” [32], are subtype-representative because they are all associated with the *HER2* gene; while the enrichment of *TP53* associated pathways, including “PI3K-Akt signaling” [33] and “MAPK signaling” [34], probably result from the high mutation rate and toxic mutational function of *TP53.* They are shared with basal-like subtype.

The HER2+ subtype shares most of its driver pathways with luminal subtype, especially Luminal-B: not only the number and pattern of MCR of driver pathway collaborations, but also the involved pathways. This agrees with the clinical-pathological view that in general it may not be necessary to divide samples with *ERBB2* amplification into Luminal B and HER2+ [22].

The exclusive pathways in this subtype are “TNF signaling”, “Osteoclast differentiation” and “Aldosterone-regulated sodium reabsorption”. The TNF signaling pathway induces a wide range of intracellular signaling pathways including apoptosis and cell survival as well as inflammation and immunity. TNFα, which is expressed by nearly all cells and is the major receptor for TNF, can cross-talk with the EGFR/HER2 pathway at various points and affect the sensitivity to EGFR/HER2 inhibitors [35]. And overexpression of HER-2/neu induces resistance to TNF, which causes cancer cells to escape from host immune defenses [36]. For “Osteoclast differentiation”, there is evidence suggesting a likely association between the luminal subtype and bone metastasis [37]. Recent evidence suggests that bone metastasis also occurs in the HER2+ subtype [38], consistent with our mutational enrichment of the osteoclast differentiation pathway. This pathway is responsible for bone resorption and is mainly regulated by signaling pathways activated by *RANK* and immune receptors.

From a pathway category perspective, we found that besides categories of “Signal transduction” and “Cellular Processes” that are common among all subtypes, HER2+ has functional disruptions in “Organismal systems”, including “Immune system” (“T cell receptor signaling”, “Chemokine signaling”, “Natural killer cell mediated cytotoxicity”), “Nervous system” (“Cholinergic synapse”), “Endocrine system” (“Insulin signaling”), “Development” (“Osteoclast differentiation”), “Excretory system” (“Aldosterone-regulated sodium reabsorption”), and “Digestive system” (“Carbohydrate digestion and absorption”). A direct and complete relation of HER2+ with organismal systems has not been found, but some of the pathways above were indeed highlighted with their considerable connections, like “Insulin signaling” [39] and “Chemokine signaling” [40]. We expect the pathways that we identified can provide researchers a better understanding of the cross-coupling effects from these different systems.

Some of the pathway collaborations could be supported by the known drug resistance. Trastuzumab (Herceptin) is a clinically approved antibody for HER2-overexpression of breast cancer [41], but multiple HER2 cross-talk contributing to trastuzumab resistance has been reported. PI3K is the major downstream signaling pathway activated by HER2 cross-talk [42], and a potential integrator of receptor crosstalk is Src-focal adhesion kinase (FAK) signaling [43]. Another mechanism includes cross-signaling from related HER/erbB receptors and compensatory signaling from receptors of insulin-like growth factor-I [44]. The cross-talk effects involved above like “PI3K-Akt”, “ErbB signaling”, “Focal adhesion” and “Insulin signaling” are all reported in the driver pathway collaboration of our results, which may help to develop new pharmacological strategies to overcome the drug resistance.

#### Luminal-A subtype

**Figure 4:**
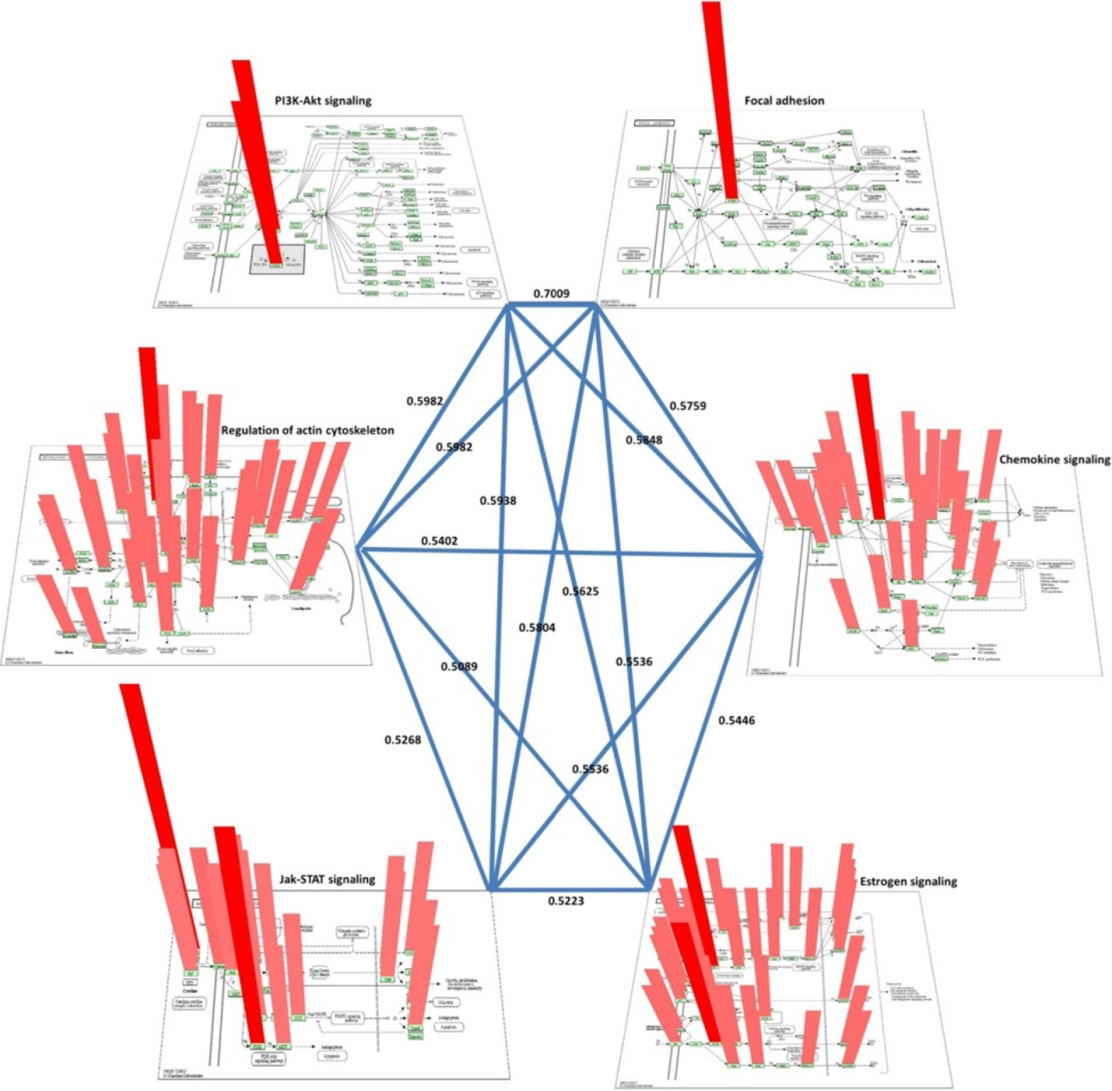
**Pathway collaboration of Luminal-A.** This figure is drawn with KEGG color pathway 3D. Gene scores (not log scaled) calculated from MUDPAC for each pathway are proportional to the length and color intensity of red bar. Two pathways are linked to each other with blue line, and edge width is proportional to the sample co-coverage percentage of these two pathways. The color of the red bars is drawn based on gene scores of a particular pathway only and are not comparable among different pathways. Genes with smaller values in a pathway are not able to be displayed if there are genes with much higher values in this pathway.

6 driver pathways have been identified with an MCR of 49%. There are no unique pathways in this subtype. In addition to the common pathway “PI3K-Akt signaling” that is present in all subtypes, there are 2 pathways shared with HER2+, another 2 pathways shared with Luminal-B and 1 pathway shared by both HER2+ and Luminal-B. The 2 pathways that only appear in luminal subtypes are “Estrogen signaling” and “Jak-STAT signaling”. The estrogen signaling pathway is a new pathway released by KEGG in 2013. It is not surprising that this pathway only appears in the luminal subtypes, as the luminal subtypes are characterized by the expression of genes activated by estrogen receptor transcription factor that are typically expressed in the luminal epithelium lining the mammary ducts [22]. The role of estrogen receptor (ER) in breast cancer has been widely studied [45–48].We demonstrated in Figure 4 how these 6 driver pathways work together, and how the genes within each of the pathways cooperate. Gene ranking scores calculated from our algorithm within each pathway are proportional to the length and color depth of the red bar. In order to show detailed gene score distribution of each pathway, we did not calculate log scale of ranking score here but use their original values. Two pathways are connected with each other based on their co-covered sample percentage; the width of each edge is proportional to the sample coverage percentage. This figure not only illustrates pathway collaboration by showing their coordinating relationships, but it also shows that mutations are well distributed across the genes in each pathway, as an example of our hypothesis that any genes with functional mutations can be driver genes.

#### Luminal-B subtype

The number of driver pathways in Luminal-B is 15 and they cover a MCR of 36% tumor samples. Compared with Luminal-A, Luminal-B always comes with a more difficult prognosis and a higher recurrence rate. A lot of efforts have been made to distinguish these two subtypes, here we focus on the differences at a driver pathway level. We found that both of these two subtypes are active in signal transduction, like “PI3K-Akt signaling”, “Jak-STAT signaling”, but Luminal-B tends to be more active with more mutated pathways in this category, including “HIF-1 signaling”, “mTOR signaling”, “ErbB signaling” and “Phosphatidylinositol signaling system”. “HIF-1 signaling” and “mTOR signaling” pathways are the unique pathways in Luminal-B. The hypoxia inducible factor-1 (HIF-1) is discovered to be interchangeable with estrogen as both similarly modulate epithelial-endothelial cell interaction [49], HIF-1α is associated with p21 but not against proliferation in ER positive tumors [50]. It is well established that PI3K/Akt/mTOR pathway plays a central role in resistance to endocrine therapy in breast cancer, partly through regulation of estrogen receptor α (ER) activity [51]. Phosphorylation of ER by mTOR’s downstream target p70S6K is potentially important in deciding personalized treatment for ER + breast cancers in the resistance to Tamoxifen [52].

Although both Luminal-A and Luminal-B are estrogen positive, Luminal-B seems to be more enriched in endocrine system pathways. Besides “Estrogen signaling”, there are two other endocrine related pathways in Luminal-B, “Progesterone-mediated oocyte maturation” and “Insulin signaling”. The “Progesterone-mediated oocyte maturation” pathway is exclusive to Luminal-B. The transition is accompanied by an increase in maturation promoting factor MPF or Cdc2/cyclin B, which is the main biological difference between two luminal subtypes found so far [53, 54]. Insulin/Insulin like growth factor 1 (IGF-I) and estrogens have potent positive effects on cell proliferation in breast cancer. Cross-talk between insulin-like growth factor (IGF) and estrogen receptor (ER) signaling pathways plays a critical role in breast carcinogenesis. The effects of ERα are mediated by the influences of insulin signaling pathway, and estrogens enhance insulin signaling by increasing the expression and/or the functional activity of some proteins involved in the insulin signaling pathway [55, 56]. Although the immune system is disrupted in both subtypes, the molecular basis of alteration appears to be subtype-specific: in Luminal-A it is a consequence of mutations in “Chemokine signaling”, while in Luminal-B the “T cell receptor signaling” and “Natural killer cell mediated cytotoxicity” pathways contribute the functional perturbations.

Cross-talk between estrogen receptor and growth factor has been discussed broadly to act as a molecular target for tamoxifen resistance [57, 58]. These growth factors include insulin-like growth factor-I (IGF1), which is found to act through a complex cross-talk with estrogen to stimulate the proliferation of normal mammary epithelium to increase the risk of breast cancer [59, 60]. The IGF1 involved pathways such as “PI3K-Akt signaling”, “HIF-1 signaling”, “mTOR signaling ”, “Focal adhesion” and “Progesterone-mediated oocyte maturation”, are all present in Luminal-B driver pathway collaboration, and 3 of them are even Luminal-B specific driver pathways, which is a strong support and complementation for our current understandings of ER + subtype, especially Luminal-B. Another important growth factor is the epidermal growth factor receptor 2 (HER2). Clinical evidence relates that treatment resistance to the presence of a complex bidirectional molecular crosstalk between the ER and HER2 pathways [61]. Treatment strategies that simultaneously block both signaling pathways have been proven to be promising in comparison with only targeting either one of them, which ultimately resulting in resistance to therapy.

#### Driver gene summary analysis

Finally, our results identify the key genes in all driver collaborative pathways for each subtype. In theory, we assume all the genes in driver pathway collaborations are with very high probability to be driver genes as they cooperate with each other to make a unique mechanism, but we are more interested at those genes with higher mutation scores. We ranked all the genes collected from driver pathway collaboration in each subtype according to their ranking score, and output Top 10 of them in ranking score descending order: Basal-like (TP53*, NF1*, LAMA5, ITGAV, NFKB2, PRKAA2, CACNA1B, LAMA1, IL2RB, PTEN), HER2+ (TP53*, CAPN2, ITGAV, ERBB3, PTEN*, ITPR3, ILK, TLR4*, COL5A3, DUSP4), Luminal-A (PTEN*, PIK3CA*, CDKN1B, LIFR*, KIT*, TP53*, COL5A3, ITGA6, ITGAV, TLR4*), Luminal-B (PTEN*, TP53*, PIK3CA*, TLR4*, KIT*, LIFR*, ACACA, LAMA5, ITPR3, VWF). There are 24 unique genes in the 4 different groups, 8 of them have overlap with genes in COSMIC or OMIM, marked with *, the remaining 16 of them also reflect potential relations to cancer, like *LAMA5* is implicated in a wide variety of biological processes including cell adhesion, differentiation, migration, signaling, neurite outgrowth and metastasis; overexpression of *ILK* is implicated in tumor growth and metastasis, which makes it an attractive target for cancer therapeutics [62]; amplification of *ERBB3* and/or overexpression of its protein have been reported in numerous cancers, including prostate, bladder, and breast [63, 64].

#### Comparison with other methods

For the mutational enriched pathways identified in step 1, we validated our results with other sources of mutated genes such as COSMIC and Cancer Cell Line Encyclopedia (CCLE) [65], and also did a comparison with Driver Genes and Pathways (DrGaP) [16], as detailed in Additional file 1.

Although a number of methods have been developed to identify driver pathways in cancer, including Mutual Exclusivity Modules (MEMo) [9], Mutational Significance in Cancer (MuSiC) [12], NetBox [15], Pathway Recognition Algorithm using Data Integration on Genomic Models (PARADIGM) [17], De novo Driver Exclusivity (Dendrix) [8], Mutated Driver Pathway Finder (MDPFinder) [14], HOTNET [13], DriverNet [6], Driver Genes and Pathways (DrGaP) [16], Multiple Pathway De novo Driver Exclusivity (Multi-Dendrix) [10], none of above focus on the collaborations among pathways on individual samples. Here we compared our method against one of them Multi-Dendrix [10], as both MUDPAC and Multi-Dendrix aim at determining driver pathway groups on the basis that mutations in several pathways, not in a single one, are generally required in cancer.

Multi-Dendrix recovers pathways from mutual exclusivity pattern of genes without any prior information about their interactions, while MUDPAC focuses on KEGG pathways to take account of the topological influence on our discoveries. The main drawback of Multi-Dendrix is that it can only find optimal solutions with limited number (2–4) of very small pathways (3–5 genes only) [10]. MUDPAC on the other hand, can select driver pathways with flexible size (2 for Basal-like subtype and 16 for HER2+ subtype). Again, although Multi-Dendrix identifies multiple driver pathways by maximizing the sum of sample coverage of each driver pathway, it does not require these pathways to be mutated in the same set of samples. MUDPAC to the opposite, tries to discover a collaborative pattern so that all pathways involved are disrupted in the same sample set. The pathways identified by MUDPAC represent functional disruptions among majority people (not individual one) of a given cancer and therefore provide more reliable insights about the mechanisms of corresponding tumorigenesis. The novel pathways identified by the collaborative pattern for each of the 4 subtypes complement our current picture of the emergence and progression of breast cancer, and may furthermore provide feasible, practical and customized therapies in clinical practice in the perspective of patient diagnose and personalized medicine.

For the 4 pathways that were found in BRCA by Multi-Dendrix, 2 of them also showed up in our results (PI3K-Akt signaling, MAPK signaling), the other two (p53 signaling, cell cycle) were selected as significantly mutated pathways in our Step 1 but filtered out in Step 2, as we found that the mutational contributions of these two pathways are dominated by *TP53*. For example, both p53 signaling and cell cycle are in the Top 60 significantly enriched pathways in the Basal-like subtype, but the MCRs of these two pathways are 80% and 81% respectively, comparable to and slightly less than the single gene mutation rate of *TP53* (82%). Cell cycle pathway is also enriched in HER2+ subtype whose MCR (72%) can’t meet the required 5% difference to the single gene mutation rate of *TP53* (72%).

We also compared the sample coverage of driver pathways for both methods. The highest and lowest sample coverage for Multi-Dendrix are 61% and 36% respectively, but 88% and 36% respectively for MUDPAC.

Finally, Multi-Dendrix is not designed to discover subtype-specific pathways while MUDPAC is aimed to identify subtype-specific collaborative set of pathways because it is built based on the hypothesis that different cancers are resulted from the different systematic failure of cellular functions. As clearly shown in Figure 2 and Table 1, the collaborative sets of pathways are very distinguishable despite the overlapping of individual pathways. As detailed in the section of each subtypes, most of them well represent the characteristics and mechanism of the corresponding subtype and provide new insights in the development of personalized medicine therapy.

## Discussion

MUDPAC identifies representative driver pathways for all 4 subtypes of breast cancer using TCGA data sets. The number of driver pathways in Basal-like, HER2+, Luminal-A and Luminal-B are 2, 16, 6 and 15 respectively. The common pathway for all subtypes is PI3K-Akt signaling. Some subtype-specific pathways are also found such as estrogen signaling pathway in both luminal subtypes and ErbB signaling pathway in both HER2+ and Luminal-B. Driver collaborative pathways in Basal-like are all associated with *TP53*; *ERBB2* related pathways and some *TP53* related pathways tend to be active in HER2+ tumors. In addition, HER2+ shares most of its characters with Luminal-B, not only the number and pattern of MCR of driver pathway collaborations, but also the involved pathways. Furthermore, HER2+ is the subtype that has the largest number of enriched pathways in organismal systems (total of 8). *PIK3CA* related pathways seem to play more important roles to drive the disease in luminal subtypes because most of these pathways appear in the corresponding driver pathway set. The distinction between Luminal-A and Luminal-B is that Luminal-B seems involving more pathways in the category of signal transduction, endocrine system, as well as immune system.The distributions of mutated genes within driver pathways, as well as their corresponding mutational frequencies, are also interesting. Figure 3 indicates that although the PI3K pathway is perturbed in all four subtypes, the way it is being perturbed can be very different, which may then lead to the different disruption of the same function. On the other hand, Figure 4 illustrates that it is very often that different genes in the same pathway are mutated in different samples, which not only explains why mutational gene signatures from different studies usually can’t agree each other but also indicates the urgent needs for personal therapies.

Several factors may impact our results, including single gene dominance, high mutation frequency and our limited knowledge of pathways. Some of them have been addressed in the current methods while others may need further development.

The single gene dominance points to the case where one gene has high mutation frequency in several different pathways, such as *TP53* and *PIK3CA* in our study. We take two steps to address this problem. First, the enrichment analysis filters out those pathways where only one or two genes have mutations; second, we require the MCR must be higher than the single gene mutation rate in any driver pathway by some threshold. By doing this, we still find that there are influences of single genes penetrating through collaborative pathways in each subtype, like pathways in Basal-like are all *TP53* related, pathways in Luminal-A and Luminal-B are all *PI3K* related, while pathways in HER2+ are a mixture of these two genes. Although the influences of these two genes in breast cancer have been well established [5], not all pathways related to these two genes show up in our results. Take *PIK3CA* and Luminal-A subtype as examples, there are totally 30 pathways including *PIK3CA* as input of our algorithm, 13 of them were selected as Top 60 in the first step of our method, which filtered out some pathways that are not highly enriched in mutations, and only 6 out of these 13 survived after Step 2, which adopted a more strict criteria to unearth the correlations among selected pathways. This indicates that our 2-step framework works efficiently to locate sets of distinguishable pathways for each subtype that are highly correlated with each other in the maximum number of samples. The collaborative pathway patterns for each subtype without considering single gene effects can be seen in Additional file 2, in which we simply applied greedy algorithm to select pathway with highest MCR each time, without requiring MCR 5% higher than single gene mutation rate and the significance of selected pathways.

Our method may fail if there are individual genes whose mutational rates are very high for a given cancer (e.g., ovarian cancer), because the statistical significance of pathway collaboration in our current method will be difficult to achieve. As a result, very few pathways may be identified as the set of driver pathways. The Basal-like subtype, the subtype of breast cancer that is most similar to ovarian cancer, may be impacted by both single gene dominance and high mutation frequency. There are a total of 7 pathways that consist of *TP53* in KEGG pathway database (excluding disease related pathways) as our input, 5 of them showed up in the Top 60 of Step 1 for this subtype, only 2 of them were considered as collaborative pathways in Step 2. The other 3 (“Wnt signaling”, “Cell cycle”, “p53 signaling”) are also important and related to cancer; and the reason they are not selected is that they are dominated by single gene effects with MCRs equal or lower than the mutation rate of *TP53*. In addition, they are not significantly correlated with 2 identified pathways.

Another limitation of MUDPAC is that present analysis is based on prior knowledge of known pathways in KEGG only. This is far from accurate if taking into account the incompleteness of human pathways or missing topology information. We need to make the definitions of this method feasible to a more general network. It could be possible to expand the pathway data set using other data sources, like Functional Linkage Network (FLN) [66] or Human Reference Network (HRN) [67].

One potential improvement of MUDPAC is to include inherited mutations when performing mutational pathway enrichment analysis. It is well known that for most sporadic cancers, somatic mutations are accumulated and restricted to an individual cell of the body through a human being’s lifespan. However, cancer also has heritable components. Inherited mutations in *BRCA1/BRCA2* for example, increase the risk of breast and ovarian cancer [68]. If heritable mutations are included in Step 1, the number of mutated pathways could be increased or the significance of existing enriched pathways could be strengthened. Furthermore in Step 2, the MCR of pathway groups could be possibly heightened, which may provide a more complete and ameliorated view of the mechanism of cancer.

It will also be useful to meliorate MUDPAC by integrating a larger panel of driving alterations beside the mutations, both genetic and epigenetic, such as somatic copy number alteration (SCNA), methylation and transcription factor (TF) etc. Identifying the oncogene and tumor suppressor gene targets of driver SCNAs and elucidate the functional roles of SCNAs have been considered important in cancer diagnostics and therapeutics. It is well recognized that the known oncogenes as well as novel ones involved in the significantly altered regions would enable researchers to identify new causes and molecular pathways that may one day be targeted to treat cancer [69]. It is also discovered that tumor suppressor genes are often subjected to silencing through cancer-specific promoter DNA methylation [70], and identification of driver and passenger DNA methylation in cancer may provide new insight about the process of carcinogenesis [71, 72]. Alterations of numerous transcription factors (TF), on the other hand, were shown to be causatively involved in various cancers in human [73], and identification of their roles will be useful in determining a more “holistic” picture of tumorigenesis and cancer treatment [74]. Overall, a flexible framework will be useful for MUDPAC to allow the different combination of these driving forces upon the availability of the corresponding data.

## Conclusions

MUDPAC identifies collaborative driver pathways in cancer using a two-step approach: mutational pathway enrichment analysis followed by greedy search for the collaborative driver pathways. The enrichment analysis examines whether a given pathway shows statistically significant differences between non-synonymous mutation group and synonymous mutation group while the greedy search identifies the pathway group from the top enriched pathways that achieves a Maximal Coverage Rate (MCR) in a sufficient number of tumor samples. In comparison to the previous approaches, MUDPAC discovers driver pathways not only by their enrichment of mutated genes but also their invariant presence among majority of samples that has been ignored previously. Using four subtypes of breast cancers as examples, our results reasserted that collaborative pathways rather than individual pathway are much more effective in discovering and interpreting the biological correlations for the complicated diseases [75, 76]. The varied distributions of mutations across the different genes in the majority of resulted driver pathways, on the other hand, also indicate the urgent needs for personalized cancer therapy.

## Methods

MUDPAC requires two input data sets: mutation data in mutation annotation format (.maf), and pathway data with topology information. The .maf file was downloaded from TCGA on March, 2013. The sample subtype information was obtained from supplementary table of [5], where PAM50 is used to stratify subtypes based on mRNA data. There are total 29,900 mutations in 498 samples after removing samples that do not fall into four subtypes. Tissue sampling is carried out by the TCGA project and no further tissue sampling is performed in this study. 269 pathways were downloaded from KEGG database [77] on Jun. of 2013, and 200 of them are used in this study after excluding 3 global metabolic pathways and 66 human disease pathways.

### Mutational pathway enrichment analysis

#### Step 1: Construct mutation matrix

Given *n* genes *G = {g*_*1*_*, g*_*2*_*, …, g*_*n*_*}*, *m* samples *S = {s*_*1*_*, s*_*2*_*, …, s*_*m*_*}*, MuSiC [12] is used to calculate the total number of bases for each gene *g*_*i*_ having available alignment data. MuSiC is a comprehensive mutational analysis pipeline that establishes correlations among mutation sites, genes and pathways. It uses Broad Institute’s analysis infrastructure Firehose to count bases with sufficient coverage of each gene from the given wiggle tract format file. Wiggle files contain dense, continuous data such as GC percent, probability scores, and transcriptome data, and were downloaded from http://gdac.broadinstitute.org on Aug 2012. The thresholds for sufficient coverage are at least 8 fold read depth in normal tissue, and at least 14 fold read depth in cancer tissue.

The functional impact score *MA*_*k*_ is calculated using MutationAssessor [78] for each somatic mutation *k* in a TCGA data set. A simple scoring rule similar to [79] is developed to assess the impacts of other types of mutations because MutationAssessor can only identify non-synonymous mutations or missense polymorphisms: the highest score that can be calculated using MutationAssessor is assigned to all indels, nonsense mutations and splice site mutations, under the assumption that these variants are at least as disruptive to protein function as the most disruptive non-synonymous mutation; the lowest score from MutationAssessor is allocated to synonymous mutations, based on the assumption that they are no more disruptive to protein function than the least disruptive non-synonymous mutations; the average score of all missense mutations is assigned to other missense mutations that can’t be calculated using MutationAssessor. The scoring scheme can be summarized below:


A matrix *M* of size 2n*m is formed with a pair of rows for each gene. The elements of alternating rows contain mutation scores based on synonymous and non-synonymous mutations, which we define *M*_*ij*__* (the wild card, * = non-synonymous/synonymous mutations (NSY/SY)) for gene *i* in sample *j* as the number of mutated bases, weighted by the functional impact score of this mutation, divided by the total number of bases that have sufficient read depth.

#### *Step 2: Calculate gene Mutation Factor (****MF)***

The mutation factor *MF*_*i*_ for gene *i* is defined as difference between the synonymous and non-synonymous mutation impacts, averaged across all samples, multiplied by a gene-specific weighting factor. The weighting factor is the ratio of the number of non-synonymous (*N*_*i_NSY*_) to synonymous (*N*_*i_SY*_) mutations for gene *i*.


#### *Step 3: Calculate gene Interaction Factor (****IF)***

The interaction factor (*IF*) for a gene *g*_*i*_, which is to be ranked, is designed to take account of the mutation landscape in its functional neighborhood [19]. In particular we incorporate the following.

(i) The shortest distance *d*_*ij*_ between *g*_*i*_ and *g*_*j*_, where *j* runs over all genes in the KEGG pathway that contains gene *i*. It takes account of the influence that a mutation in one gene has on others in terms of the functional distance between them [80] under the assumption that genes located closer to each other in a functional network tend to take less time to spread mutational information from one to the other.

(ii) *C*_*ij*_ measures the covered samples by *g*_*i*_ and *g*_*j*_, which is defined as the fraction of samples in which only one of the genes, either *g*_*i*_ or *g*_*j*_, is non-synonymously mutated.

(iii) *ME*_*ij*_ measures the mutual exclusivity of *g*_*i*_ and *g*_*j*_, which is defined as the number of samples in which only one of the genes is non-synonymously mutated, divided by the number of samples in which at least one of the genes is non-synonymously mutated [14].

We define the *IF* of a gene *i* as the average mutational influence that this gene imposes on the rest of the genes in the pathway [19]. Smaller *IF*_*i*_ means the pairwise mutational relation dominated by gene *i* exhibits a relatively neutral effect to the pathway [81]. Conversely, if *IF*_*i*_ is high, gene *i* could aggregately perturb the pathway by a strong connection to others. *IF*_*i*_ only counts genes that with interaction to gene *i* higher than a threshold of α, which is used to control the sensitivity and selectivity of the *IF* and is set as 0.005 in our following experiment.

The log of the interaction factor *IF*_*i*_ for gene *i* in pathway *k* is defined as the interaction  between gene *i* and gene *j*, averaged over all genes in the pathway.


Where


*Θ* is a function that compares if *f*_*ij*_ satisfy a pre-defined threshold α, which is used to control the contribution of *g*_*i*_ to *f*_*ij*_.

#### Step 4: Compute gene ranking list by integrating MF and IF

The ranking score for each gene is computed by combining the mutation and interaction factors for that gene.


Since *MF* can be either positive or negative and 0 < *IF* < 1, exponential function is applied to make sure the ranking score increases as *MF* and *IF* increases.

#### Step 5: Impute score for genes that are not in pathway

For a given pathway, the scores of those genes outside the pathway are imputed using information from genes inside the pathway since their topology information is not available. In practice, the imputations are performed after the scores of all genes from all pathways are computed, i.e., a background distribution is generated by parameterizing from the mean and standard deviation of all gene scores from all pathways instead of individual pathways. Scores for the genes outside a pathway are then calculated by drawing random samples from the background distribution.

Under the consideration that not all genes within a pathway can pass *Θ*, MUDPAC measures the possibility of passing event for genes inside the pathway and applies this possibility to the genes outside the pathway while performing imputation, i.e., imputation will be only carried out when a passing event happens. The aim of this is to keep the same percentage of genes inside a pathway and outside a pathway in order to maintain the distribution of their *IF* scores. Imputations of *IF* scores for genes outside a pathway are important for fair ranking to avoid artificial bias toward genes inside a pathway.

#### Step 6: Calculate statistical significance of each pathway

The statistical significance of each pathway is calculated by Weighted Kolmogorov–Smirnov (WKS) test. The cumulative distribution function (CDFs) of genes that are in a pathway *P*_*k*_ and that are not in *P*_*k*_ at position *i* in the rank can be written as:

and


where  and *N*_*Not Pk*_ is the number of all genes not belonging to *P*_*k*_. The maximum deviation (MD) of *CDF*(*P*_*k*_*,i*) and *CDF*(*Not P*_*k*_*,i*) is calculated after *n* permutations of mutation status shuffling and the *P*-value of pathway *P*_*k*_ is the percentage of iterations that have higher maximum deviations than the original data. In our experiments, *n* is set to 5000.

#### Step 7: Perform the multiple testing

After *P*-values for all pathways are computed, *FDR* is calculated to correct for multiple testing with FDR = P*m/k, where *m* is the total number of pathways and *k* is the rank of the pathway under consideration.

### Collaborative driver pathway identification

To access the biological relevance of the identified candidate driver pathways, a greedy algorithm is developed to identify sets of cooperative pathways by examining if the co-occurrence patterns of pathways are conserved in the majority of cancer samples, with the requirement that the Maximal Coverage Rate (MCR) of driver pathways is higher than the maximum mutation rate of any single genes in a given pathway. We use the Top 60 pathways identified in Step 1, and rank each by its coverage rate, i.e. by the number of samples in which it has at least one non-synonymous mutation. We then walk down the ranking list, starting with the pathway of highest coverage rate, add one more pathway in each step to achieve the MCR with existing collaborative pathways and satisfy the two criteria detailed below. The algorithm terminates when there are no pathways satisfying above constrains can be added.

Two more criteria are considered when selecting a new pathway into the collaborative set. First, the newly selected pathway along with all pathways already in collaborative pathway set, should have a MCR higher than the highest mutation rate of genes in this particular pathway by a given threshold, which is set to be 5% in this study (more discussion can be found in Additional file 1). The choice of 5% is based on the comprehensive tests to balance the sensitivity and selectivity. This parameter remains adjustable for future applications. Second, permutation is used to test whether MCR of this newly selected pathway is significantly higher than a background MCR where all mutations across all samples in this newly selected pathway are randomly redistributed across different samples while the total number of mutations remains unchanged. The background MCR is obtained by calculating the percentage of samples with at least one randomly assigned mutation in this newly selected pathway, and at least one mutation in each of previously selected pathways. The permutation is carried out 5000 times for the selection of each new pathway. The *P*-value is computed as the percentage of times when the background MCRs are greater than or equal to the observed MCR of the selected pathway. Pathway can be selected only if it satisfies a *P*-value threshold of 0.01. If the pathway with the highest MCR does not satisfy these two criteria, the pathway with second highest MCR will be examined, so on and so forth.

### Availability of supporting data

The data sets supporting the results of this article and source codes of MUDPAC are available at http://www.visantnet.org/misi/MUDPAC.zip.

## Electronic supplementary material

Additional file 1: **Supplementary methods and supplementary figures.** The .doc file contains the description of supplementary methods to explain how 5% threshold is selected in Step 2, a table of top 5 genes in all the driver pathways of each subtype, validation results with COSMIC mutated genes, validation results with cell line mutated genes, comparison with other method (DrGaP) for mutational pathway enrichment analysis. It also contains supplementary figures: KEGG 2D color plots of all driver pathways for each subtype. (DOC 1 MB)

Additional file 2: **Supplementary tables.** This .xls file contains details of selected pathway and pathway collaboration for each subtype. (XLSX 65 KB)

## Abbreviations

TCGA: The cancer genome atlas
ICGC: International cancer genome consortium
MUDPAC: Mutational driver pathway collaboration
PWEA: Pathway enrichment analysis
GSEA: Gene set enrichment analysis
MF: Mutation factor
IF: Interaction factor
BRCA: Breast cancer
MCR: Maximal coverage rate
KEGG: Kyoto encyclopedia of genes and genomes
COSMIC: Catalogue of somatic mutations in cancer
OMIM: Online mendelian inheritance in man
ER: Estrogen receptor
MEMo: Mutual exclusivity modules
MuSiC: Mutational significance in cancer
PARADIGM: Pathway recognition algorithm using data integration on genomic models
Dendrix: De novo driver exclusivity
MDPFinder: Mutated driver pathway finder
DrGaP: Driver genes and pathways
Multi-Dendrix: Multiple pathway de novo driver exclusivity
FLN: Functional linkage network
HRN: Human reference network
WKS: Weighted Kolmogorov–Smirnov
CDF: Cumulative distribution function
MD: Maximum deviation.
