# Supplementary material for: Identification of collaborative driver pathways in breast cancer
Source: BMC Genomics. 2014 Jul 17;15(1):605. doi: 10.1186/1471-2164-15-605 (PMC4111852; doi:10.1186/1471-2164-15-605)
Supplement: Supplementary file 1 — Additional file 1: Supplementary methods and supplementary figures. The .doc file contains the description of supplementary methods to explain how 5% threshold is selected in Step 2, a table of top 5 genes in all the driver pathways of each subtype, validation results with COSMIC mutated genes, validation results with cell line mutated genes, comparison with other method (DrGaP) for mutational pathway enrichment analysis. It also contains supplementary figures: KEGG 2D color plots of all driver pathways for each subtype. (DOC 1 MB) [file 12864_2014_6279_MOESM1_ESM.doc]

**Supplementary methods and supplementary figures - Identification of collaborative driver pathways in breast cancer**

**Collaborative driver pathway identification**

When implementing Step 2 of ***Mu***tational ***D***river ***Pa***thway ***C***ollaboration (MUDPAC), a straightforward greedy algorithm is introduced to find out pathway collaborations. The greedy algorithm follows locally optimal choice at each stage and can be done within limited steps, but can’t always produce an optimal solution. When applying to the real data, we usually run additional one step forward to check which one produce a better global Maximal Coverage Rate (MCR) if there are more than one pathway have the same MCR in the current step. We randomly pick one of them if the corresponding MCRs are still equal. MUDPAC terminates when either of the following criteria satisfy first: no more pathways can be selected by our algorithm; MCR of driver pathways is lower than a threshold, which is 35% in current version.

We pick Top 60 enriched pathways to find the driver pathway collaborations instead of a FDR cutoff because we try to select pathway collaborations based on the same number of candidate pathways for each subtype, and also would like to include as many candidate pathways as possible because step 2 will be able to filter out more false positives. This results in a relative loose FDR cutoff of 0.3 (0.25 is the common cutoff for enrichment analysis).

The requirement of MCR for selected pathway groups is 5% higher than single gene mutation rate. The choice of 5% is based on experimental results to achieve balance between sensitivity and selectivity. We tried 1%, 3%, 5%, 7%, 9%, 10%, detailed results can be seen in Additional file 2. For the three subtypes of Basal-like, Luminal-A and Luminal-B, the bigger threshold will always results in less driver pathways selected, as shown in Figure S1. For HER2+, the number of driver pathways reach its maximum when threshold equal to 5%, and decrease after that. We found that thresholds of 1% and 3% can’t be very helpful to screen pathways, and thresholds of 7%, 9%, 10% are too sensitive to exclude lots of them. Threshold of 5% works most efficiently in all these 4 subtypes with some subtype-specific pathways selected, like “Estrogen signaling” pathway only in Luminal-A and Luminal-B, “ErbB signaling” in HER2+ and Luminal-B.


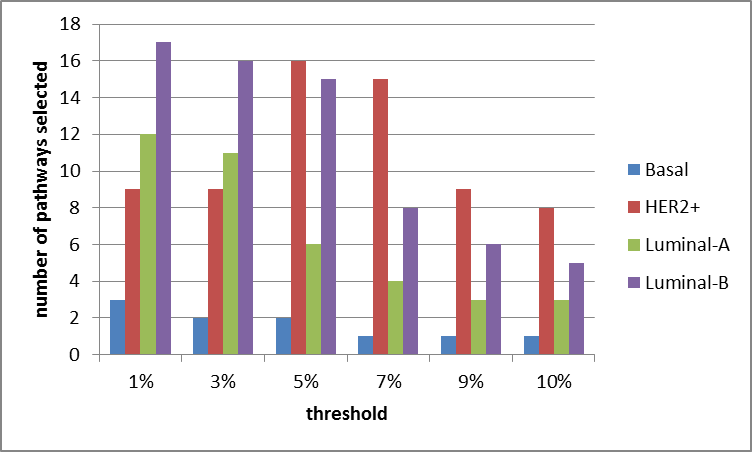


**Figure S1. Number of driver pathways selected for each subtype when applying different thresholds requirement for MCR to be higher than single gene mutation rate.**

Table S1. Top 5 genes in driver pathways of each subtype. For each driver pathway in each subtype, the top 5 genes with highest ranking score are listed. If this pathway is not a driver in a subtype, its corresponding value is left blank.

|  | **Basal-like** | **HER2+** | **Luminal-A** | **Luminal-B** |
| --- | --- | --- | --- | --- |
| PI3K-Akt signaling | TP53, LAMA5, ITGAV, PRKAA2, LAMA1 | TP53, ITGAV, PTEN, TLR4, COL5A3 | PTEN, PIK3CA, CDKN1B, KIT, TP53 | PTEN, TP53, TLR4, PIK3CA, KIT |
| MAPK signaling | TP53, NF1, NFKB2, CACNA1B, RPS6KA6 | TP53, DUSP4, PLA2G4F, MAP3K5, CRKL |  |  |
| Apoptosis |  | TP53, CAPN2, PIK3R1, PIK3CA, CSF2RB |  | TP53, PIK3CA, IRAK1, ATM, PIK3R1 |
| Phosphatidylinositol signaling system |  | PTEN, ITPR3, PIK3R1, PIK3CA, PLCB2 |  | PTEN, PIK3CA, ITPR3, PLCE1, ITPKC |
| Regulation of actin cytoskeleton |  | ITGAV, ARPC2, PIK3R1, MYLK, PIK3CA | PIK3CA, ITGA6, ITGAV, ARPC2, PIK3R1 |  |
| Focal adhesion |  | CAPN2, ITGAV, PTEN, ILK, COL5A3 | PTEN, PIK3CA, COL5A3, ITGA6, ITGAV | PTEN, PIK3CA, LAMA5, VWF, COL6A3 |
| T cell receptor signaling |  | PTPRC, PIK3R1, PIK3CA, NFAT5, PIK3CG |  | PIK3CA, ZAP70, NFATC4, PAK7, PIK3R1 |
| Cholinergic synapse |  | ITPR3, PIK3R1, GNAO1, PIK3CA, KCNJ2 |  |  |
| ErbB signaling |  | ERBB3, CDKN1B, PIK3R1, PIK3CA, ABL2 |  | PIK3CA, CAMK2D, CDKN1B, TGFA, MAP2K4 |
| Chemokine signaling |  | PIK3R1, PIK3CA, CCL21, CRKL, PLCB2 | PIK3CA, PIK3R1, CCR10, ADCY7, AKT1 |  |
| Insulin signaling |  | PIK3R1, PIK3CA, CRKL, TRIP10, RAPGEF1 |  | PIK3CA, ACACA, PPARGC1A, PHKA1, TRIP10 |
| TNF signaling |  | PIK3R1, PIK3CA, MAP3K5, PIK3CG, MAP2K4 |  |  |
| Osteoclast differentiation |  | PIK3R1, PIK3CA, PIK3CG, IRF9, CYBB |  |  |
| Aldosterone-regulated sodium reabsorption |  | NEDD4L, PIK3R1, PIK3CA, SGK1, ATP1A4 |  |  |
| Carbohydrate digestion and absorption |  | PIK3R1, PIK3CA, PLCB2, SI, ATP1A4 |  | PIK3CA, PLCB2, PIK3R1, LCT, AKT1 |
| Natural killer cell mediated cytotoxicity |  | PIK3R1, PIK3CA, PTPN11, NFAT5, PIK3CG |  | PIK3CA, HLA-G, ZAP70, NFATC4, PIK3R1 |
| Jak-STAT signaling |  |  | PIK3CA, LIFR, PIK3R1, IL2RG, IL10RA | PIK3CA, LIFR, PIK3R1, CBLC, EP300 |
| Estrogen signaling |  |  | PIK3CA, PIK3R1, ADCY7, AKT1, HSP90AB1 | PIK3CA, ITPR3, GABBR1, ADCY7, NOS3 |
| HIF-1 signaling |  |  |  | TLR4, PIK3CA, CAMK2D, CDKN1B, PDHA1 |
| mTOR signaling |  |  |  | PTEN, PIK3CA, RPS6KA6, RPS6KA2, PIK3R1 |
| Progesterone-mediated oocyte maturation |  |  |  | PIK3CA, RPS6KA6, ADCY7, RPS6KA2, CDC25C |
| Cholinergic synapse |  |  |  | PIK3CA, ITPR3, CAMK2D, CHRNA6, ADCY7 |

**Validation with COSMIC mutated genes**

COSMIC public breast cancer data set was chosen to validate the pathways selected by our method in breast cancer. The number of mutated genes in basal-like, HER2+, Luminal-A and Luminal-B are 2179, 848, 1 and 2 respectively (April, 2014), since number of mutated genes are too small to do any tests in both Luminal-A and Luminal-B, we ignored the subtype information and did the following analysis based on all samples.

All mutated genes from the 4 subtypes were collected no matter what its mutation frequency is. There are 2767 unique mutated genes and they were all uploaded into The Database for Annotation, Visualization and Integrated Discovery (DAVID) to have a pathway enrichment analysis. Significantly mutated KEGG pathways (*P*<0.1) as well as an overlapping status with our pathway enrichment analysis (Step 1) are shown in Table S2. Among 36 pathways reported by DAVID, 34 of them are also reported by our method in at least one of the subtypes, which is an independent confirmatory.

**Table S2. Enriched KEGG pathways in COSMIC breast cancer mutational genes (*P*<0.1).** All mutated genes irrelevant in which subtype they are mutated are uploaded into DAVID to do KEGG pathway enrichment analysis, columns are KEGG pathway name, number of genes mutated in this pathway along with percentage, *P*-value, *FDR*, whether this pathway is selected in our pathway enrichment step and in which subtype it is selected.

| **Pathway** | **NO of genes**  **(percentage)** | **P** | **FDR** | **Selected by our method (subtypes)** |
| --- | --- | --- | --- | --- |
| [Focal adhesion](http://david.abcc.ncifcrf.gov/kegg.jsp?path=hsa04510$Focal adhesion&termId=470038853&source=kegg) | 66 (2.5) | 7.7E-9 | 7.3E-7 | Y (basal, HER2+, Luminal-A, Luminal-B) |
| [ErbB signaling pathway](http://david.abcc.ncifcrf.gov/kegg.jsp?path=hsa04012$ErbB signaling pathway&termId=470038828&source=kegg) | 33 (1.2) | 2.6E-6 | 1.2E-4 | Y (basal, HER2+, Luminal-A, Luminal-B) |
| [MAPK signaling pathway](http://david.abcc.ncifcrf.gov/kegg.jsp?path=hsa04010$MAPK signaling pathway&termId=470038827&source=kegg) | 71 (2.6) | 1.5E-5 | 2.6E-4 | Y (basal, HER2+, Luminal-A, Luminal-B) |
| [ECM-receptor interaction](http://david.abcc.ncifcrf.gov/kegg.jsp?path=hsa04512$ECM-receptor interaction&termId=470038854&source=kegg) | 30 (1.1) | 3.1E-5 | 4.9E-4 | Y (basal, HER2+, Luminal-A, Luminal-B) |
| [Dorso-ventral axis formation](http://david.abcc.ncifcrf.gov/kegg.jsp?path=hsa04320$Dorso-ventral axis formation&termId=470038847&source=kegg) | 13 (0.5) | 2.1E-4 | 2.7E-3 | Y (Luminal-A) |
| [Axon guidance](http://david.abcc.ncifcrf.gov/kegg.jsp?path=hsa04360$Axon guidance&termId=470038851&source=kegg) | 38 (1.4) | 2.5E-4 | 2.9E-3 | Y (basal, HER2+, Luminal-A, Luminal-B) |
| [Neurotrophin signaling pathway](http://david.abcc.ncifcrf.gov/kegg.jsp?path=hsa04722$Neurotrophin signaling pathway&termId=470038877&source=kegg) | 36 (1.3) | 5.0E-4 | 5.0E-3 | Y (HER2+, Luminal-B) |
| [B cell receptor signaling pathway](http://david.abcc.ncifcrf.gov/kegg.jsp?path=hsa04662$B cell receptor signaling pathway&termId=470038870&source=kegg) | 25 (0.9) | 5.4E-4 | 5.1E-3 | Y (HER2+, Luminal-B) |
| [Gap junction](http://david.abcc.ncifcrf.gov/kegg.jsp?path=hsa04540$Gap junction&termId=470038858&source=kegg) | 28 (1.0) | 6.4E-4 | 5.8E-3 | Y (Luminal-A,  Luminal-B) |
| [Cell cycle](http://david.abcc.ncifcrf.gov/kegg.jsp?path=hsa04110$Cell cycle&termId=470038834&source=kegg) | 35 (1.3) | 1.2E-3 | 1.1E-2 | Y (basal, HER2+, Luminal-A, Luminal-B) |
| [GnRH signaling pathway](http://david.abcc.ncifcrf.gov/kegg.jsp?path=hsa04912$GnRH signaling pathway&termId=470038883&source=kegg) | 29 (1.1) | 1.5E-3 | 1.2E-2 | Y (basal, HER2+, Luminal-A, Luminal-B) |
| [T cell receptor signaling pathway](http://david.abcc.ncifcrf.gov/kegg.jsp?path=hsa04660$T cell receptor signaling pathway&termId=470038869&source=kegg) | 31 (1.2) | 1.6E-3 | 1.2E-2 | Y (HER2+, Luminal-A, Luminal-B) |
| [Notch signaling pathway](http://david.abcc.ncifcrf.gov/kegg.jsp?path=hsa04330$Notch signaling pathway&termId=470038848&source=kegg) | 17 (0.6) | 2.2E-3 | 1.5E-2 | N |
| [Fc epsilon RI signaling pathway](http://david.abcc.ncifcrf.gov/kegg.jsp?path=hsa04664$Fc epsilon RI signaling pathway&termId=470038871&source=kegg) | 24 (0.9) | 2.4E-3 | 1.6E-2 | Y (basal) |
| [ABC transporters](http://david.abcc.ncifcrf.gov/kegg.jsp?path=hsa02010$ABC transporters&termId=470038812&source=kegg) | 16 (0.6) | 2.9E-3 | 1.8E-2 | Y (basal, HER2+, Luminal-A) |
| [Vascular smooth muscle contraction](http://david.abcc.ncifcrf.gov/kegg.jsp?path=hsa04270$Vascular smooth muscle contraction&termId=470038845&source=kegg) | 31 (1.2) | 3.0E-3 | 1.8E-2 | Y (basal, Luminal-A, Luminal-B) |
| [Aldosterone-regulated sodium reabsorption](http://david.abcc.ncifcrf.gov/kegg.jsp?path=hsa04960$Aldosterone-regulated sodium reabsorption&termId=470038890&source=kegg) | 15 (0.6) | 3.9E-3 | 2.3E-2 | Y (HER2+, Luminal-B) |
| [Progesterone-mediated oocyte maturation](http://david.abcc.ncifcrf.gov/kegg.jsp?path=hsa04914$Progesterone-mediated oocyte maturation&termId=470038884&source=kegg) | 25 (0.9) | 4.3E-3 | 2.5E-2 | Y (basal, Luminal-B) |
| [Long-term potentiation](http://david.abcc.ncifcrf.gov/kegg.jsp?path=hsa04720$Long-term potentiation&termId=470038876&source=kegg) | 21 (0.8) | 4.6E-3 | 2.6E-2 | Y (Luminal-A,  Luminal-B) |
| [Toll-like receptor signaling pathway](http://david.abcc.ncifcrf.gov/kegg.jsp?path=hsa04620$Toll-like receptor signaling pathway&termId=470038862&source=kegg) | 28 (1.0) | 4.9E-3 | 2.6E-2 | Y (basal, Luminal-A, Luminal-B) |
| [Apoptosis](http://david.abcc.ncifcrf.gov/kegg.jsp?path=hsa04210$Apoptosis&termId=470038843&source=kegg) | 25 (0.9) | 5.1E-3 | 2.7E-2 | Y (HER2+, Luminal-A, Luminal-B) |
| [Long-term depression](http://david.abcc.ncifcrf.gov/kegg.jsp?path=hsa04730$Long-term depression&termId=470038878&source=kegg) | 21 (0.8) | 5.6E-3 | 2.8E-2 | Y (basal, Luminal-A, Luminal-B) |
| [Insulin signaling pathway](http://david.abcc.ncifcrf.gov/kegg.jsp?path=hsa04910$Insulin signaling pathway&termId=470038882&source=kegg) | 34 (1.3) | 8.8E-3 | 4.4E-2 | Y (basal, HER2+, Luminal-A, Luminal-B) |
| [Chemokine signaling pathway](http://david.abcc.ncifcrf.gov/kegg.jsp?path=hsa04062$Chemokine signaling pathway&termId=470038831&source=kegg) | 44 (1.6) | 9.7E-3 | 4.7E-2 | Y (HER2+, Luminal-A, Luminal-B) |
| [Phosphatidylinositol signaling system](http://david.abcc.ncifcrf.gov/kegg.jsp?path=hsa04070$Phosphatidylinositol signaling system&termId=470038832&source=kegg) | 21 (0.8) | 1.3E-2 | 5.9E-2 | Y (basal, HER2+, Luminal-A, Luminal-B) |
| [mTOR signaling pathway](http://david.abcc.ncifcrf.gov/kegg.jsp?path=hsa04150$mTOR signaling pathway&termId=470038842&source=kegg) | 16 (0.6) | 1.6E-2 | 7.1E-2 | Y (Luminal-A,  Luminal-B) |
| [Regulation of actin cytoskeleton](http://david.abcc.ncifcrf.gov/kegg.jsp?path=hsa04810$Regulation of actin cytoskeleton&termId=470038881&source=kegg) | 48 (1.8) | 1.8E-2 | 7.7E-2 | Y (basal, HER2+, Luminal-A) |
| [Tight junction](http://david.abcc.ncifcrf.gov/kegg.jsp?path=hsa04530$Tight junction&termId=470038857&source=kegg) | 32 (1.2) | 2.4E-2 | 9.9E-2 | Y (basal, HER2+, Luminal-A, Luminal-B) |
| [Hedgehog signaling pathway](http://david.abcc.ncifcrf.gov/kegg.jsp?path=hsa04340$Hedgehog signaling pathway&termId=470038849&source=kegg) | 16 (0.6) | 3.1E-2 | 1.2E-1 | Y (HER2+, Luminal-A) |
| [Natural killer cell mediated cytotoxicity](http://david.abcc.ncifcrf.gov/kegg.jsp?path=hsa04650$Natural killer cell mediated cytotoxicity&termId=470038868&source=kegg) | 31 (1.2) | 3.5E-2 | 1.4E-1 | Y (basal, HER2+, Luminal-A, Luminal-B) |
| [Wnt signaling pathway](http://david.abcc.ncifcrf.gov/kegg.jsp?path=hsa04310$Wnt signaling pathway&termId=470038846&source=kegg) | 34 (1.3) | 4.3E-2 | 1.6E-1 | Y (basal, HER2+, Luminal-A, Luminal-B) |
| [Fc gamma R-mediated phagocytosis](http://david.abcc.ncifcrf.gov/kegg.jsp?path=hsa04666$Fc gamma R-mediated phagocytosis&termId=470038872&source=kegg) | 23 (0.9) | 5.0E-2 | 1.8E-1 | Y (basal, HER2+, Luminal-A) |
| [Purine metabolism](http://david.abcc.ncifcrf.gov/kegg.jsp?path=hsa00230$Purine metabolism&termId=470038742&source=kegg) | 33 (1.2) | 7.6E-2 | 2.6E-1 | Y (basal, HER2+, Luminal-A, Luminal-B) |
| [Oocyte meiosis](http://david.abcc.ncifcrf.gov/kegg.jsp?path=hsa04114$Oocyte meiosis&termId=470038835&source=kegg) | 25 (0.9) | 7.7E-2 | 2.5E-1 | Y (basal, Luminal-A, Luminal-B) |
| [Calcium signaling pathway](http://david.abcc.ncifcrf.gov/kegg.jsp?path=hsa04020$Calcium signaling pathway&termId=470038829&source=kegg) | 37 (1.4) | 8.2E-2 | 2.6E-1 | Y (basal, HER2+, Luminal-A, Luminal-B) |
| [VEGF signaling pathway](http://david.abcc.ncifcrf.gov/kegg.jsp?path=hsa04370$VEGF signaling pathway&termId=470038852&source=kegg) | 18 (0.7) | 9.3E-2 | 2.9E-1 | N |

**Validation with cell line mutated gene**

In order to have a comparative analysis between driver pathways identified in TCGA tumor data and cancer cell line data, we downloaded breast cancer cell line data from Cancer Cell Line Encyclopedia (CCLE) . There are 51 breast cancer cell lines available on March, 2014. We extracted all mutated genes from all cell lines and applied these 1103 unique genes to DAVID to have a pathway enrichment analysis. All the enriched pathways (*P*<0.1) in KEGG reported by DAVID are summarized in Table S3. Among total 45 significantly enriched pathways reported by DAVID, 43 pathways are also reported by our method in at least one of the subtypes.

**Table S3. Enriched KEGG pathways in CCLE cell line breast cancer data (*P*<0.1).** All 1103 unique mutated genes from 51 breast cancer cell line data are uploaded into DAVID to do KEGG pathway enrichment analysis, columns are KEGG pathway name, number of genes mutated in this pathway along with percentage, *P*-value, FDR, whether this pathway is selected in our pathway enrichment step and in which subtype it is selected.

| **Pathway** | **NO of genes**  **(percentage)** | **P** | **FDR** | **Selected by our method (subtypes)** |
| --- | --- | --- | --- | --- |
| [MAPK signaling pathway](http://david.abcc.ncifcrf.gov/kegg.jsp?path=hsa04010$MAPK signaling pathway&termId=470038827&source=kegg) | 106 (9.6) | 2.8E-37 | 2.1E-35 | Y (basal, HER2+, Luminal-A, Luminal-B) |
| [Neurotrophin signaling pathway](http://david.abcc.ncifcrf.gov/kegg.jsp?path=hsa04722$Neurotrophin signaling pathway&termId=470038877&source=kegg) | 57 (5.2) | 1.9E-23 | 9.3E-22 | Y (HER2+, Luminal-B) |
| [ErbB signaling pathway](http://david.abcc.ncifcrf.gov/kegg.jsp?path=hsa04012$ErbB signaling pathway&termId=470038828&source=kegg) | 46 (4.2) | 4.8E-22 | 1.4E-20 | Y (HER2+, Luminal-A, Luminal-B) |
| [Focal adhesion](http://david.abcc.ncifcrf.gov/kegg.jsp?path=hsa04510$Focal adhesion&termId=470038853&source=kegg) | 72 (6.5) | 7.3E-22 | 1.8E-20 | Y (basal, HER2+, Luminal-A, Luminal-B) |
| [T cell receptor signaling pathway](http://david.abcc.ncifcrf.gov/kegg.jsp?path=hsa04660$T cell receptor signaling pathway&termId=470038869&source=kegg) | 48 (4.4) | 5.2E-19 | 9.7E-18 | Y (HER2+, Luminal-A, Luminal-B) |
| [mTOR signaling pathway](http://david.abcc.ncifcrf.gov/kegg.jsp?path=hsa04150$mTOR signaling pathway&termId=470038842&source=kegg) | 30 (2.7) | 1.0E-15 | 1.1E-14 | Y (Luminal-A,  Luminal-B) |
| [Apoptosis](http://david.abcc.ncifcrf.gov/kegg.jsp?path=hsa04210$Apoptosis&termId=470038843&source=kegg) | 39 (3.5) | 1.4E-15 | 1.4E-14 | Y (HER2+, Luminal-A, Luminal-B) |
| [VEGF signaling pathway](http://david.abcc.ncifcrf.gov/kegg.jsp?path=hsa04370$VEGF signaling pathway&termId=470038852&source=kegg) | 33 (3.0) | 5.9E-13 | 4.8E-12 | N |
| [B cell receptor signaling pathway](http://david.abcc.ncifcrf.gov/kegg.jsp?path=hsa04662$B cell receptor signaling pathway&termId=470038870&source=kegg) | 33 (3.0) | 5.9E-13 | 4.8E-12 | Y (HER2+, Luminal-B) |
| [GnRH signaling pathway](http://david.abcc.ncifcrf.gov/kegg.jsp?path=hsa04912$GnRH signaling pathway&termId=470038883&source=kegg) | 38 (3.5) | 8.3E-13 | 6.2E-12 | Y (basal, HER2+, Luminal-A, Luminal-B) |
| [Insulin signaling pathway](http://david.abcc.ncifcrf.gov/kegg.jsp?path=hsa04910$Insulin signaling pathway&termId=470038882&source=kegg) | 45 (4.1) | 2.1E-12 | 1.5E-11 | Y (basal, HER2+, Luminal-A, Luminal-B) |
| [Regulation of actin cytoskeleton](http://david.abcc.ncifcrf.gov/kegg.jsp?path=hsa04810$Regulation of actin cytoskeleton&termId=470038881&source=kegg) | 59 (5.4) | 5.0E-12 | 3.4E-11 | Y (basal, HER2+, Luminal-A) |
| [Toll-like receptor signaling pathway](http://david.abcc.ncifcrf.gov/kegg.jsp?path=hsa04620$Toll-like receptor signaling pathway&termId=470038862&source=kegg) | 37 (3.4) | 1.2E-11 | 8.0E-11 | Y (basal, Luminal-A, Luminal-B) |
| [Progesterone-mediated oocyte maturation](http://david.abcc.ncifcrf.gov/kegg.jsp?path=hsa04914$Progesterone-mediated oocyte maturation&termId=470038884&source=kegg) | 32 (2.9) | 2.5E-10 | 1.4E-9 | Y (basal, Luminal-B) |
| [Fc epsilon RI signaling pathway](http://david.abcc.ncifcrf.gov/kegg.jsp?path=hsa04664$Fc epsilon RI signaling pathway&termId=470038871&source=kegg) | 30 (2.7) | 4.1E-10 | 2.3E-9 | Y (basal) |
| [Axon guidance](http://david.abcc.ncifcrf.gov/kegg.jsp?path=hsa04360$Axon guidance&termId=470038851&source=kegg) | 39 (3.5) | 2.0E-9 | 1.0E-8 | Y (basal, HER2+, Luminal-A, Luminal-B) |
| [Gap junction](http://david.abcc.ncifcrf.gov/kegg.jsp?path=hsa04540$Gap junction&termId=470038858&source=kegg) | 30 (2.7) | 1.4E-8 | 6.7E-8 | Y (Luminal-A,  Luminal-B) |
| [TGF-beta signaling pathway](http://david.abcc.ncifcrf.gov/kegg.jsp?path=hsa04350$TGF-beta signaling pathway&termId=470038850&source=kegg) | 29 (2.6) | 3.4E-8 | 1.6E-7 | Y (basal, Luminal-A) |
| [Chemokine signaling pathway](http://david.abcc.ncifcrf.gov/kegg.jsp?path=hsa04062$Chemokine signaling pathway&termId=470038831&source=kegg) | 46 (4.2) | 6.7E-8 | 3.0E-7 | Y (HER2+, Luminal-A, Luminal-B) |
| [Phosphatidylinositol signaling system](http://david.abcc.ncifcrf.gov/kegg.jsp?path=hsa04070$Phosphatidylinositol signaling system&termId=470038832&source=kegg) | 25 (2.3) | 2.8E-7 | 1.2E-6 | Y (basal, HER2+, Luminal-A, Luminal-B) |
| [Vascular smooth muscle contraction](http://david.abcc.ncifcrf.gov/kegg.jsp?path=hsa04270$Vascular smooth muscle contraction&termId=470038845&source=kegg) | 32 (2.9) | 3.0E-7 | 1.3E-6 | Y (basal, Luminal-A, Luminal-B) |
| [Wnt signaling pathway](http://david.abcc.ncifcrf.gov/kegg.jsp?path=hsa04310$Wnt signaling pathway&termId=470038846&source=kegg) | 38 (3.5) | 6.4E-7 | 2.6E-6 | Y (basal, HER2+, Luminal-A, Luminal-B) |
| [Cell cycle](http://david.abcc.ncifcrf.gov/kegg.jsp?path=hsa04110$Cell cycle&termId=470038834&source=kegg) | 33 (3.0) | 1.3E-6 | 5.4E-6 | Y (basal, HER2+, Luminal-A, Luminal-B) |
| [Natural killer cell mediated cytotoxicity](http://david.abcc.ncifcrf.gov/kegg.jsp?path=hsa04650$Natural killer cell mediated cytotoxicity&termId=470038868&source=kegg) | 34 (3.1) | 1.9E-6 | 7.6E-6 | Y (basal, HER2+, Luminal-A, Luminal-B) |
| [Adherens junction](http://david.abcc.ncifcrf.gov/kegg.jsp?path=hsa04520$Adherens junction&termId=470038856&source=kegg) | 24 (2.2) | 2.5E-6 | 9.6E-6 | Y (basal, HER2+, Luminal-A, Luminal-B) |
| [Notch signaling pathway](http://david.abcc.ncifcrf.gov/kegg.jsp?path=hsa04330$Notch signaling pathway&termId=470038848&source=kegg) | 18 (1.6) | 2.9E-6 | 1.1E-5 | N |
| [Adipocytokine signaling pathway](http://david.abcc.ncifcrf.gov/kegg.jsp?path=hsa04920$Adipocytokine signaling pathway&termId=470038886&source=kegg) | 22 (2.0) | 2.9E-6 | 1.0E-5 | Y (Luminal-A) |
| [Fc gamma R-mediated phagocytosis](http://david.abcc.ncifcrf.gov/kegg.jsp?path=hsa04666$Fc gamma R-mediated phagocytosis&termId=470038872&source=kegg) | 27 (2.5) | 3.5E-6 | 1.2E-5 | Y (basal, HER2+, Luminal-A) |
| [NOD-like receptor signaling pathway](http://david.abcc.ncifcrf.gov/kegg.jsp?path=hsa04621$NOD-like receptor signaling pathway&termId=470038863&source=kegg) | 19 (1.7) | 4.8E-5 | 1.6E-4 | Y (Luminal-A) |
| [Oocyte meiosis](http://david.abcc.ncifcrf.gov/kegg.jsp?path=hsa04114$Oocyte meiosis&termId=470038835&source=kegg) | 27 (2.5) | 5.9E-5 | 1.9E-4 | Y (basal, Luminal-A, Luminal-B) |
| [Melanogenesis](http://david.abcc.ncifcrf.gov/kegg.jsp?path=hsa04916$Melanogenesis&termId=470038885&source=kegg) | 25 (2.3) | 7.4E-5 | 2.3E-4 | Y (basal, HER2+, Luminal-A, Luminal-B) |
| [Endocytosis](http://david.abcc.ncifcrf.gov/kegg.jsp?path=hsa04144$Endocytosis&termId=470038841&source=kegg) | 38 (3.5) | 8.0E-5 | 2.4E-4 | Y (basal, HER2+, Luminal-A, Luminal-B) |
| [Dorso-ventral axis formation](http://david.abcc.ncifcrf.gov/kegg.jsp?path=hsa04320$Dorso-ventral axis formation&termId=470038847&source=kegg) | 11 (1.0) | 1.3E-4 | 3.8E-4 | Y (Luminal-A) |
| [Calcium signaling pathway](http://david.abcc.ncifcrf.gov/kegg.jsp?path=hsa04020$Calcium signaling pathway&termId=470038829&source=kegg) | 36 (3.3) | 1.6E-4 | 4.6E-4 | Y (basal, HER2+, Luminal-A, Luminal-B) |
| [Long-term depression](http://david.abcc.ncifcrf.gov/kegg.jsp?path=hsa04730$Long-term depression&termId=470038878&source=kegg) | 19 (1.7) | 2.2E-4 | 6.3E-4 | Y (basal, Luminal-A, Luminal-B) |
| [Inositol phosphate metabolism](http://david.abcc.ncifcrf.gov/kegg.jsp?path=hsa00562$Inositol phosphate metabolism&termId=470038776&source=kegg) | 16 (1.5) | 3.5E-4 | 9.8E-4 | Y (basal, Luminal-A, Luminal-B) |
| [p53 signaling pathway](http://david.abcc.ncifcrf.gov/kegg.jsp?path=hsa04115$p53 signaling pathway&termId=470038836&source=kegg) | 18 (1.6) | 5.6E-4 | 1.5E-3 | Y (basal, Luminal-A, Luminal-B) |
| [RIG-I-like receptor signaling pathway](http://david.abcc.ncifcrf.gov/kegg.jsp?path=hsa04622$RIG-I-like receptor signaling pathway&termId=470038864&source=kegg) | 18 (1.6) | 9.5E-4 | 2.6E-3 | Y (Luminal-A) |
| [Cytokine-cytokine receptor interaction](http://david.abcc.ncifcrf.gov/kegg.jsp?path=hsa04060$Cytokine-cytokine receptor interaction&termId=470038830&source=kegg) | 45 (4.1) | 1.2E-3 | 3.2E-3 | Y (basal, Luminal-A, Luminal-B) |
| [Jak-STAT signaling pathway](http://david.abcc.ncifcrf.gov/kegg.jsp?path=hsa04630$Jak-STAT signaling pathway&termId=470038866&source=kegg) | 30 (2.7) | 1.6E-3 | 4.1E-3 | Y (basal, Luminal-A, Luminal-B) |
| [Hedgehog signaling pathway](http://david.abcc.ncifcrf.gov/kegg.jsp?path=hsa04340$Hedgehog signaling pathway&termId=470038849&source=kegg) | 15 (1.4) | 1.7E-3 | 4.3E-3 | Y (HER2+, Luminal-A) |
| [Aldosterone-regulated sodium reabsorption](http://david.abcc.ncifcrf.gov/kegg.jsp?path=hsa04960$Aldosterone-regulated sodium reabsorption&termId=470038890&source=kegg) | 12 (1.1) | 2.9E-3 | 7.2E-3 | Y (HER2+, Luminal-B) |
| [Tight junction](http://david.abcc.ncifcrf.gov/kegg.jsp?path=hsa04530$Tight junction&termId=470038857&source=kegg) | 25 (2.3) | 6.9E-3 | 1.6E-2 | Y (basal, HER2+, Luminal-A, Luminal-B) |
| [ECM-receptor interaction](http://david.abcc.ncifcrf.gov/kegg.jsp?path=hsa04512$ECM-receptor interaction&termId=470038854&source=kegg) | 16 (1.5) | 3.0E-2 | 6.8E-2 | Y (basal, HER2+, Luminal-A, Luminal-B) |
| [Homologous recombination](http://david.abcc.ncifcrf.gov/kegg.jsp?path=hsa03440$Homologous recombination&termId=470038825&source=kegg) | 7 (0.6) | 7.0E-2 | 1.5E-1 | Y (Luminal-A) |

**Comparison with other method for mutational pathway enrichment analysis**

In order to validate the mutational candidate driver pathways identified in our first step, we compared our results with another mutational pathway identification method: DrGaP (Driver genes and pathways) . DrGaP is a computational tool that integrates biological knowledge of the mutational process in tumors into statistical models. Compared with regular statistical models, they propose a heuristic strategy to estimate the mixture proportion of chi-square distribution of likelihood ratio test (LRT) statistics, which significantly increase statistical power to detect low prevalent of somatic mutations. For the biological knowledge, they include variables such as the length of protein-coding regions, transcript isoforms, variation in mutation types, differences in background mutation rates, the redundancy of genetic code, and multiple mutations in one gene.

The same breast cancer data from TCGA and the same KEGG pathways were used: .maf file downloaded on March 2013 with 29900 somatic mutations and 498 samples distributed over 4 subtypes, 200 KEGG pathways after excluded 3 global metabolic and 66 human disease pathways, downloaded from KEGG (http://www.genome.jp/kegg/pathway.html) on Jun 2013.

The 4 subtypes of basal-like, HER2+, Luminal-A, Luminal-B were analyzed and compared separately. The following 4 venny diagrams show the comparisons between DrGaP and our method under *P*<0.1. We can see that in all the 4 circumstances, MUDPAC is always sensitive to discover more mutational enriched pathways, and in addition, the pathways identified are more related to cancer or to that specific subtype. For example, besides the well-known cancer related pathways like “Focal adhesion”, “PI3K-Akt signaling”, “Wnt signaling”, “MAPK signaling”, “Cell cycle”, we also could find some pathways that are representative in that particular subtype, like “p53 signaling” in basal-like, “ErbB signaling” and “Insulin signaling” in HER2+, “Estrogen signaling” in both Luminal-A and Luminal-B.


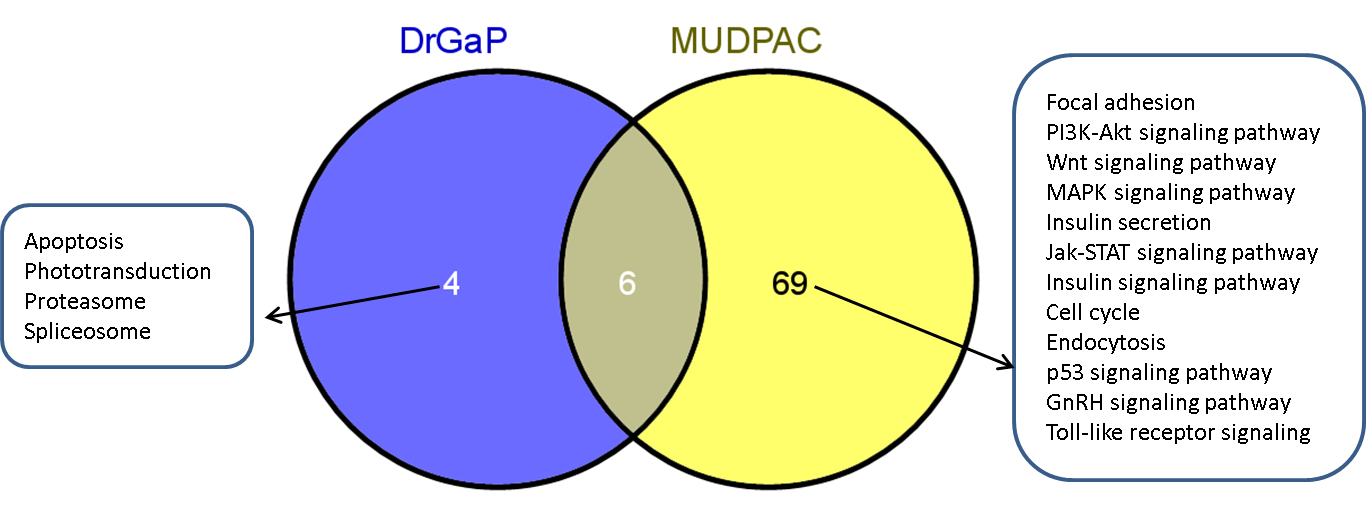


**Figure S2. Comparison of mutational enriched pathways between DrGaP and MUDPAC in basal-like subtype (*P*<0.1).**


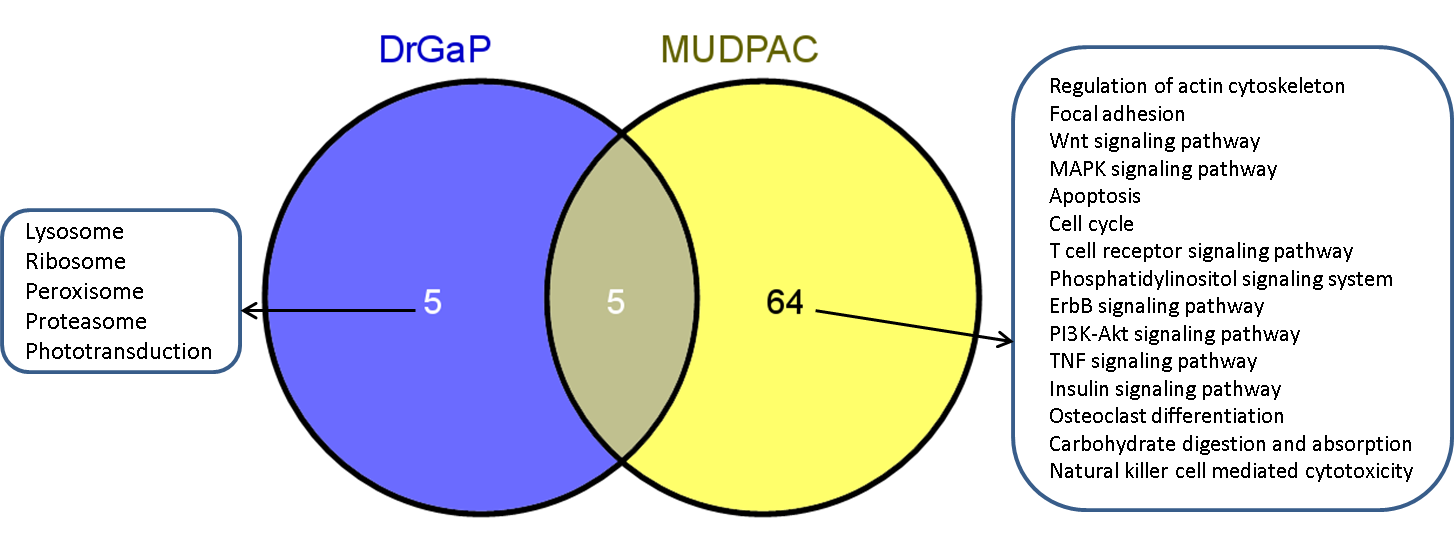


**Figure S3. Comparison of mutational enriched pathways between DrGaP and MUDPAC in HER2+ subtype (*P*<0.1).**


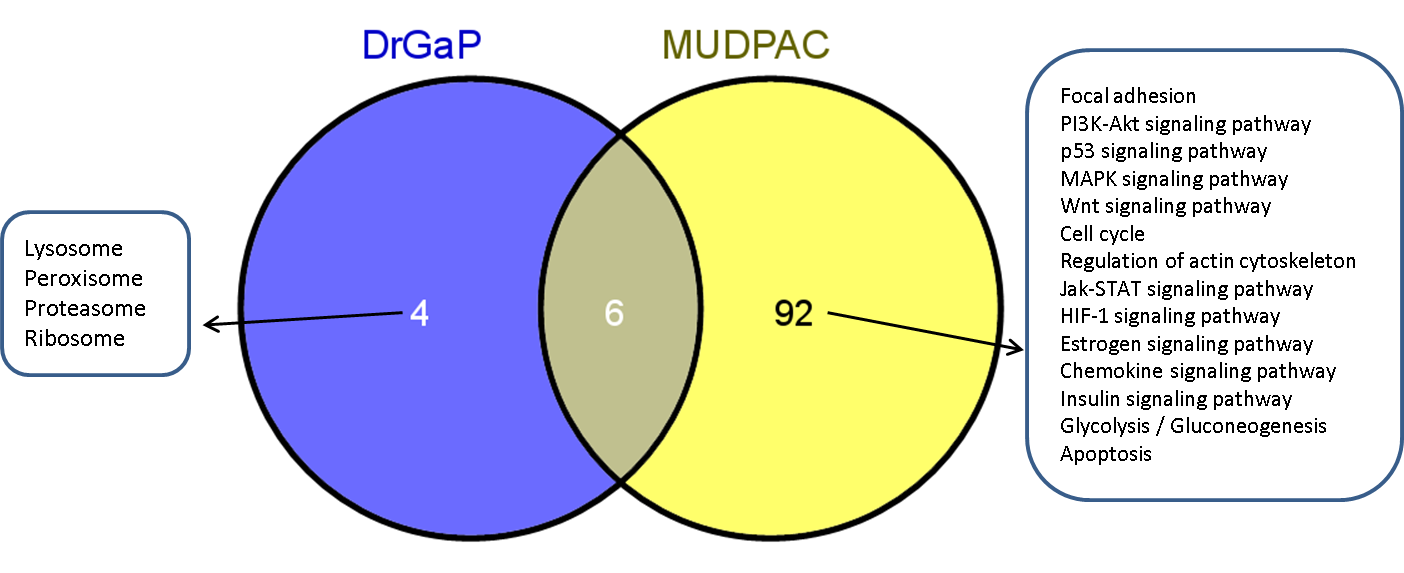


**Figure S4. Comparison of mutational enriched pathways between DrGaP and MUDPAC in Luminal-A subtype (*P*<0.1).**


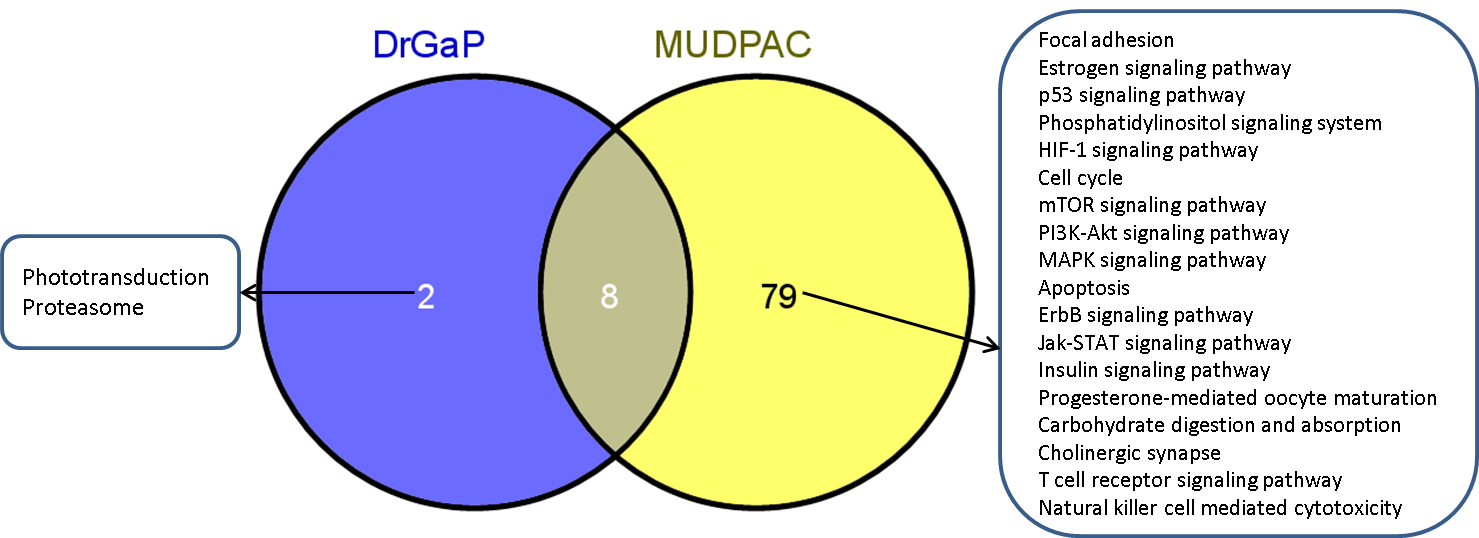


**Figure S5. Comparison of mutational enriched pathways between DrGaP and MUDPAC in Luminal-B subtype (*P*<0.1).**

**
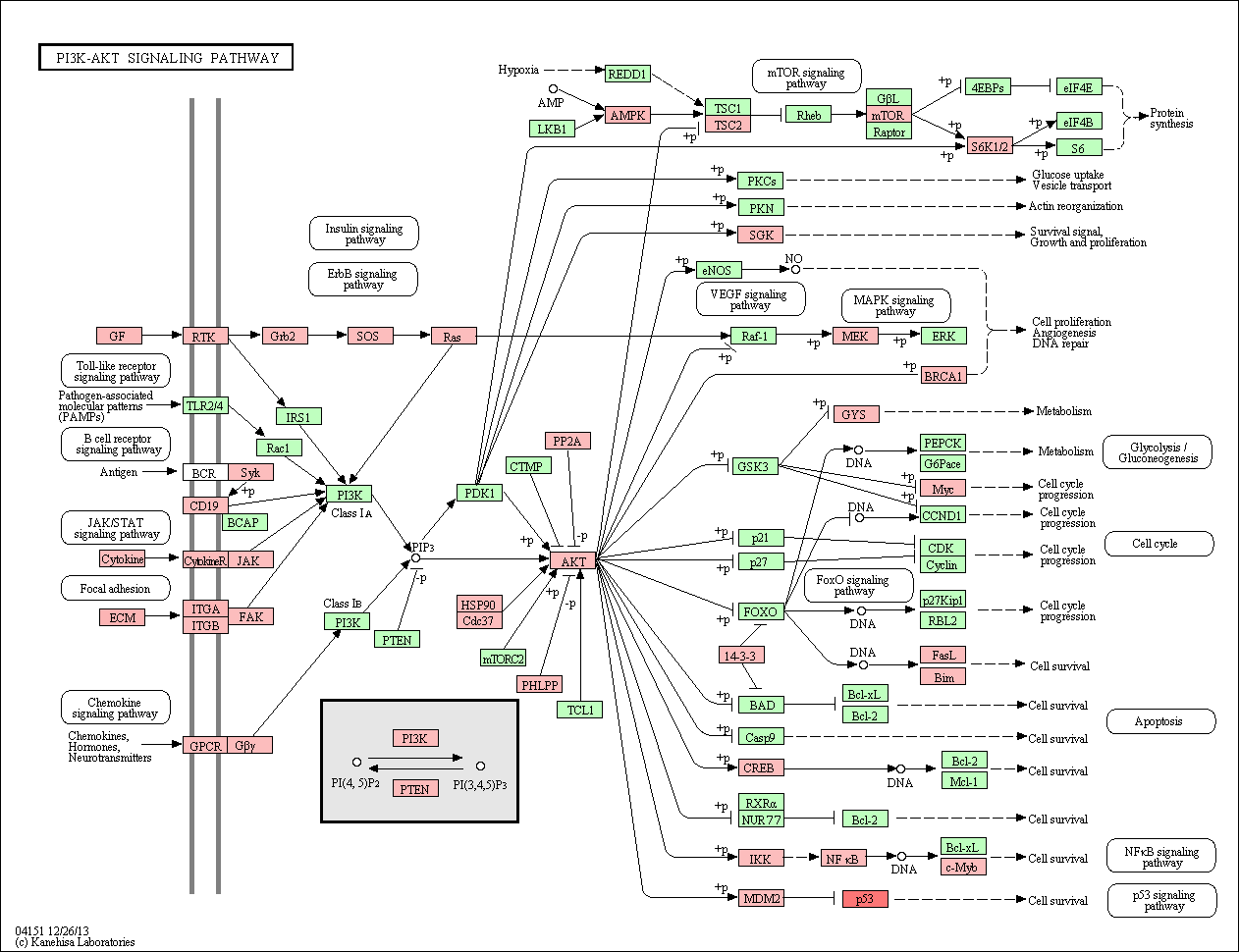
**

**Figure S6. Pathway collaboration of Basal-like - hsa04151: PI3K-Akt signaling pathway.**

**
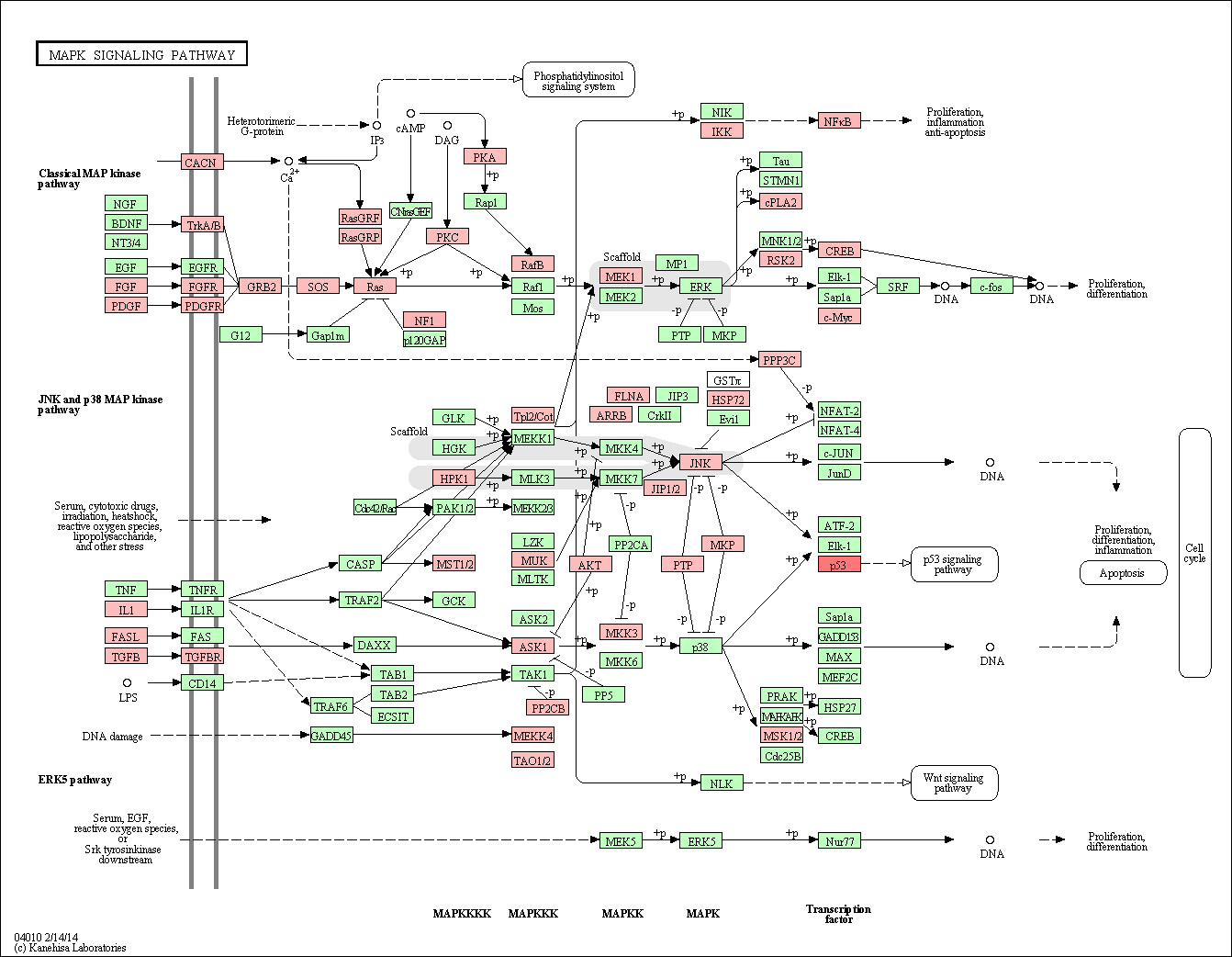
**

**Figure S7. Pathway collaboration of Basal-like - hsa04010: MAPK signaling pathway.**

**
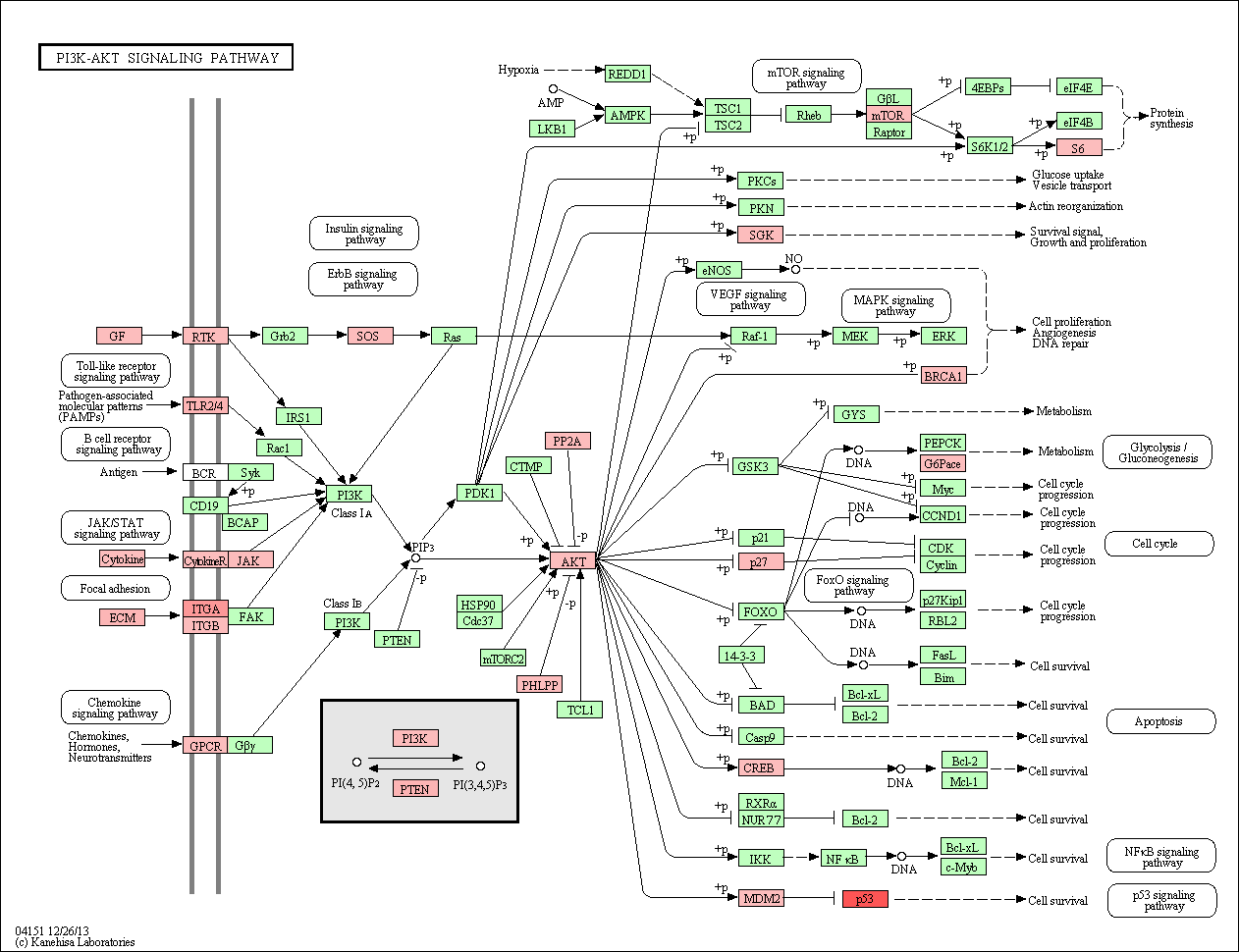
**

**Figure S8. Pathway collaboration of HER2+ - hsa04151: PI3K-Akt signaling pathway.**

**
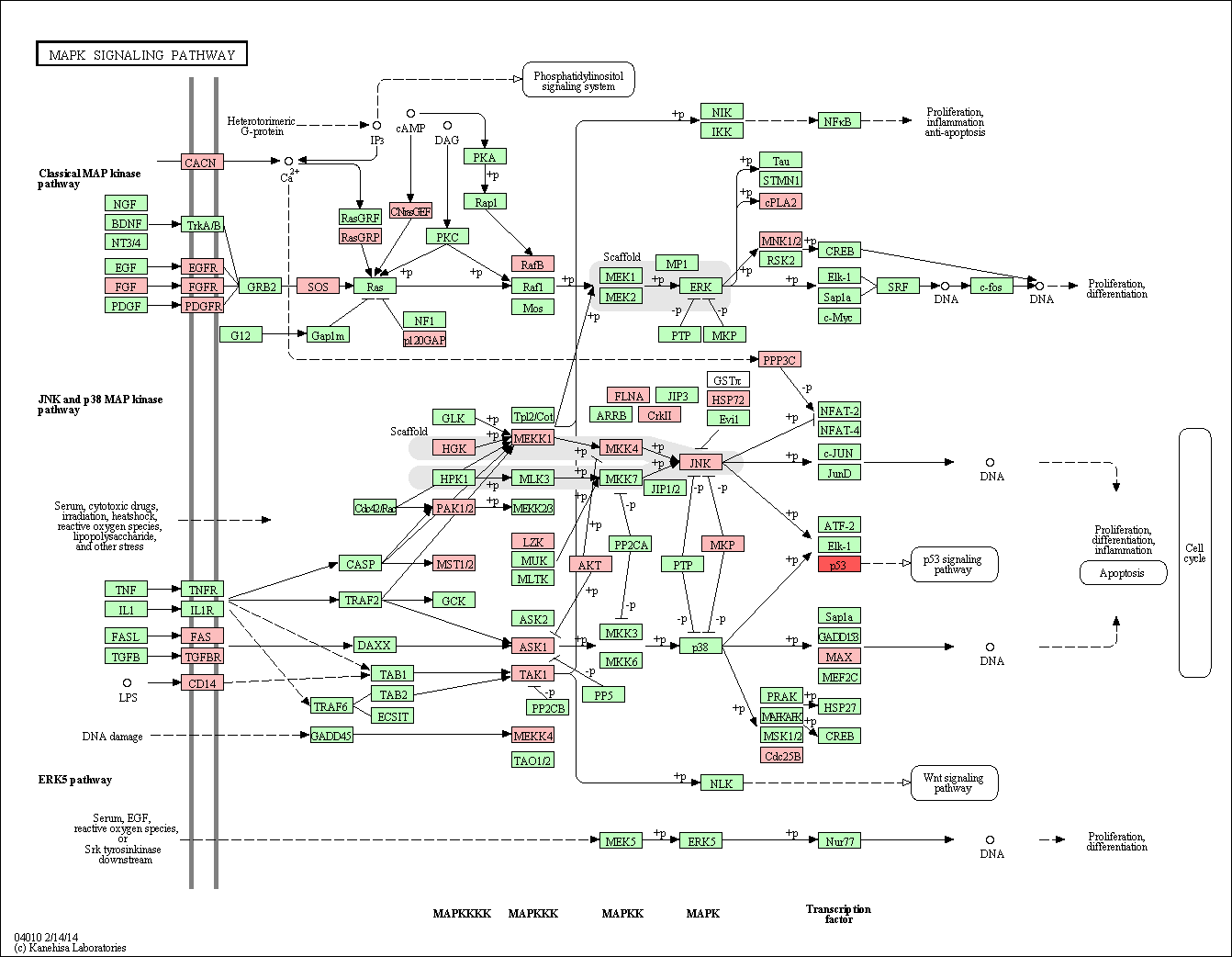
**

**Figure S9.** **Pathway collaboration of HER2+ - hsa04010: MAPK signaling pathway.**


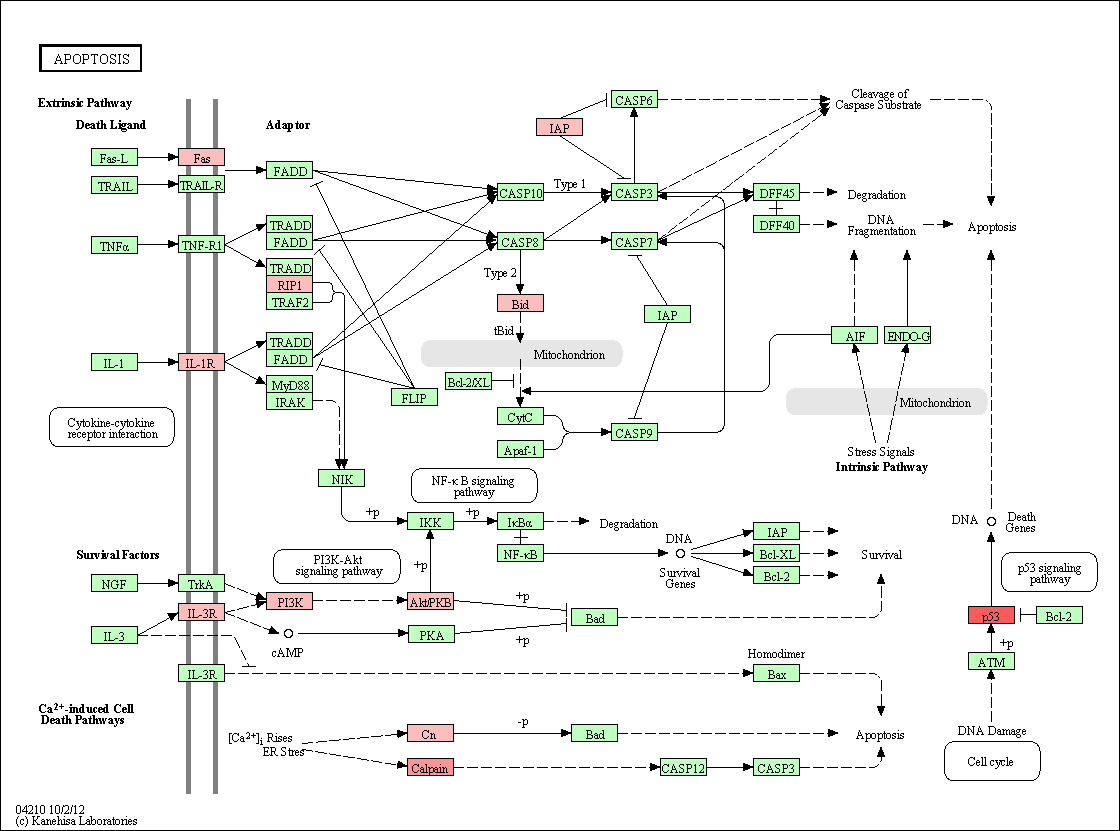


**Figure S10.** **Pathway collaboration of HER2+ - hsa04210: Apoptosis.**


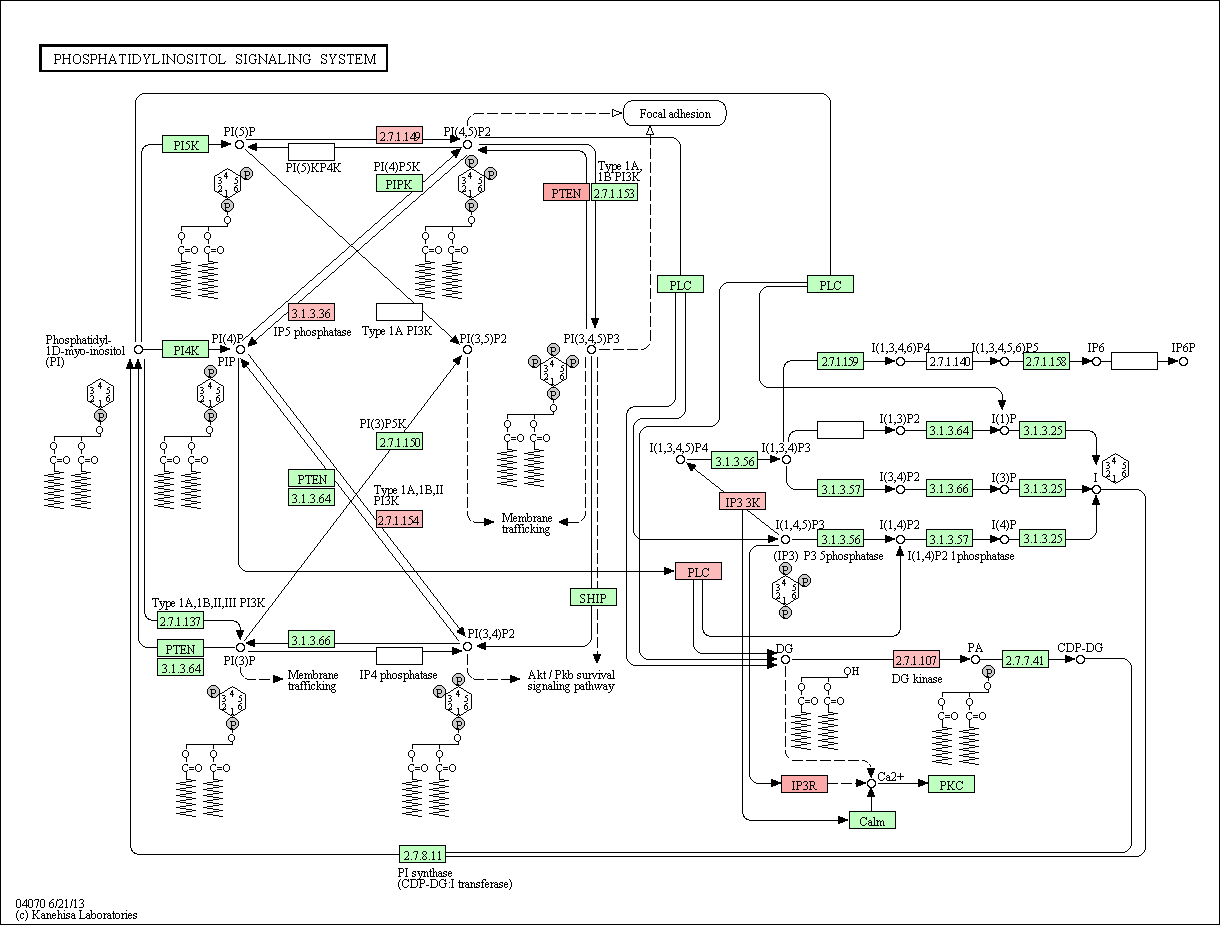


**Figure S11.** **Pathway collaboration of HER2+ - hsa04070: Phosphatidylinositol signaling system.**


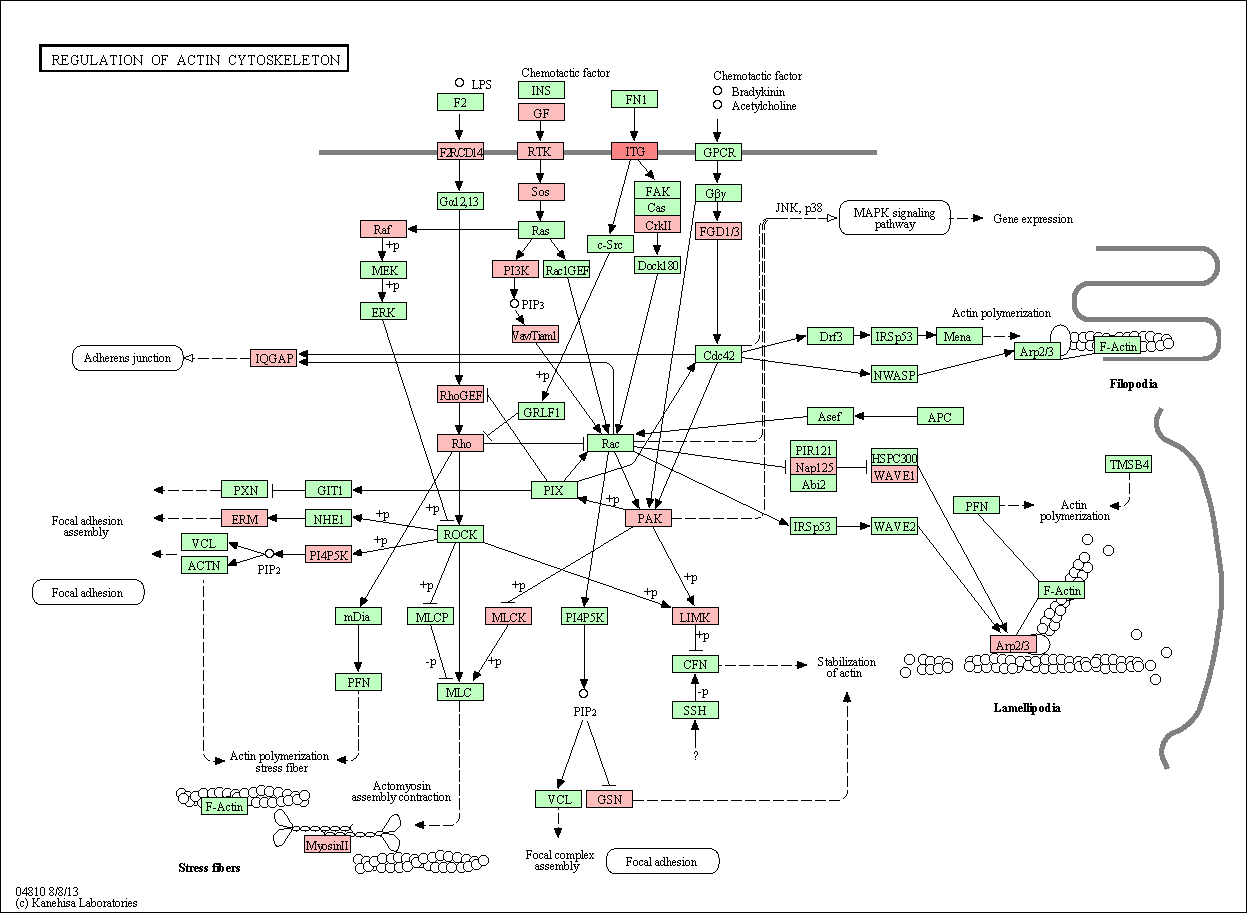


**Figure S12.** **Pathway collaboration of HER2+ - hsa04810: Regulation of actin cytoskeleton.**


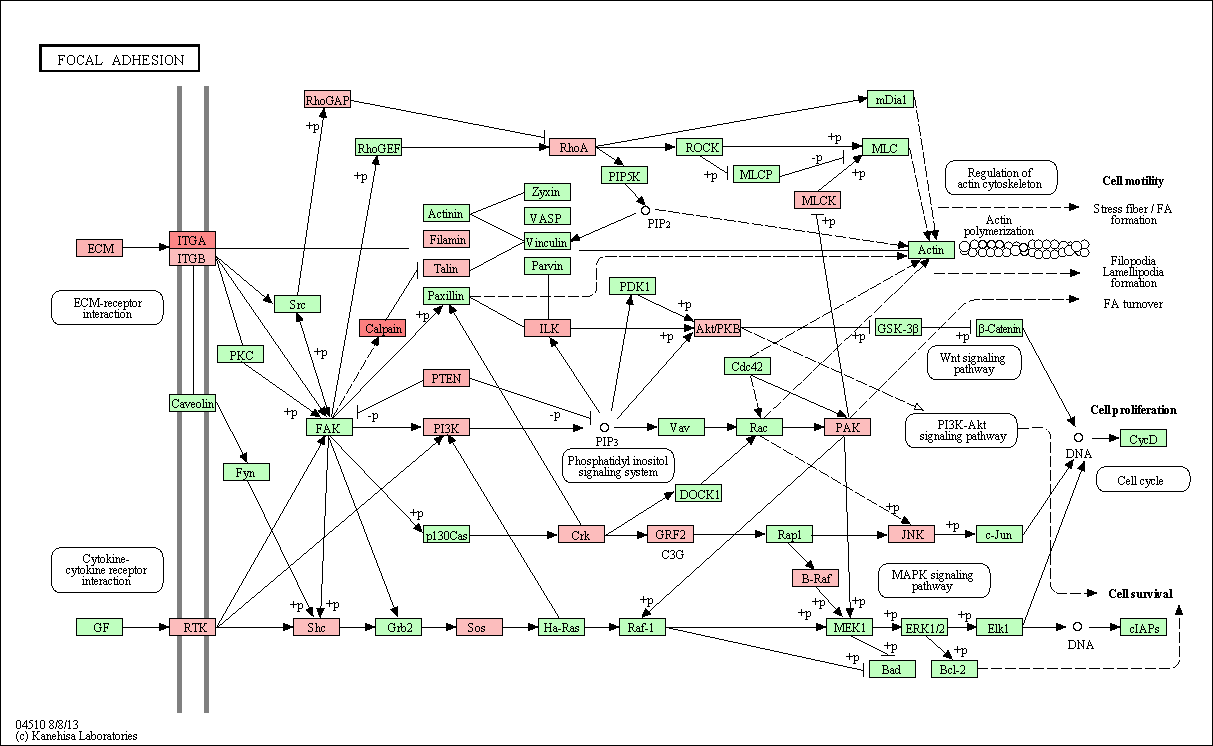


**Figure S13.** **Pathway collaboration of HER2+ - hsa04510: Focal adhesion.**


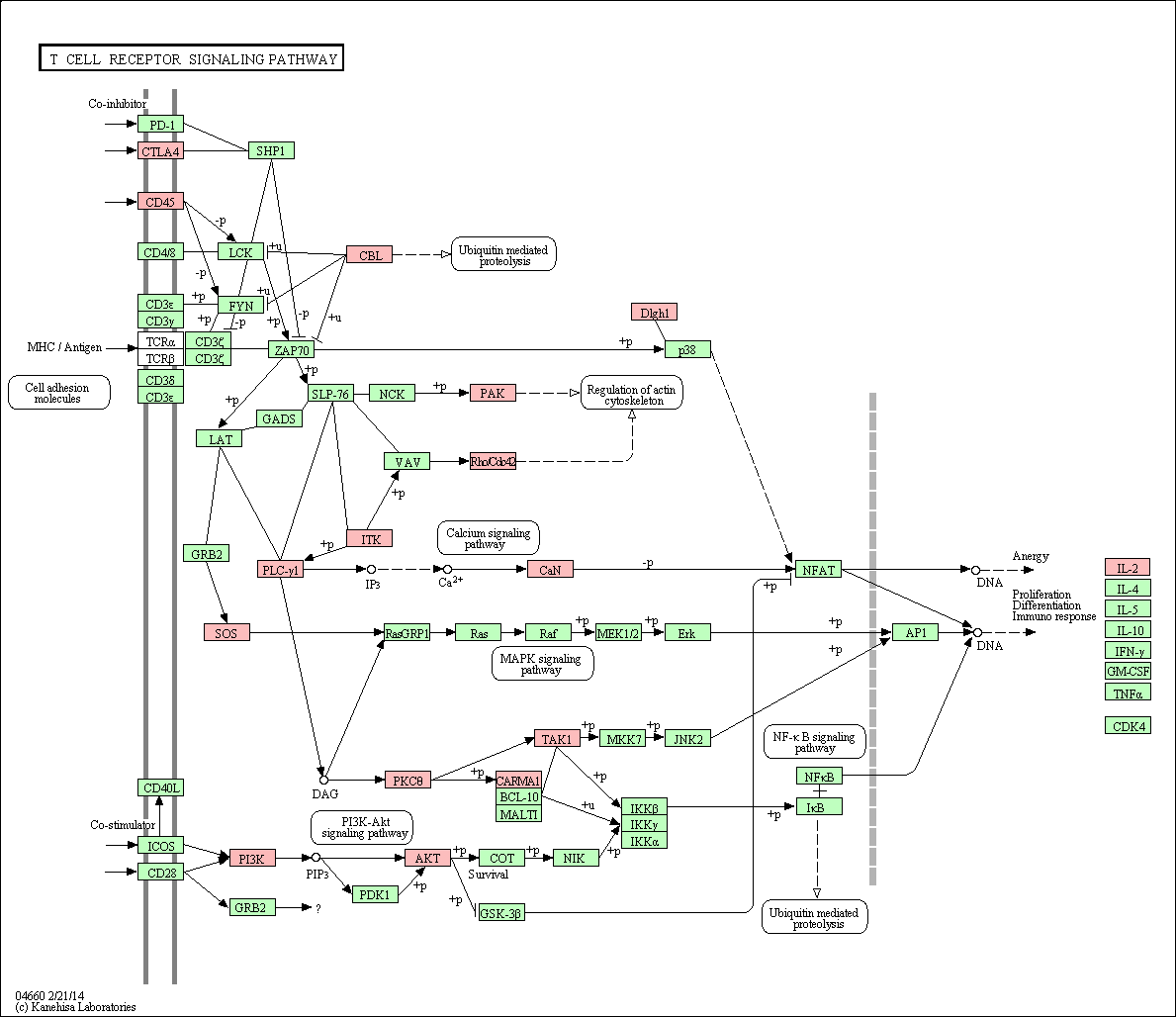


**Figure S14.** **Pathway collaboration of HER2+ - hsa04660: T cell receptor signaling pathway.**


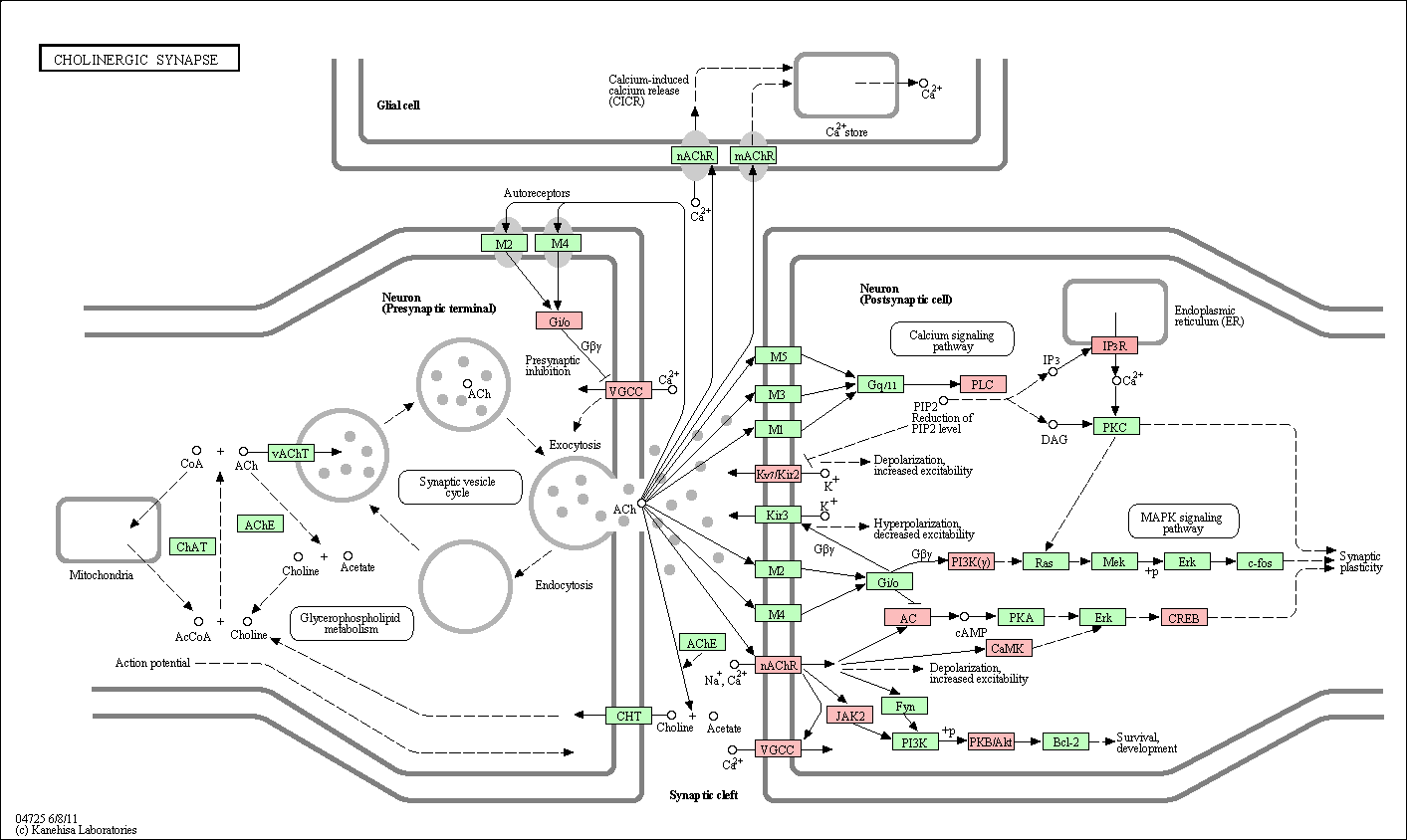


**Figure S15.** **Pathway collaboration of HER2+ - hsa04725: Cholinergic synapse.**

**
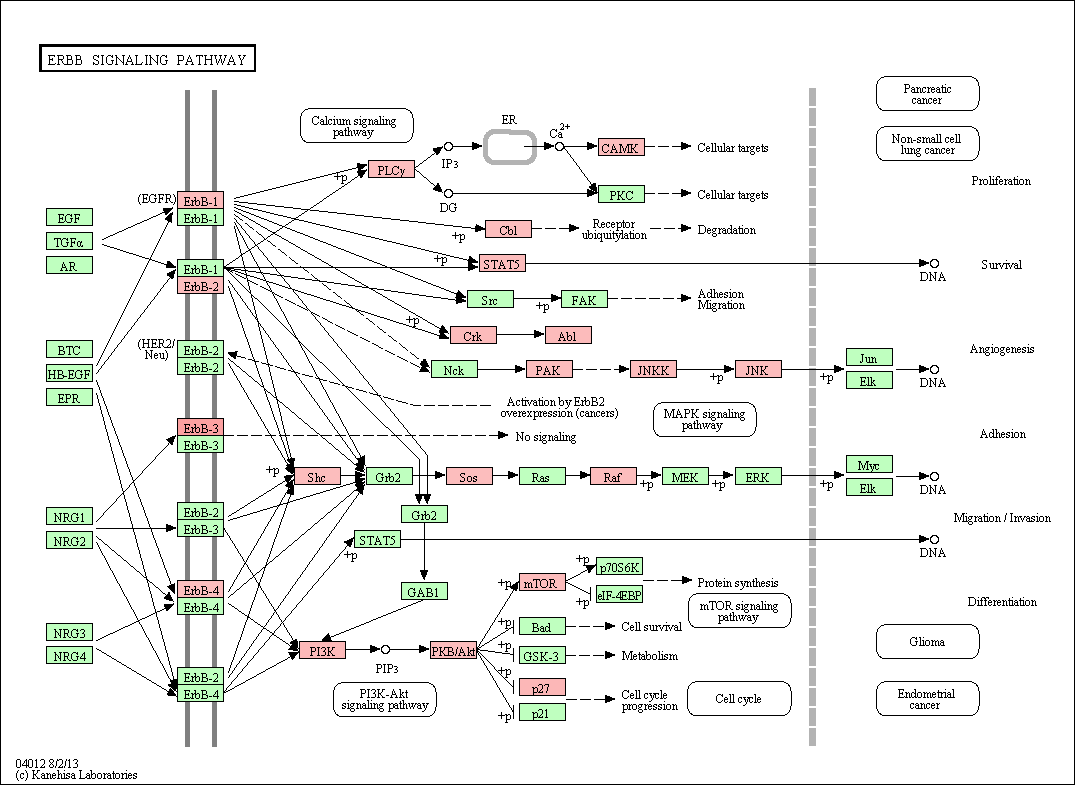
**

**Figure S16.** **Pathway collaboration of HER2+ - hsa04012: ErbB signaling pathway.**

**
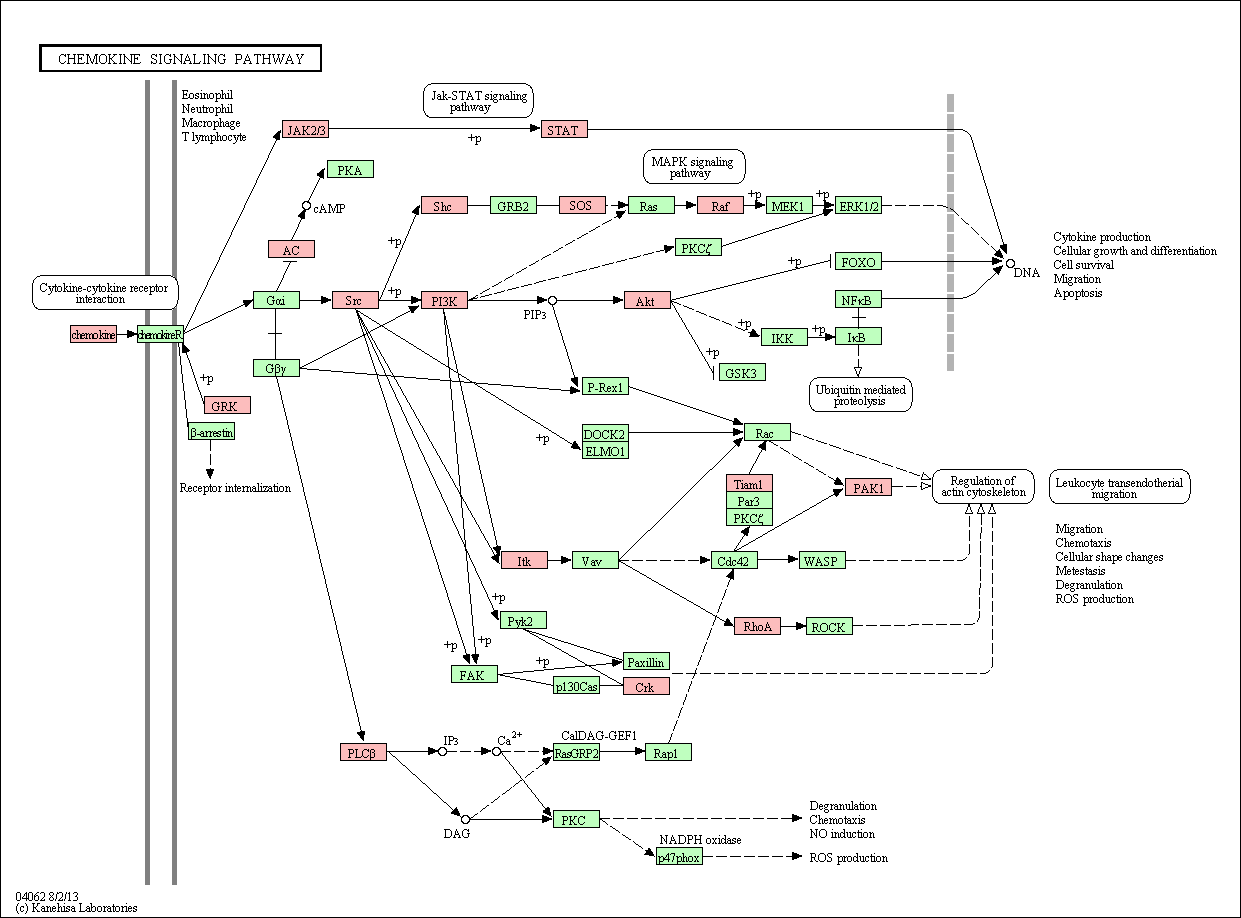
**

**Figure S17.** **Pathway collaboration of HER2+ - hsa04062: Chemokine signaling pathway.**

**
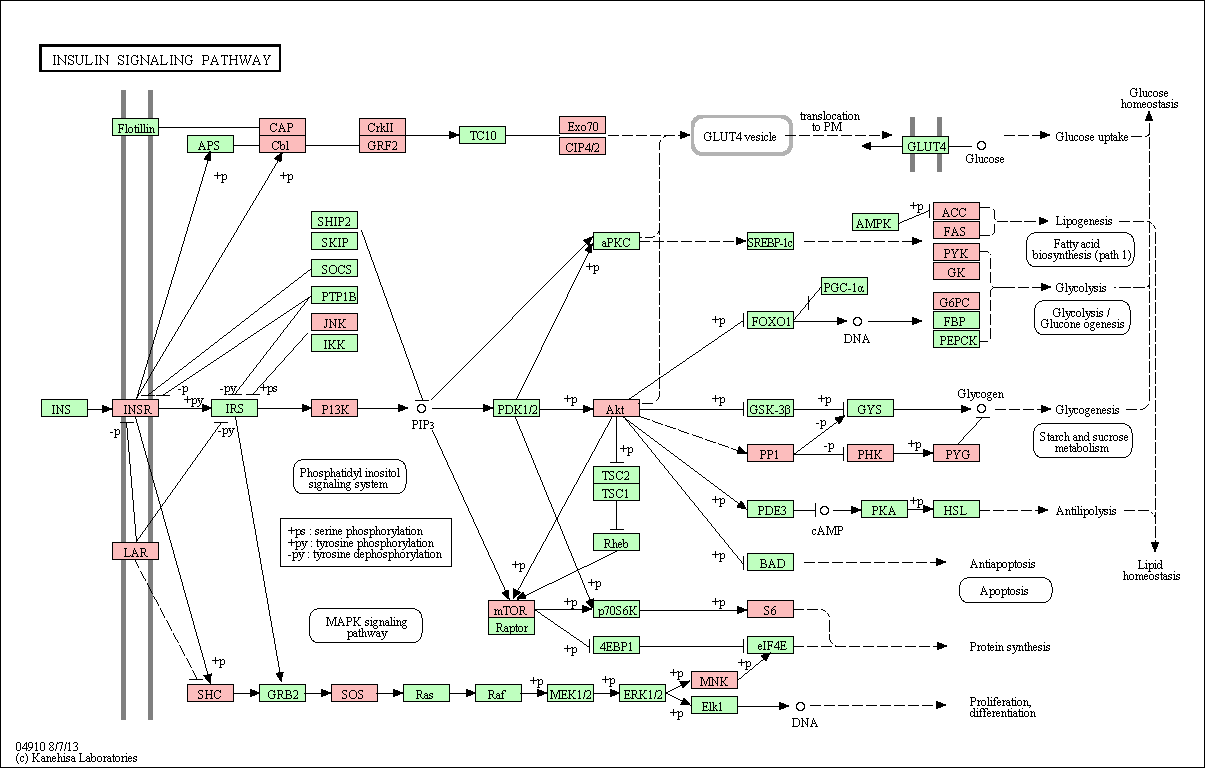
**

**Figure S18.** **Pathway collaboration of HER2+ - hsa04910: Insulin signaling pathway.**

**
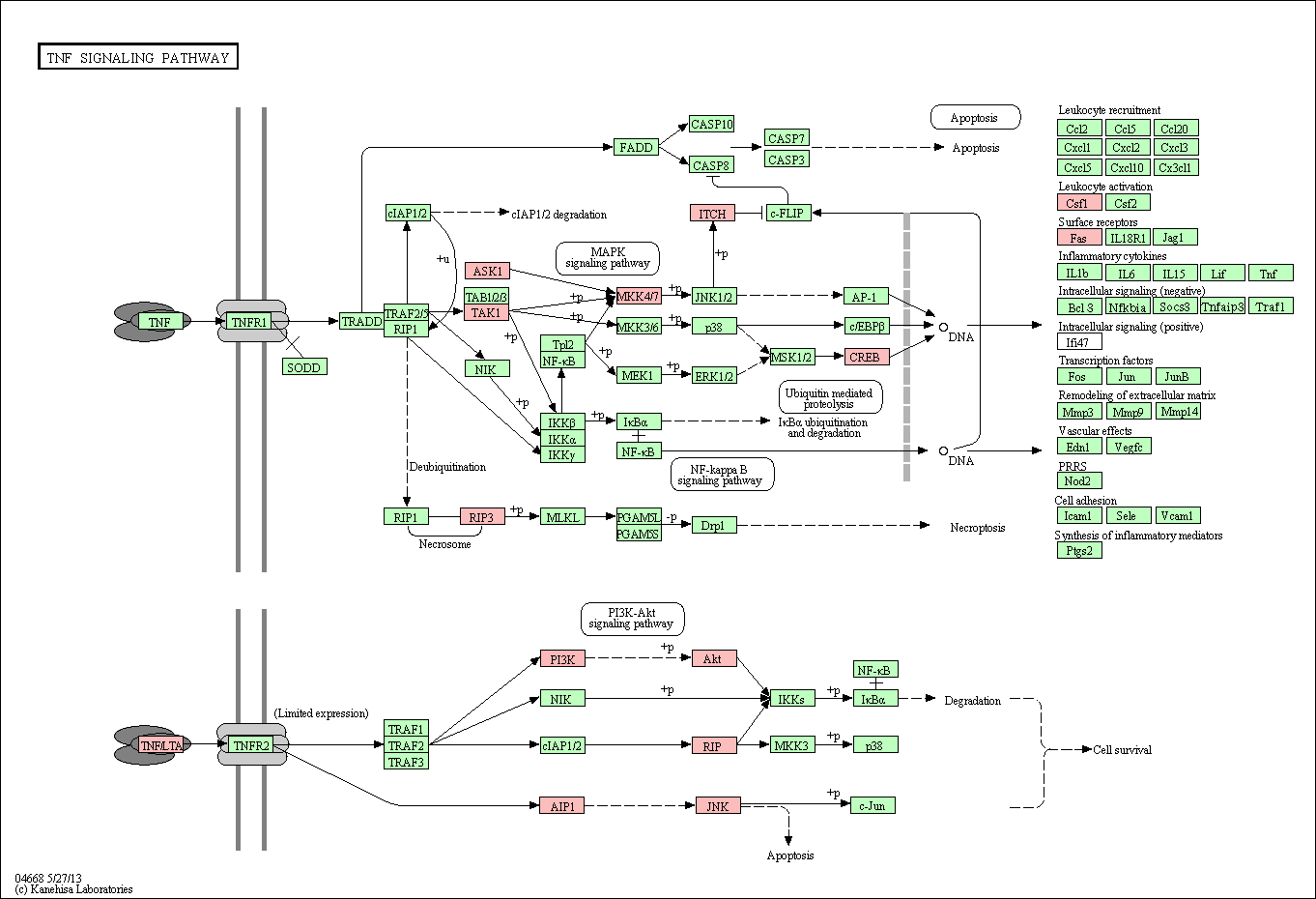
**

**Figure S19.** **Pathway collaboration of HER2+ - hsa04668: TNF signaling pathway.**

**
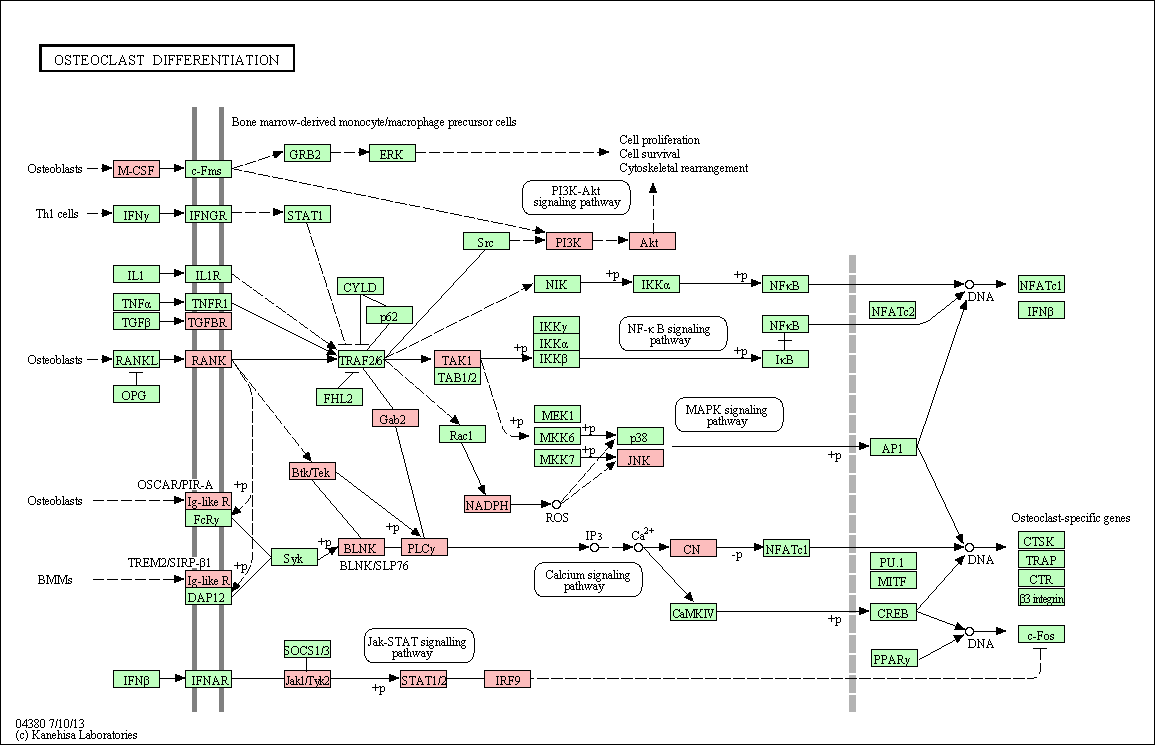
**

**Figure S20.** **Pathway collaboration of HER2+ - hsa04380: Osteoclast differentiation.**

**
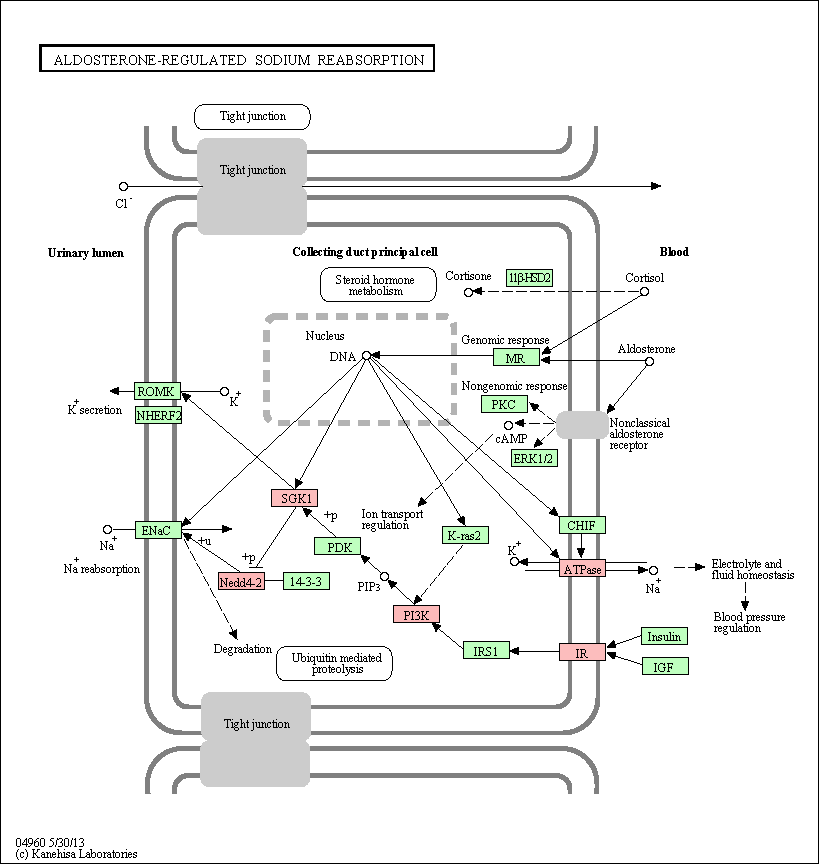
**

**Figure S21.** **Pathway collaboration of HER2+ - hsa04960: Aldosterone-regulated sodium reabsorption.**

**
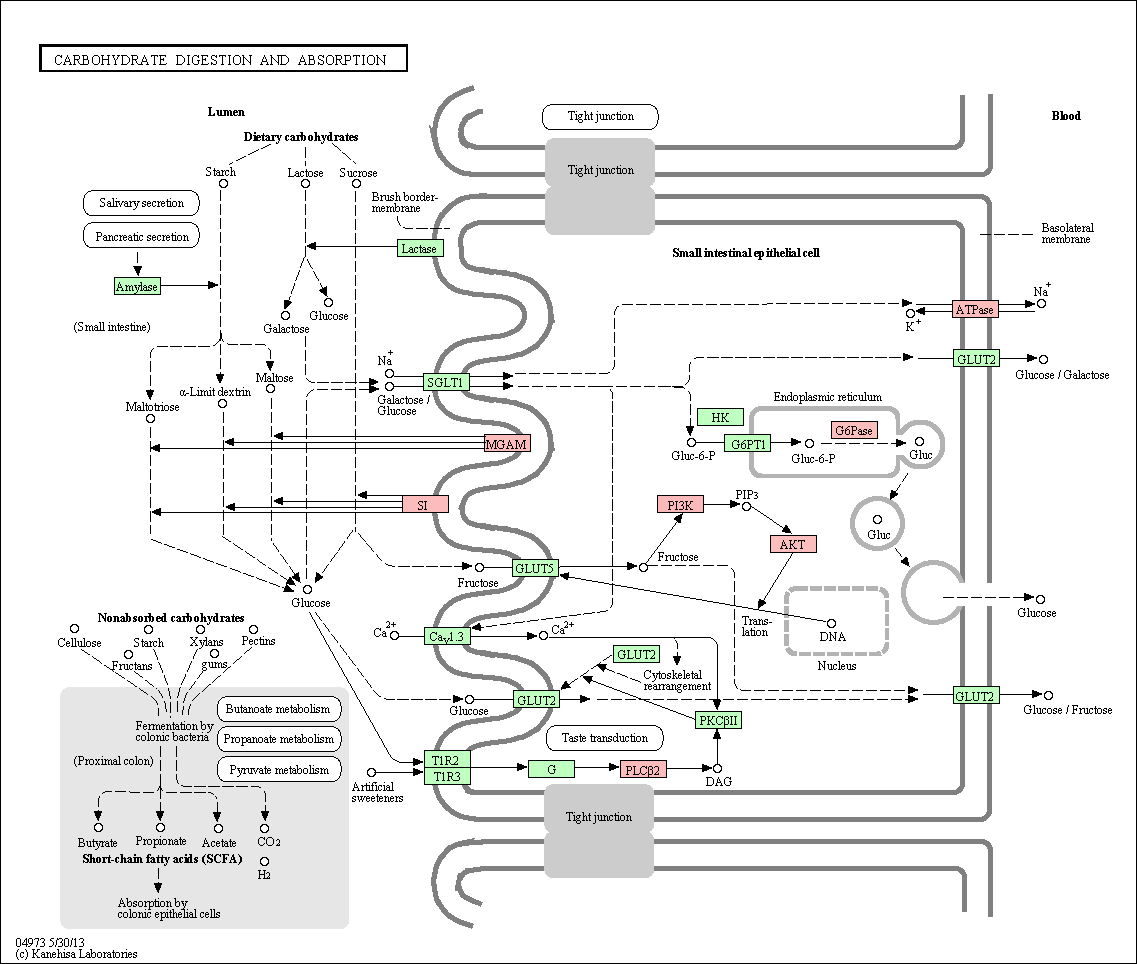
**

**Figure S22.** **Pathway collaboration of HER2+ - hsa04973: Carbohydrate digestion and absorption.**

**
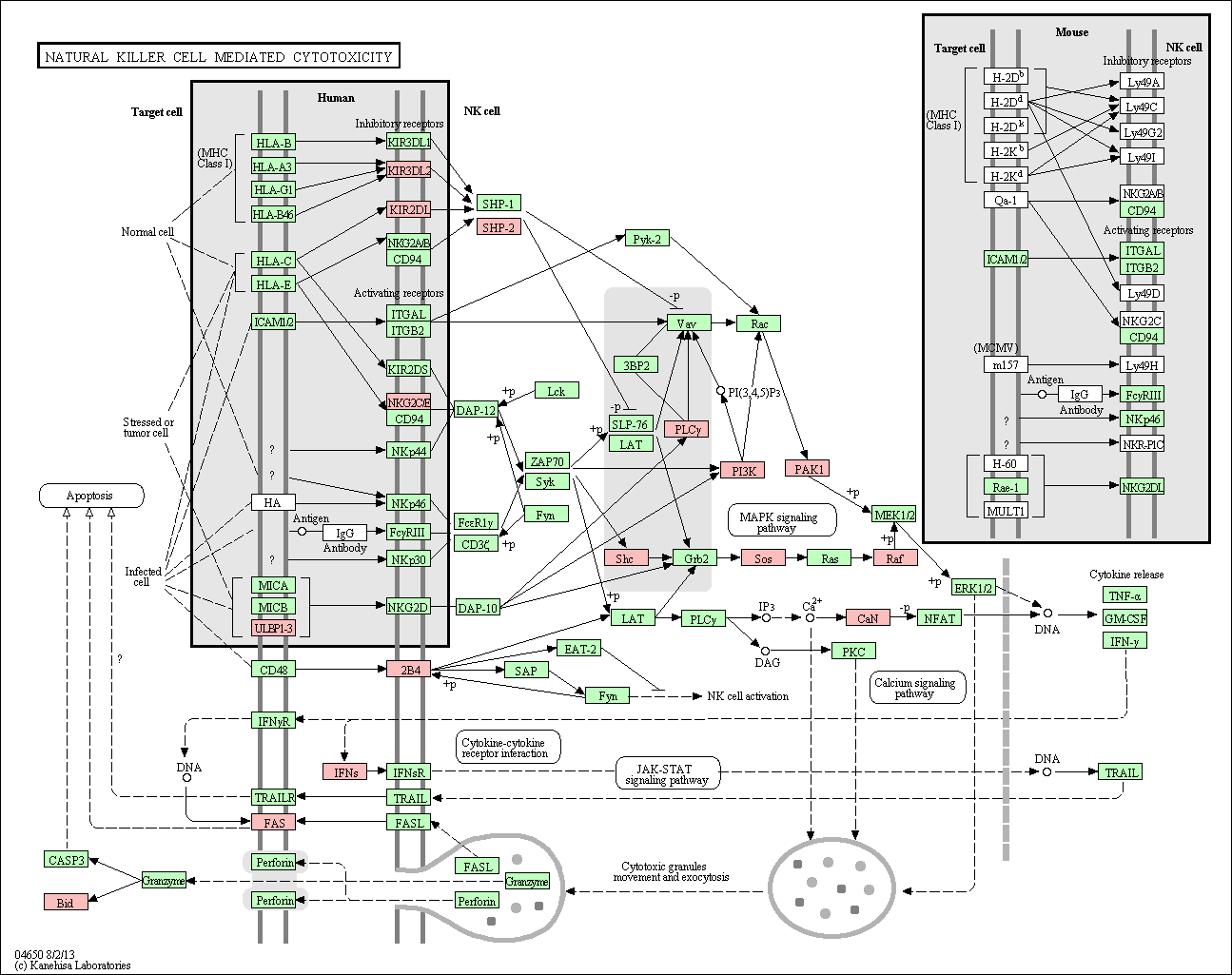
**

**Figure S23.** **Pathway collaboration of HER2+ - hsa04650: Natural killer cell mediated cytotoxicity.**

**
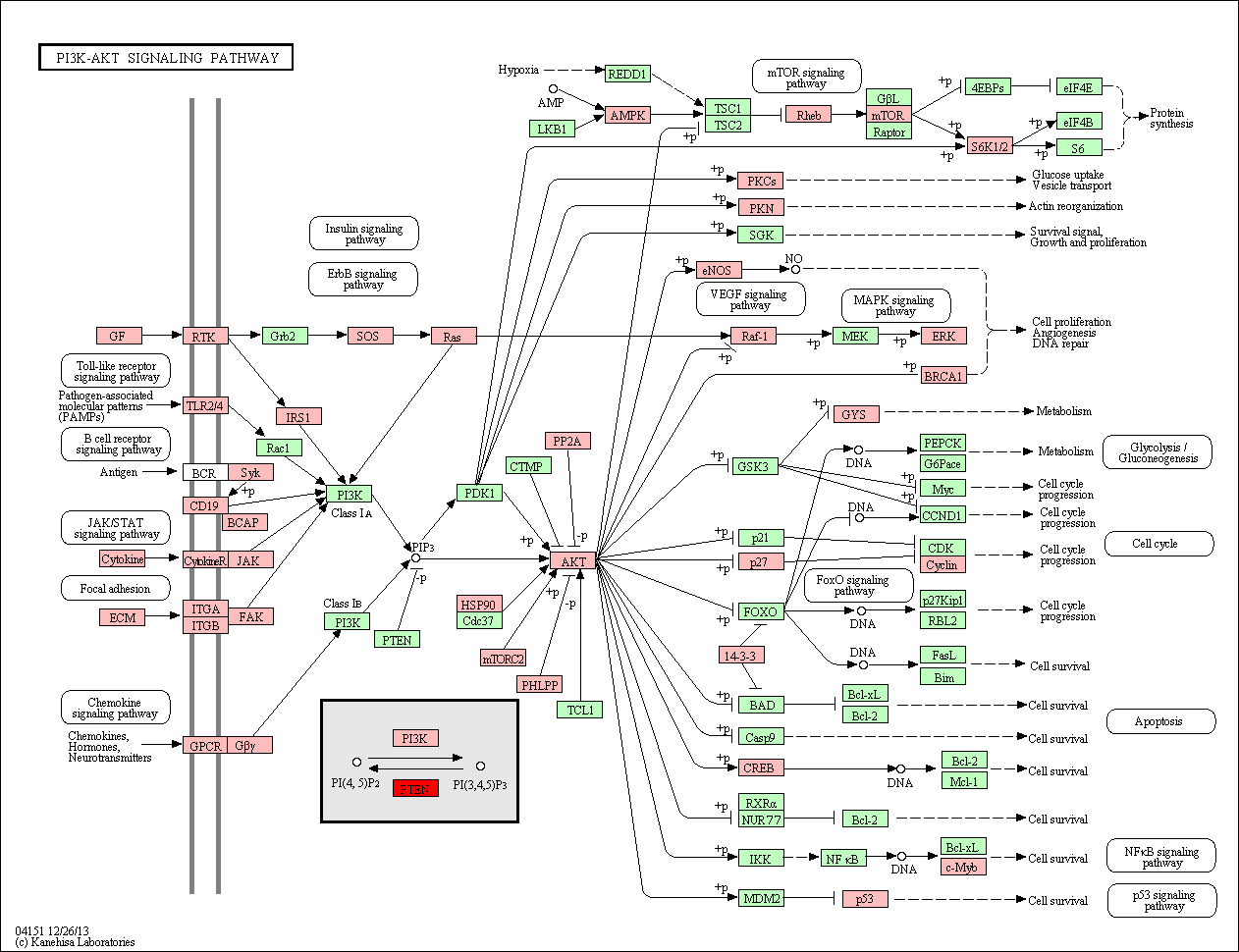
**

**Figure S24.** **Pathway collaboration of LuminalA - hsa04151: PI3K-Akt signaling pathway.**

**
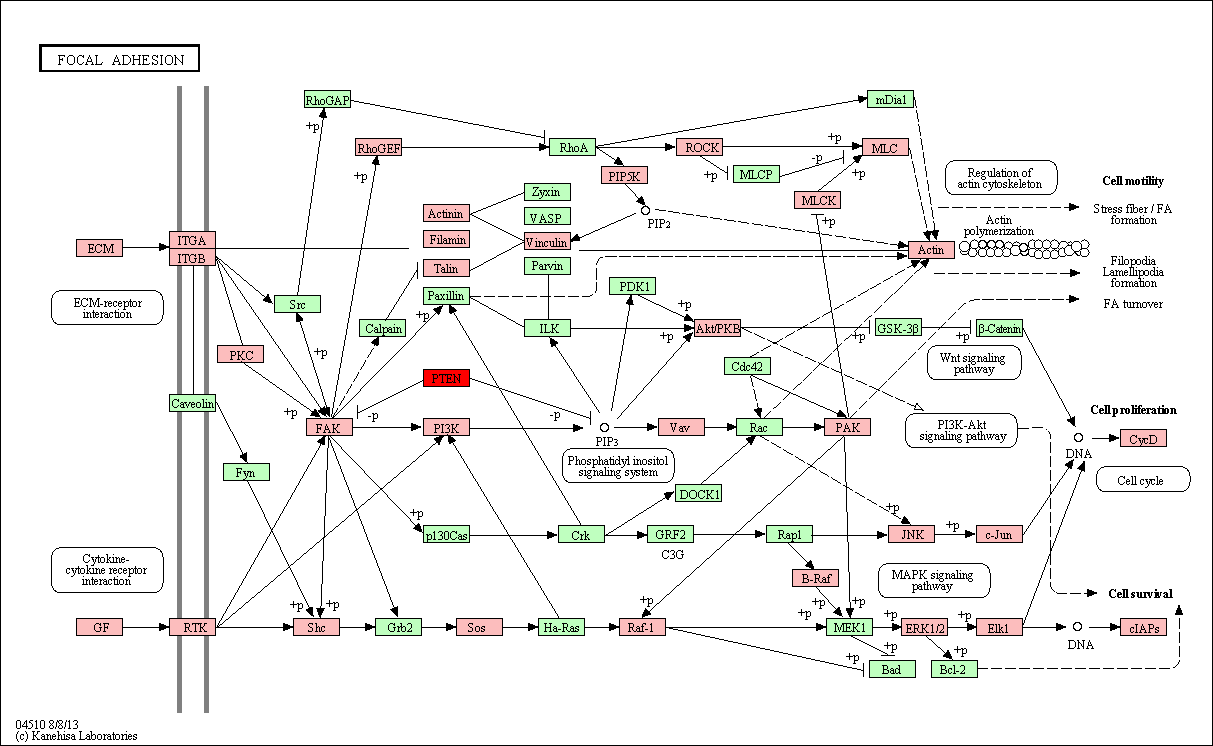
**

**Figure S25.** **Pathway collaboration of LuminalA - hsa04510: Focal adhesion.**

**
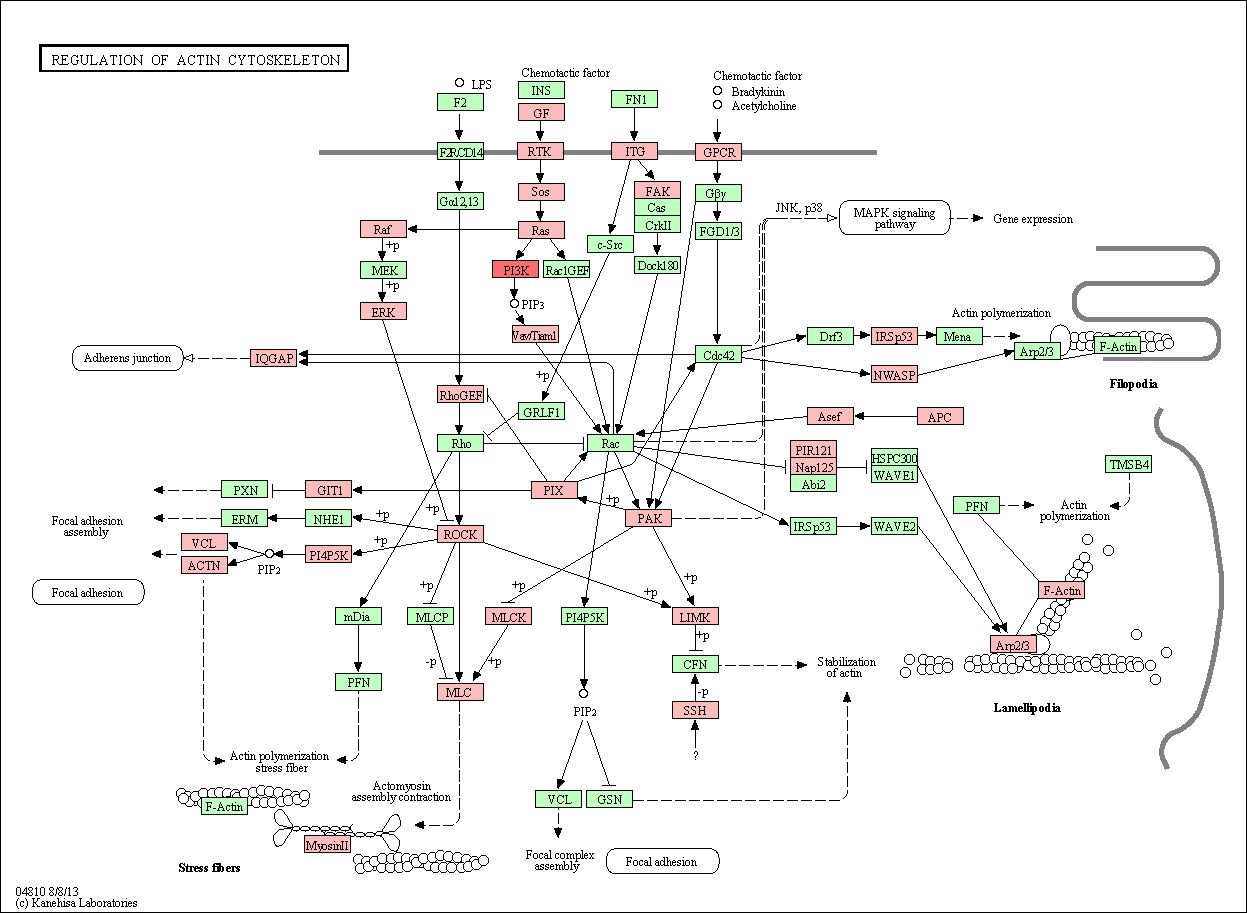
**

**Figure S26.** **Pathway collaboration of LuminalA - hsa04810: Regulation of actin cytoskeleton.**

**
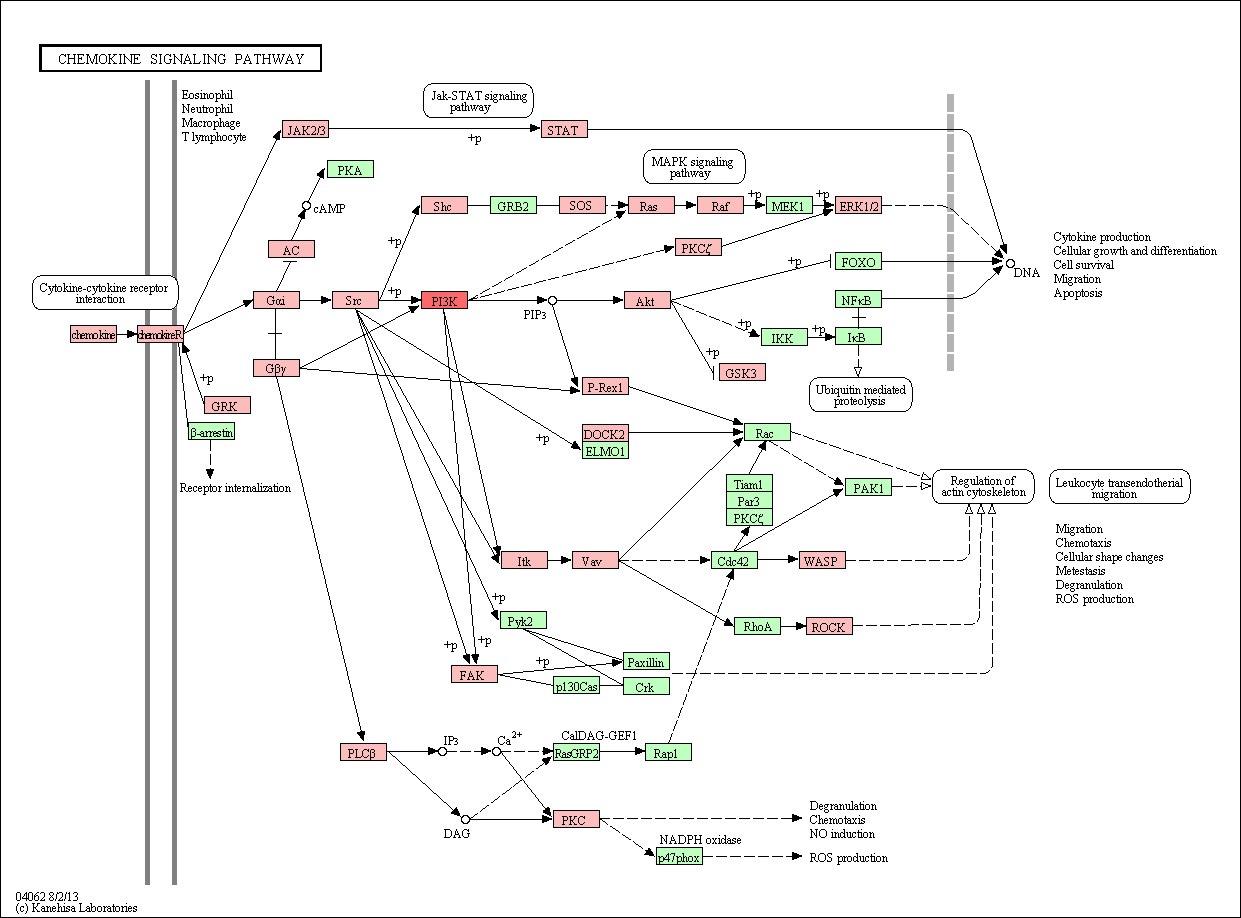
**

**Figure S27.** **Pathway collaboration of LuminalA - hsa04062: Chemokine signaling pathway.**

**
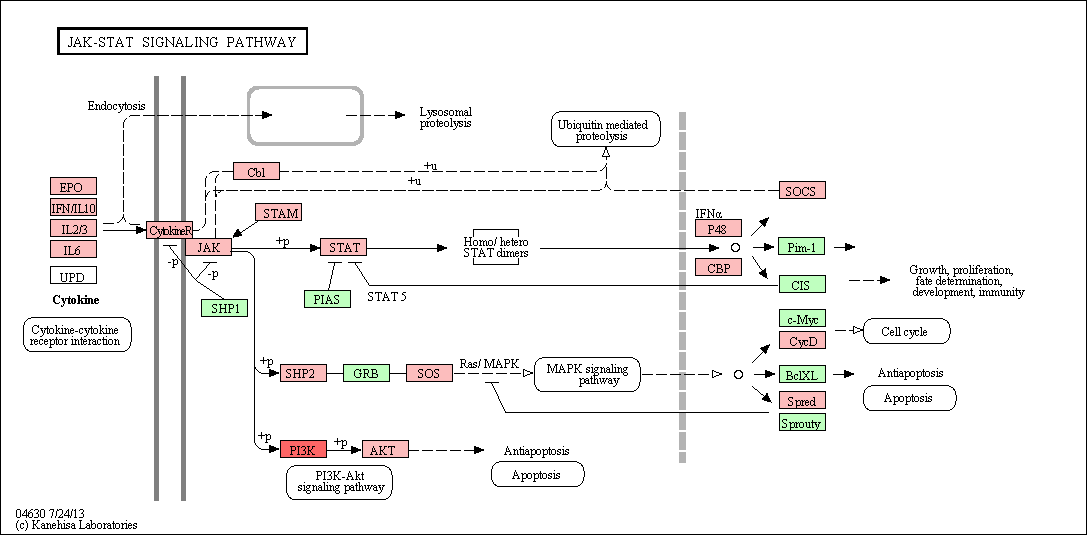
**

**Figure S28.** **Pathway collaboration of LuminalA - hsa04630: Jak-STAT signaling pathway.**

**
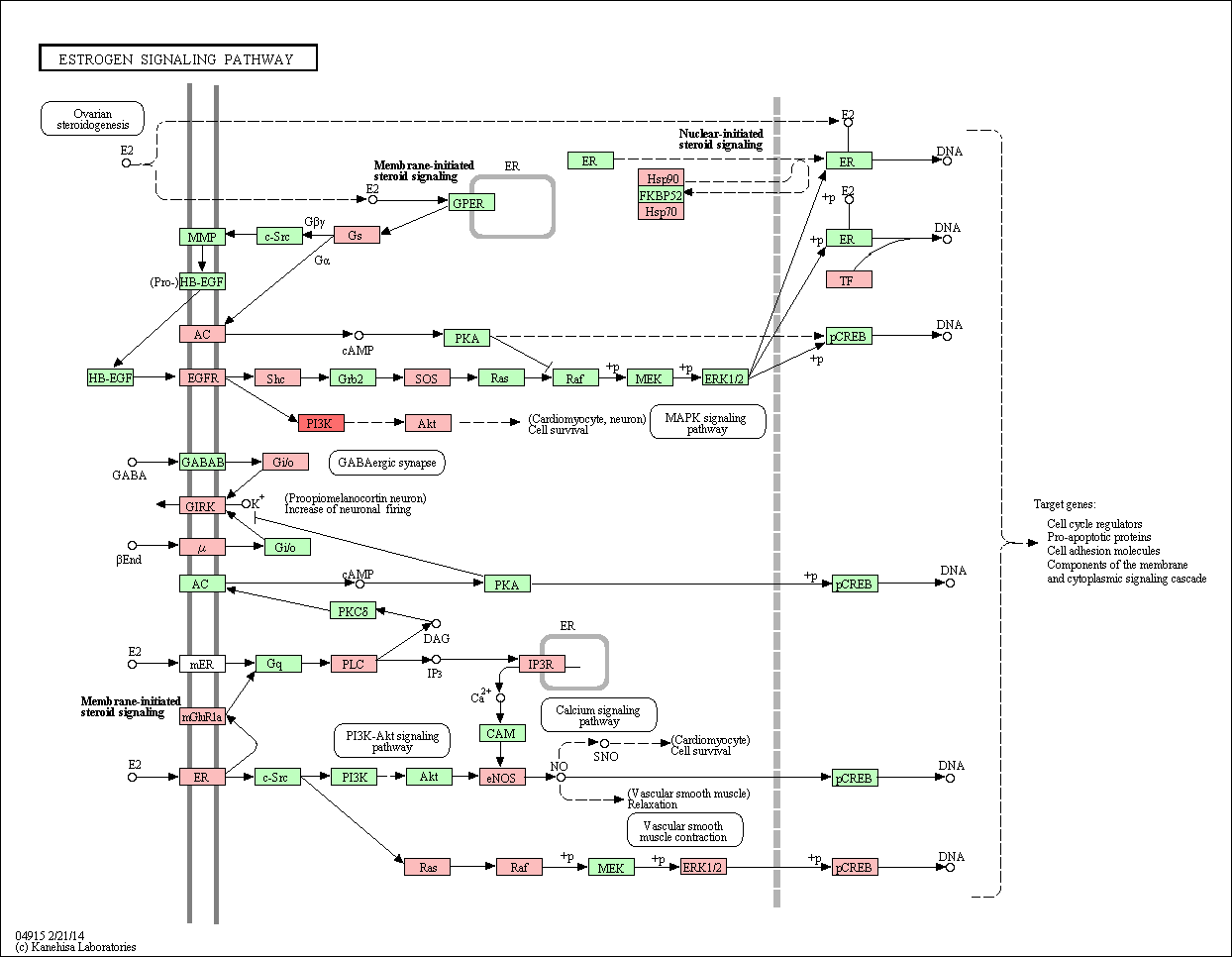
**

**Figure S29.** **Pathway collaboration of LuminalA - hsa04915: Estrogen signaling pathway.**

**
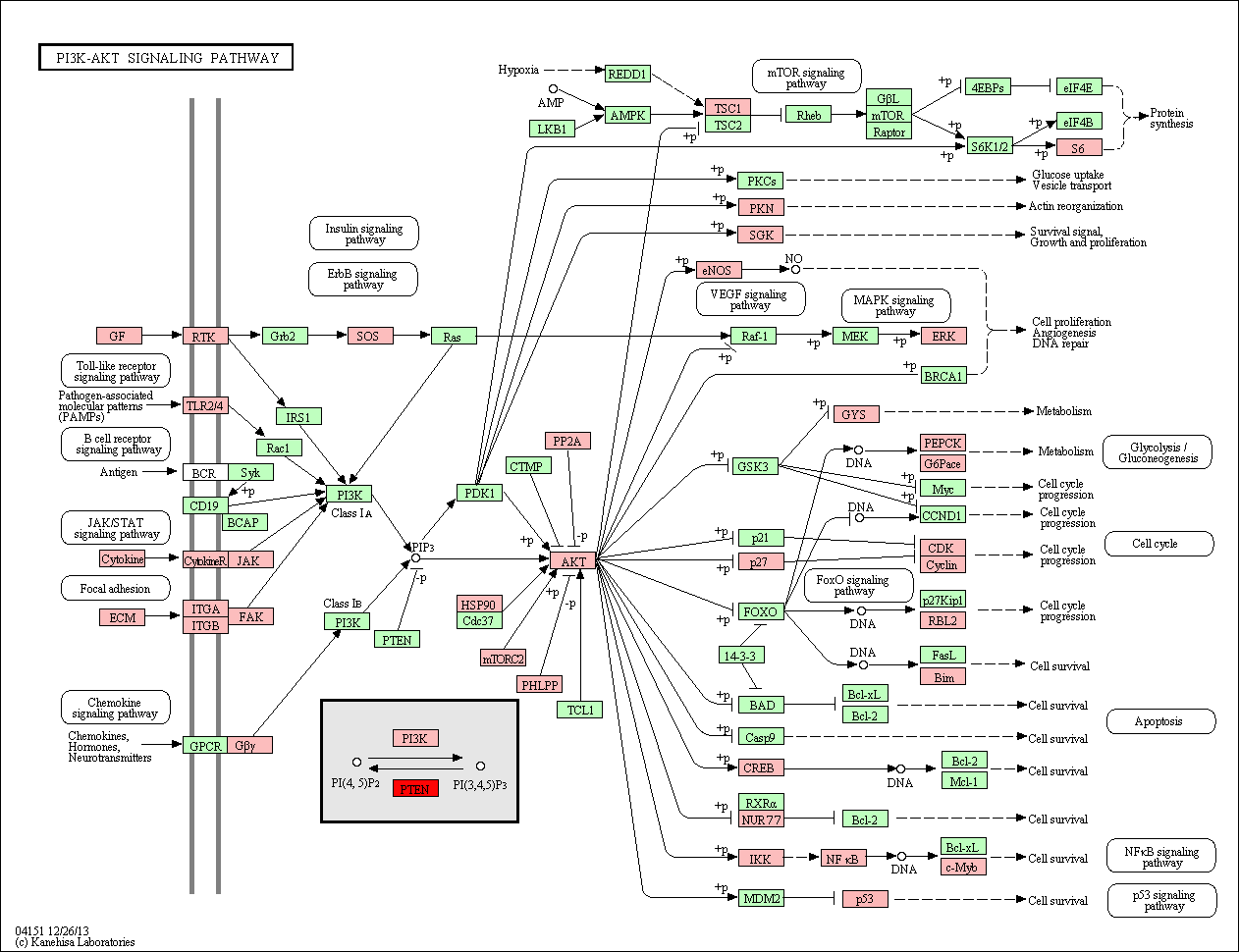
**

**Figure S30.** **Pathway collaboration of LuminalB - hsa04151: PI3K-Akt signaling pathway.**

**
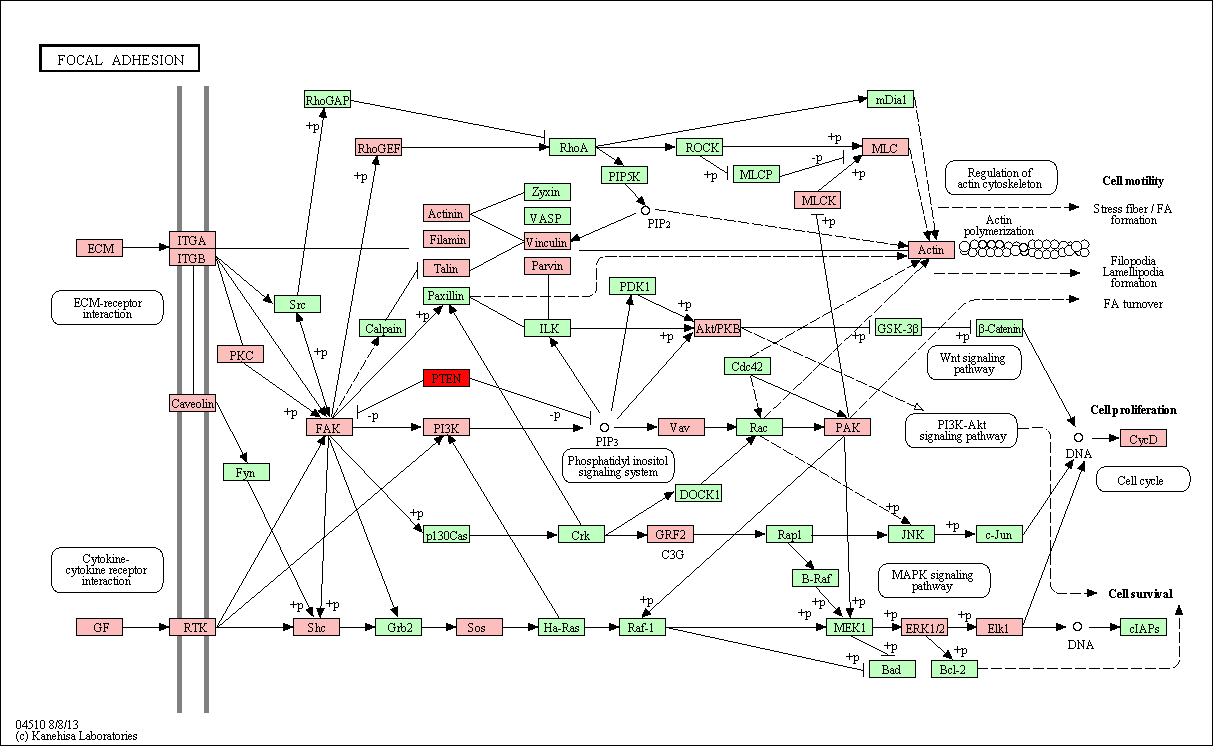
**

**Figure S31.** **Pathway collaboration of LuminalB - hsa04510: Focal adhesion.**

**
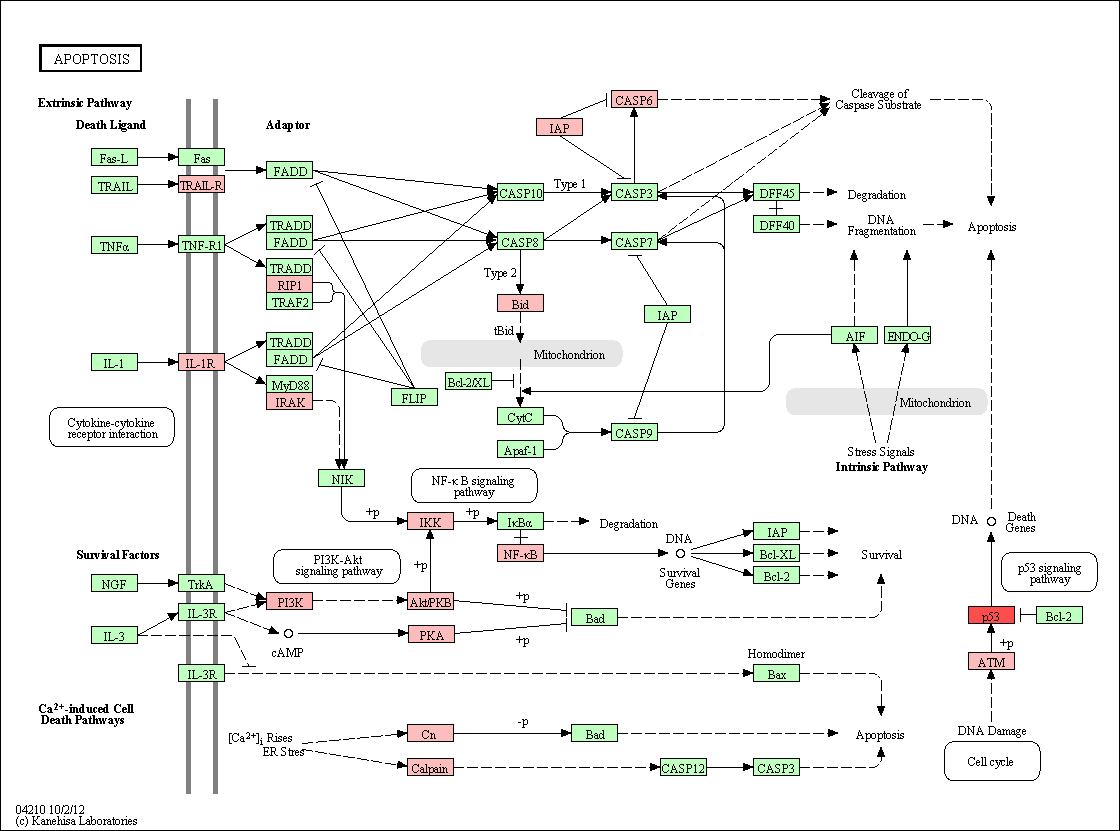
**

**Figure S32.** **Pathway collaboration of LuminalB - hsa04210: Apoptosis.**

**
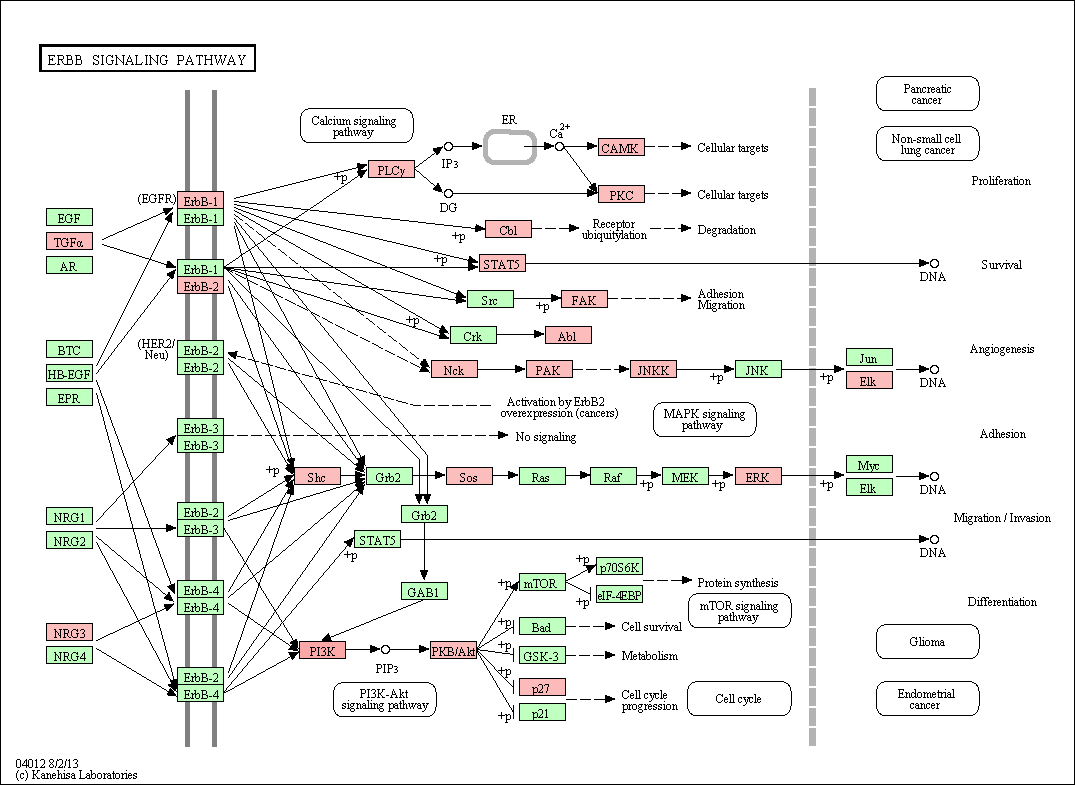
**

**Figure S33.** **Pathway collaboration of LuminalB - hsa04012: ErbB signaling pathway.**

**
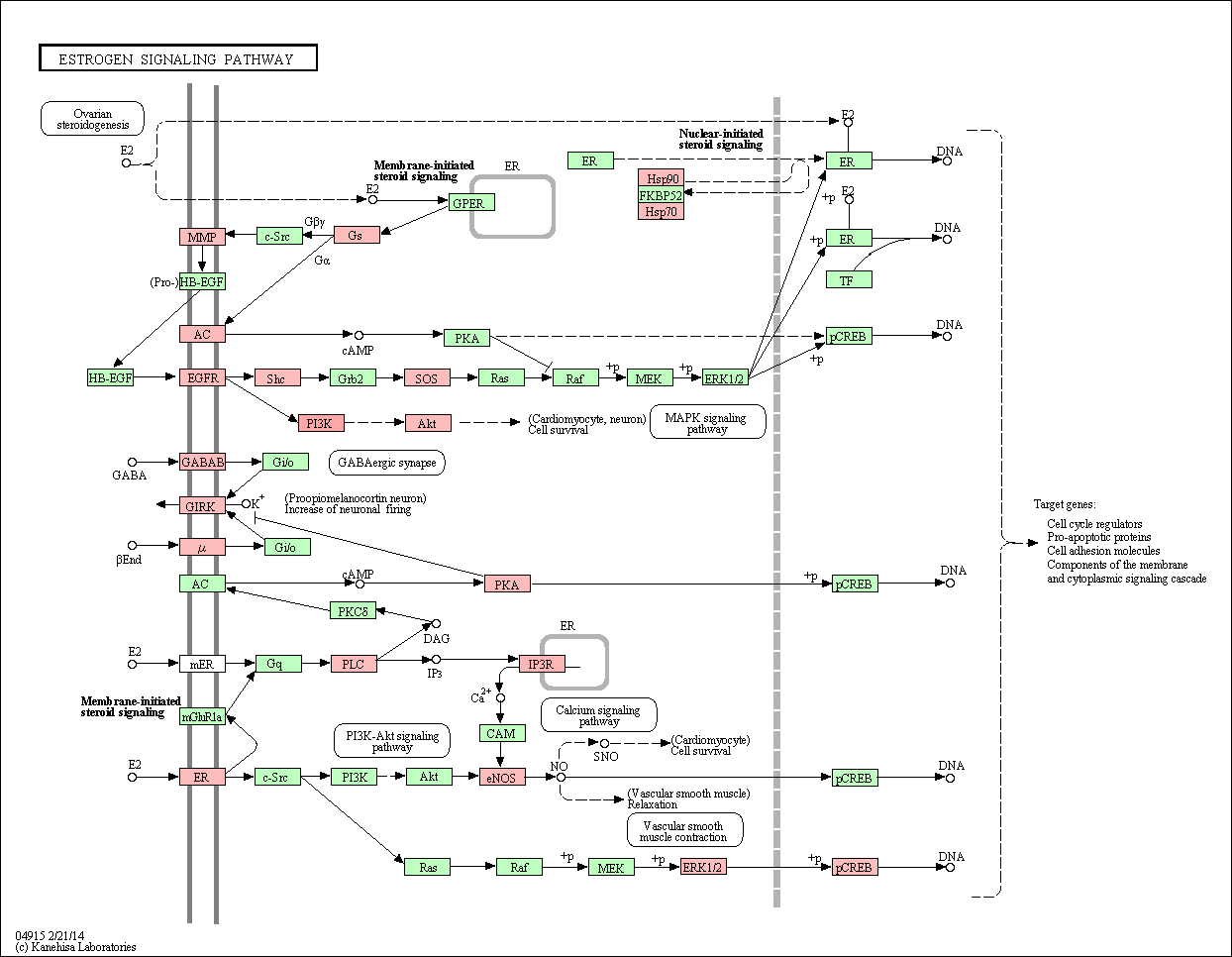
**

**Figure S34.** **Pathway collaboration of LuminalB - hsa04915: Estrogen signaling pathway.**

**
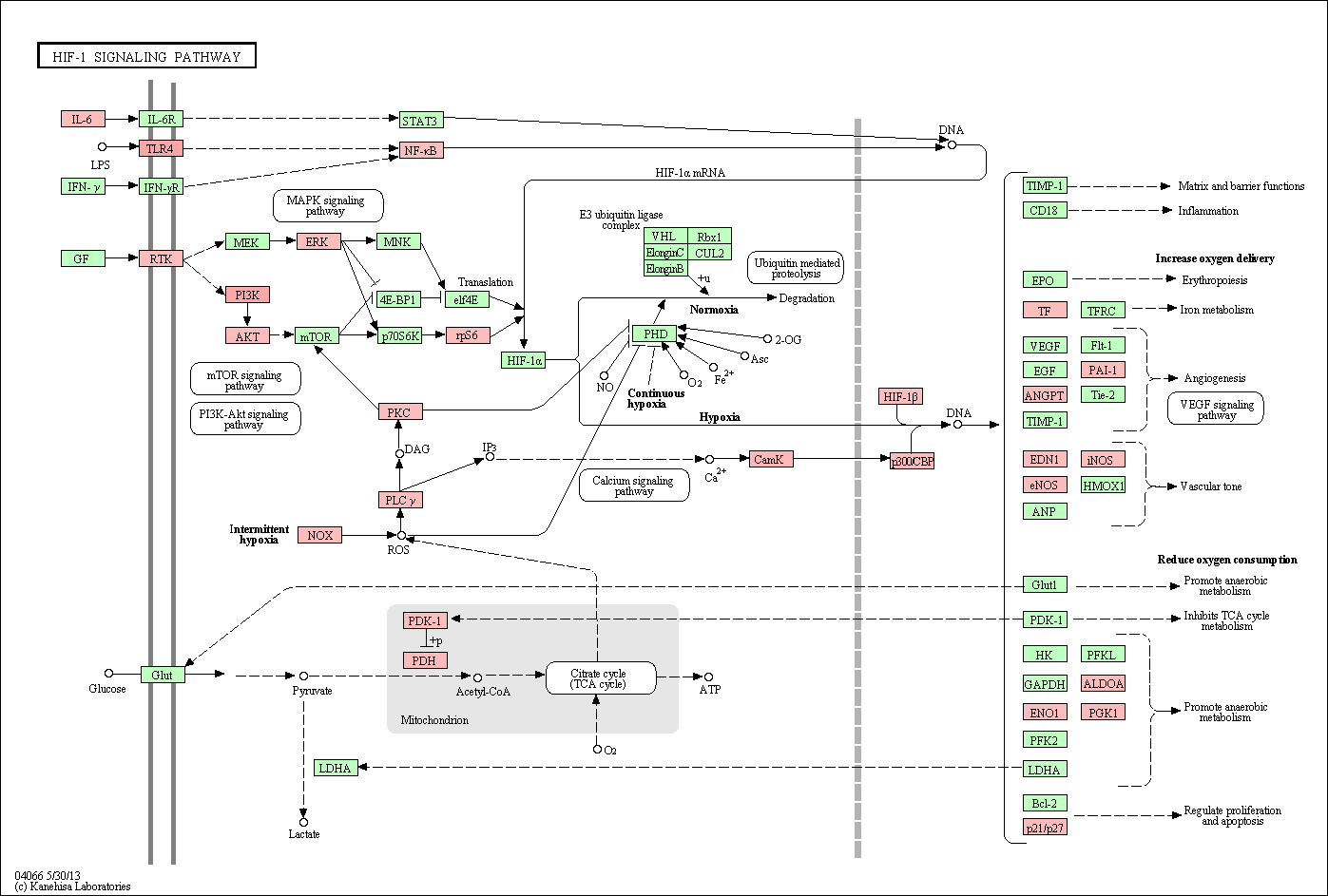
**

**Figure S35.** **Pathway collaboration of LuminalB - hsa04066: HIF-1 signaling pathway.**

**
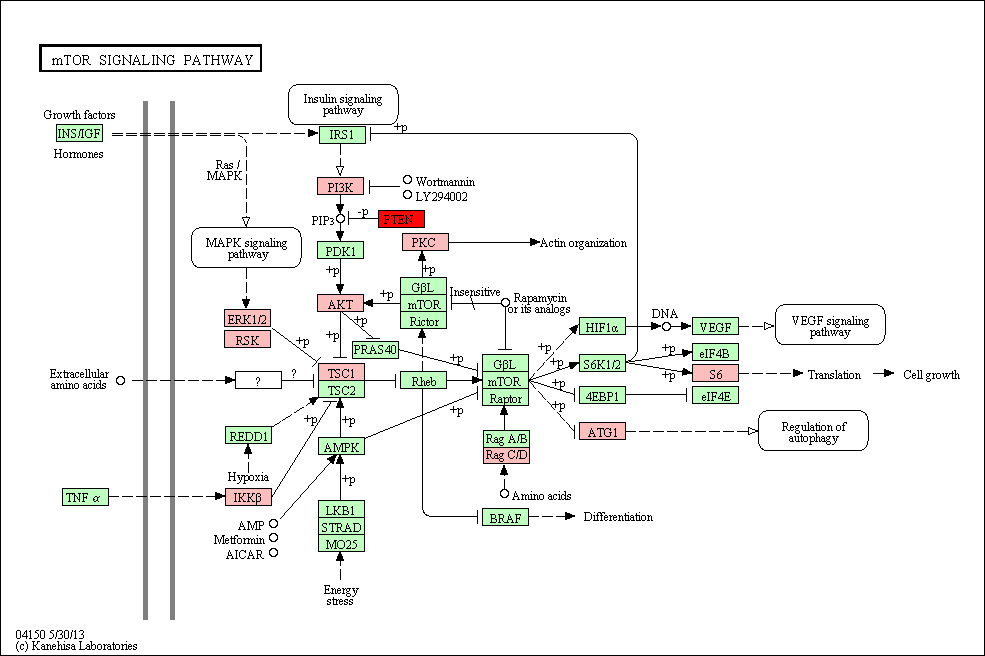
**

**Figure S36.** **Pathway collaboration of LuminalB - hsa04150: mTOR signaling pathway.**

**
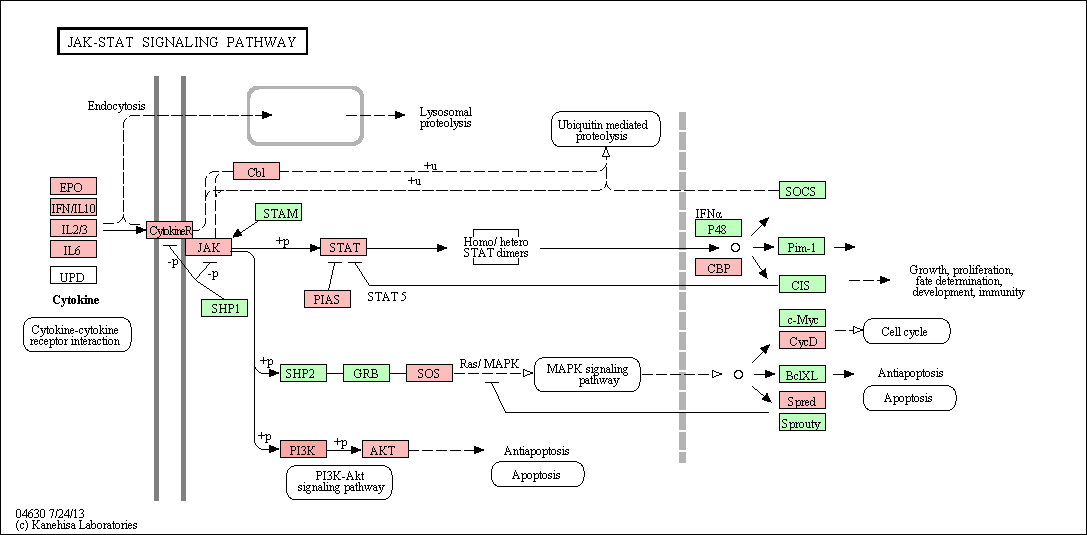
**

**Figure S37.** **Pathway collaboration of LuminalB - hsa04630: Jak-STAT signaling pathway.**

**
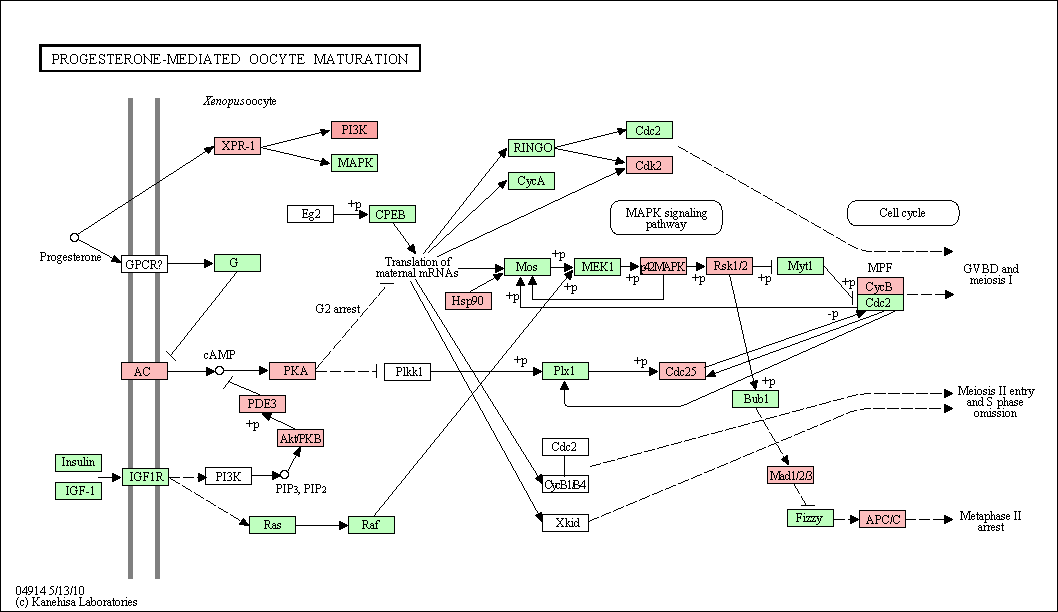
**

**Figure S38.** **Pathway collaboration of LuminalB - hsa04914: Progesterone-mediated oocyte maturation.**

**
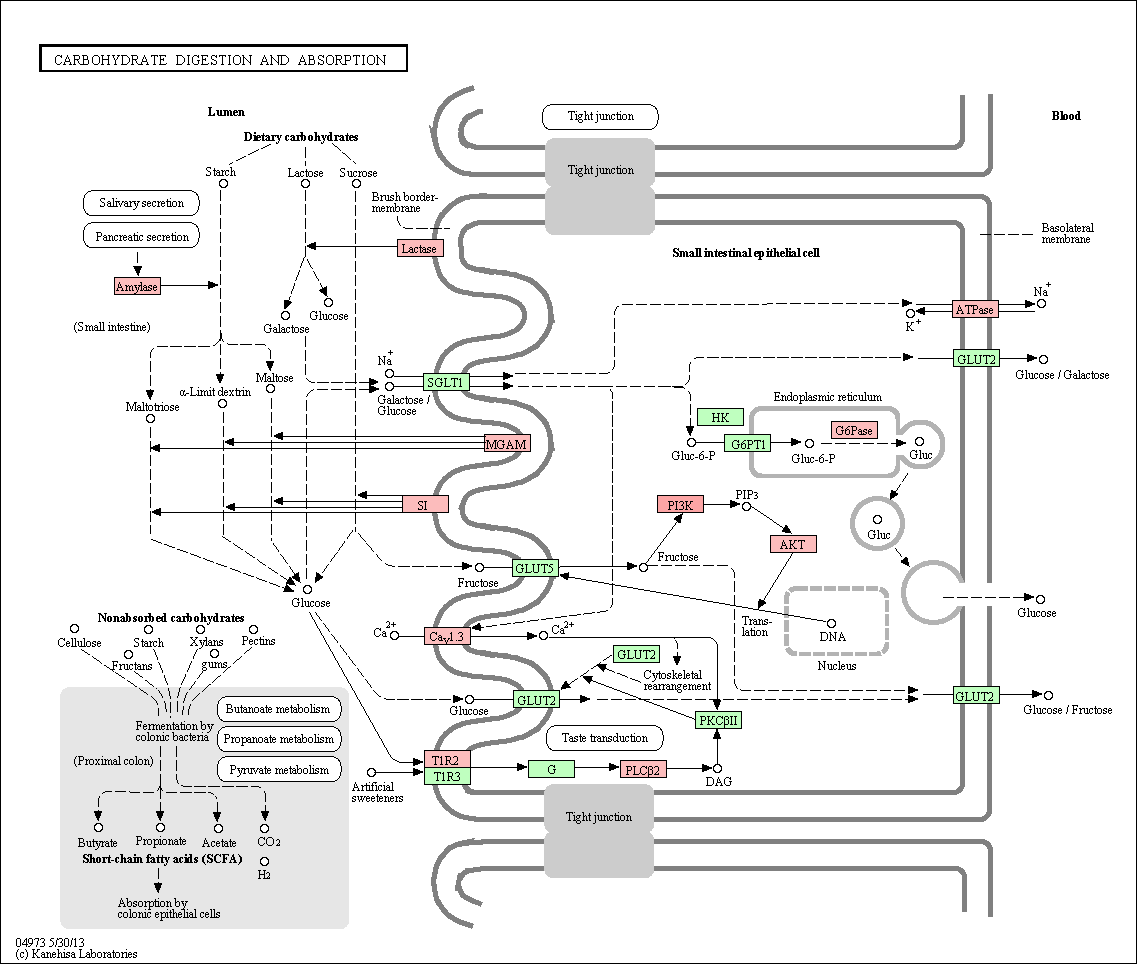
**

**Figure S39.** **Pathway collaboration of LuminalB - hsa04973: Carbohydrate digestion and absorption.**

**
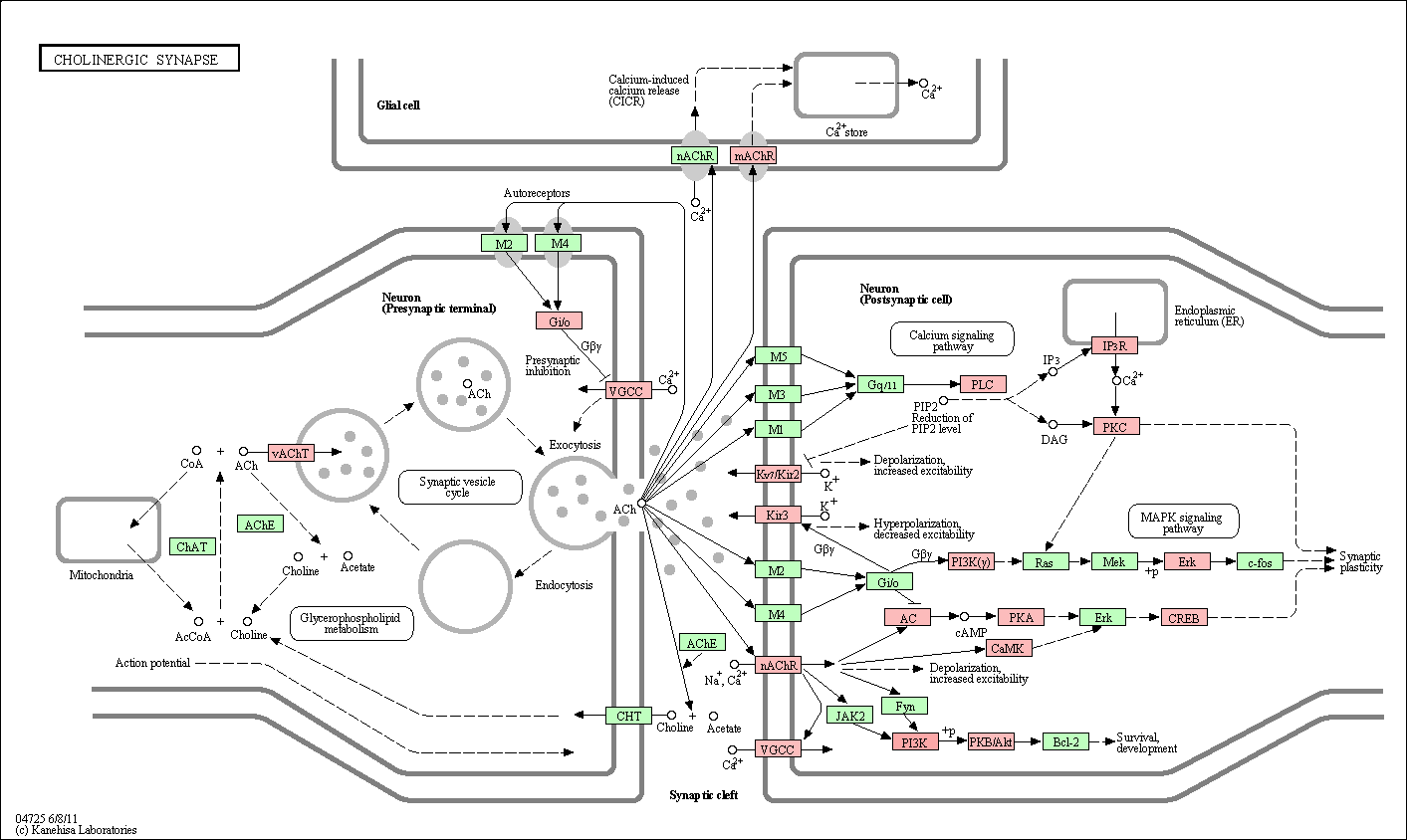
**

**Figure S40.** **Pathway collaboration of LuminalB - hsa04725: Cholinergic synapse.**

**
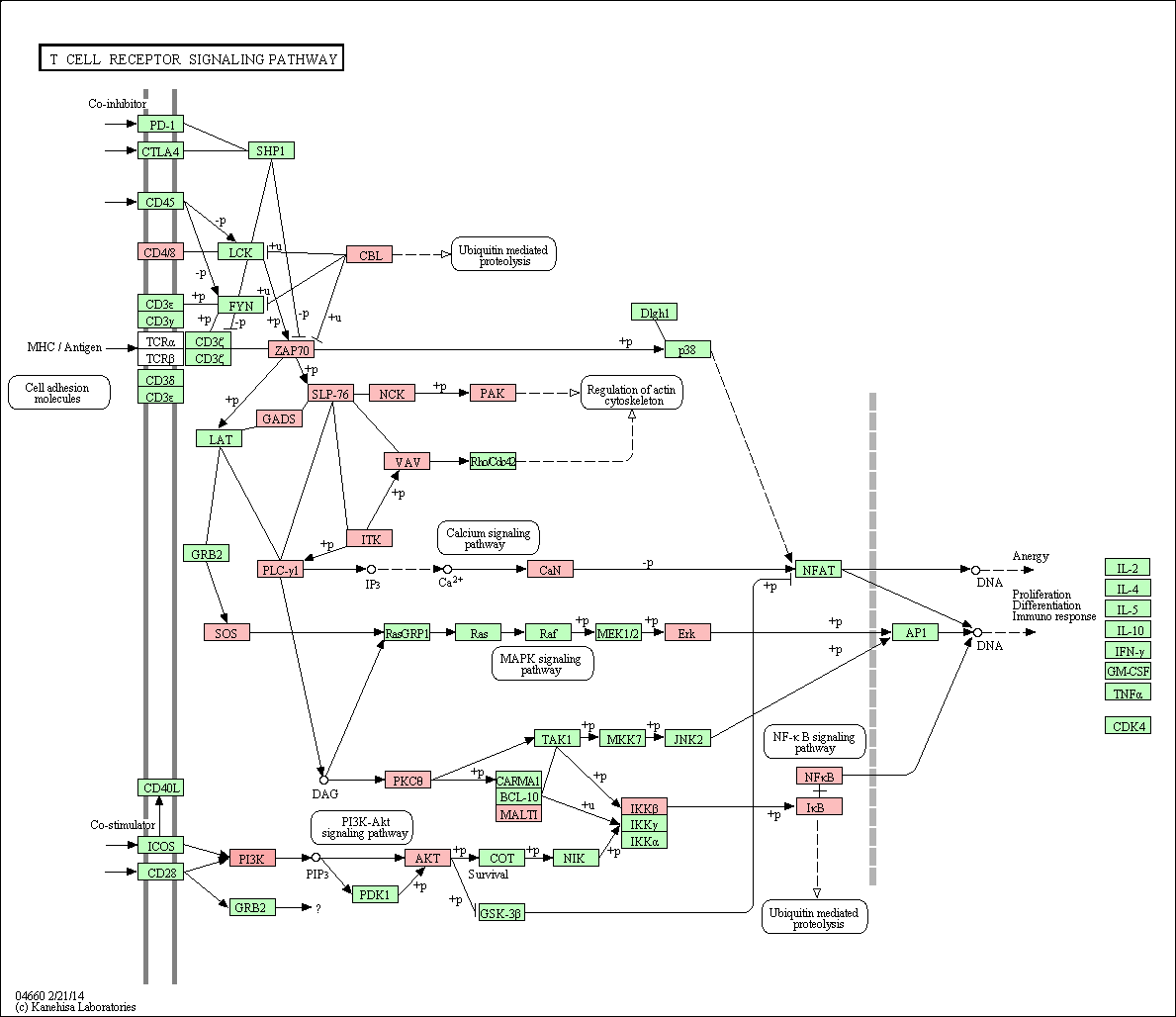
**

**Figure S41.** **Pathway collaboration of LuminalB - hsa04660: T cell receptor signaling pathway.**

**
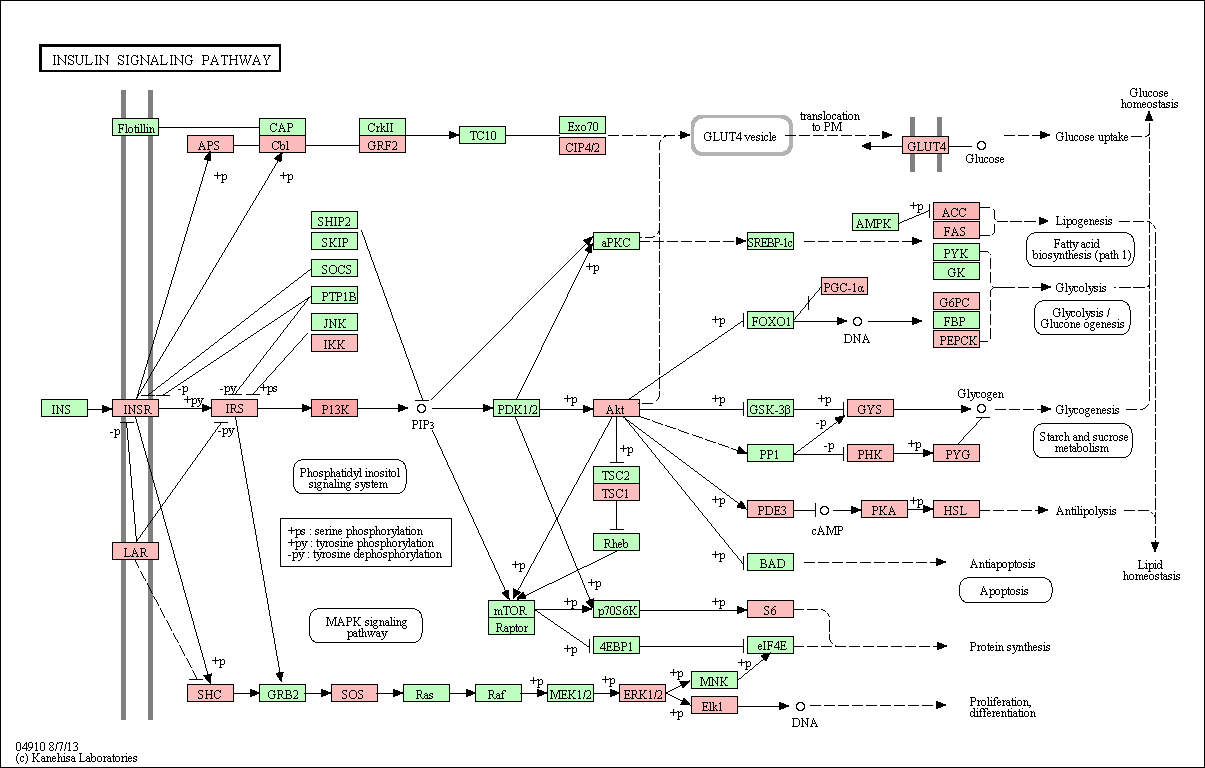
**

**Figure S42.** **Pathway collaboration of LuminalB - hsa04910: Insulin signaling pathway.**

**
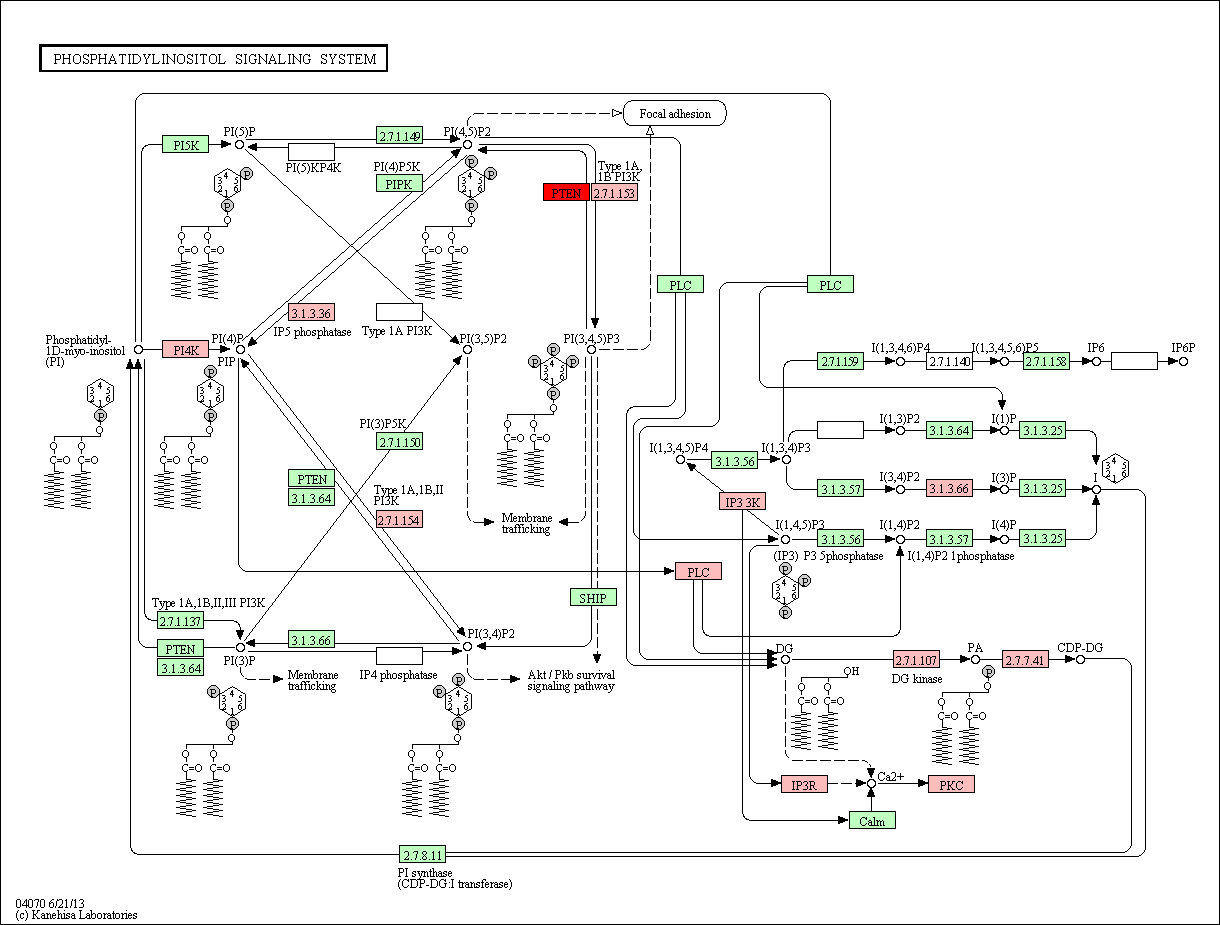
**

**Figure S43.** **Pathway collaboration of LuminalB - hsa04070: Phosphatidylinositol signaling system.**

**
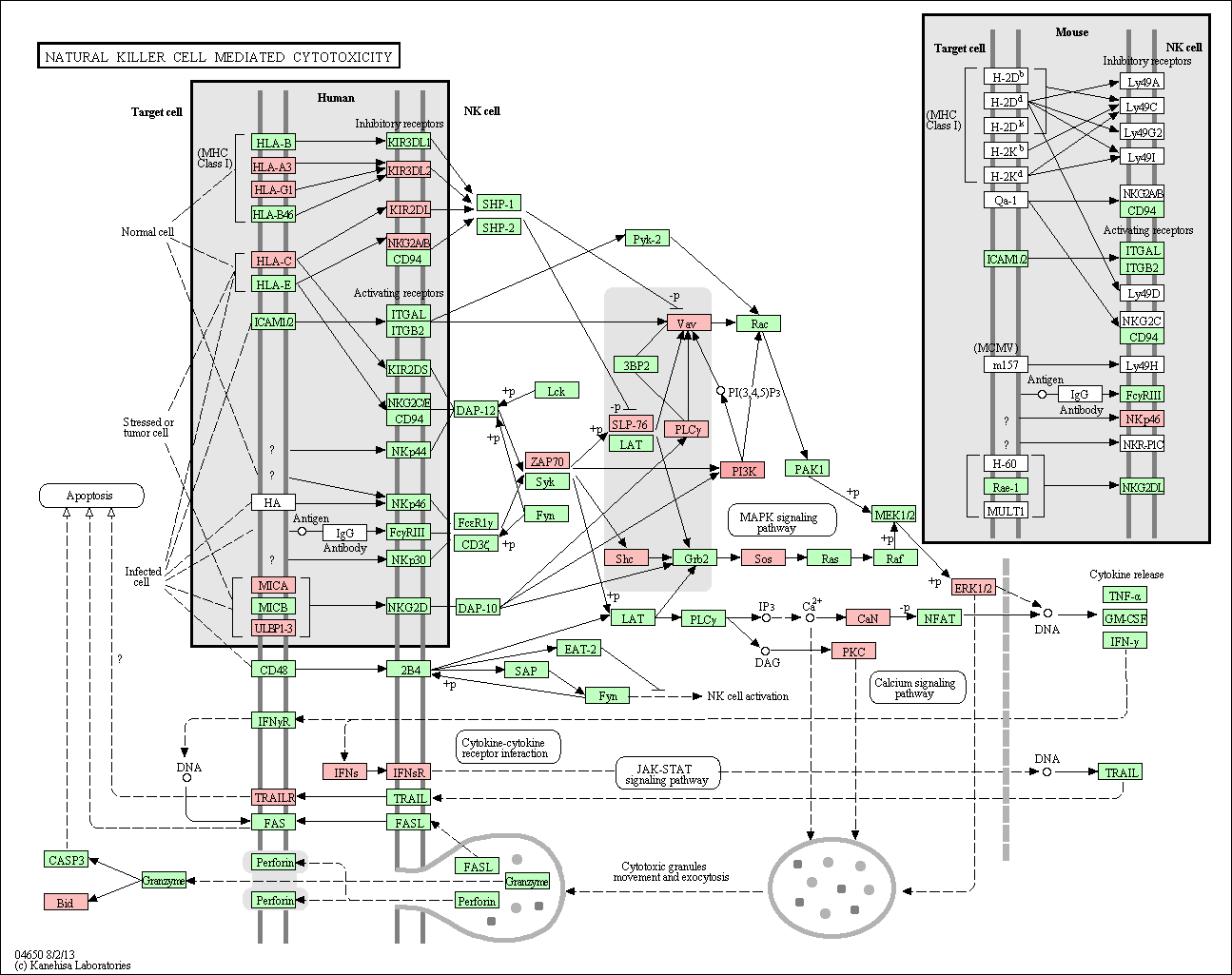
**

**Figure S44.** **Pathway collaboration of LuminalB - hsa04650: Natural killer cell mediated cytotoxicity.**

**Reference**

**1. Huang DW, Sherman BT, Lempicki RA: Systematic and integrative analysis of large gene lists using DAVID bioinformatics resources. *Nat Protoc* 2009, 4(1):44-57.**

**2. Huang DW, Sherman BT, Lempicki RA: Bioinformatics enrichment tools: paths toward the comprehensive functional analysis of large gene lists. *Nucleic Acids Res* 2009, 37(1):1-13.**

**3. Barretina J, Caponigro G, Stransky N, Venkatesan K, Margolin AA, Kim S, Wilson CJ, Lehar J, Kryukov GV, Sonkin D *et al*: The Cancer Cell Line Encyclopedia enables predictive modelling of anticancer drug sensitivity (vol 483, pg 603, 2012). *Nature* 2012, 492(7428):290-290.**

**4. Hua X, Xu HM, Yang YN, Zhu J, Liu PY, Lu Y: DrGaP: A Powerful Tool for Identifying Driver Genes and Pathways in Cancer Sequencing Studies. *Am J Hum Genet* 2013, 93(3):439-451.**
